# Disease trajectories following myocardial infarction: insights from process mining of 145 million hospitalisation episodes

**DOI:** 10.1016/j.ebiom.2023.104792

**Published:** 2023-09-21

**Authors:** Christopher J. Hayward, Jonathan A. Batty, David R. Westhead, Owen Johnson, Chris P. Gale, Jianhua Wu, Marlous Hall

**Affiliations:** a Clinical and Population Sciences Department, Leeds Institute of Cardiovascular and Metabolic Medicine, School of Medicine, Faculty of Medicine and Health, University of Leeds, Leeds, LS2 9JT, UK; b Leeds Institute for Data Analytics, University of Leeds, Leeds, LS2 9JT, UK; c School of Molecular and Cellular Biology, Faculty of Biological Sciences, University of Leeds, Leeds, LS2 9JT, UK; d School of Computing, Faculty of Engineering and Physical Sciences, University of Leeds, LS2 9JT, UK; e Department of Cardiology, Leeds Teaching Hospitals NHS Trust, Great George Street, Leeds, LS1 3EX, UK; f Wolfson Institute of Population Health, Queen Mary University of London, London, E1 4NS, UK

**Keywords:** Disease trajectories, Multimorbidity, Myocardial infarction, Electronic health records, Process mining, Machine learning

## Abstract

**Background:**

Knowledge of post-myocardial infarction (MI) disease risk to date is limited—yet the number of survivors of MI has increased dramatically in recent decades. We investigated temporally ordered sequences of all conditions following MI in nationwide electronic health record data through the application of process mining.

**Methods:**

We conducted a national retrospective cohort study of all hospitalisations (145,670,448 episodes; 34,083,204 individuals) admitted to NHS hospitals in England (1st January 2008–31st January 2017, final follow-up 27th March 2017). Through process mining, we identified trajectories of all major disease diagnoses following MI and compared their relative risk (RR) and all-cause mortality hazard ratios (HR) to a risk-set matched non-MI control cohort using Cox proportional hazards and flexible parametric survival models.

**Findings:**

Among a total of 375,669 MI patients (130,758 females; 34.8%) and 1,878,345 matched non-MI patients (653,790 females; 34.8%), we identified 28,799 unique disease trajectories. The accrual of multiple circulatory diagnoses was more common amongst MI patients (RR 4.32, 95% CI 3.96–4.72) and conferred an increased risk of death (HR 1.32, 1.13–1.53) compared with matched controls. Trajectories featuring neuro-psychiatric diagnoses (including anxiety and depression) following circulatory disorders were markedly more common and had increased mortality post MI (HR ranging from 1.11 to 1.73) compared with non-MI individuals.

**Interpretation:**

These results provide an opportunity for early intervention targets for survivors of MI—such as increased focus on the psychological and behavioural pathways—to mitigate ongoing adverse disease trajectories, multimorbidity, and premature mortality.

**Funding:**

10.13039/501100000274British Heart Foundation; 10.13039/100012338Alan Turing Institute.

**Research in context**

**Evidence before this study**

We searched the Ovid Medline database (1946–October 2022) and Embase (1996–October 2022) using medical Subject Heading (MeSH) terms “myocardial infarction”, or “acute coronary syndrome” combined with the terms “disease”, “co-morbidity”, or “multimorbidity” followed by “trajectory”, “sequence”, or “network” or one of “process” or “data” followed by “mining”. No language restrictions were applied. We searched for relevant grey literature using Google Scholar and Web of Science. Of the 160 results identified by this search, none systemically reported longitudinal multi-disease trajectories for individuals following myocardial infarction with the exception of Jensen et al. who studied pairs of conditions concatenated into trajectories in a Danish population of 6.2 million people in which MI trajectories were identified but without quantification of their impact on all-cause mortality; and Cezard et al. in which sequences of cardiovascular disease, diabetes, and cancer were studied amongst a select cohort of 6300 individuals from the Scottish population.

**Added value of this study**

This study comprehensively profiles sequential disease trajectories following myocardial infarction (MI) and assesses the differential impact of such trajectories on long-term outcomes. By evaluating a contemporary nationwide retrospective cohort of hospital admissions, and through the application of process mining, we identified distinct sequences of disease accrual that occur more frequently and confer highest mortality risk following hospitalisation for MI compared with matched individuals hospitalised for any other cause. Among 145 million hospitalisation episodes for 34 million adults admitted to hospitals across England, we observed 28,799 unique disease trajectories for survivors of MI. Trajectories involving the accrual of multiple circulatory diagnoses and those involving neuro-psychiatric diagnoses following circulatory disorders (including anxiety and depression) were between 4 and 35-fold more common and conferred between 11 and 73% increased risk of death compared with non-MI individuals. In combination with circulatory disorders, individuals were notably more likely to accrue respiratory and digestive conditions following MI but these trajectories were not associated with increased risk of death over and above that of the non-MI population.

**Implications of all the available evidence**

We provide a data-driven taxonomy of temporally ordered multisystem post-MI disease trajectories reflecting real-world pathways of disease from a comprehensive national electronic healthcare record dataset. The detailed multi-disease trajectories emerging from this study will inform more intensive and focussed research on key intervention points that have the potential to disrupt common and often fatal disease trajectories, improve patient quality of life, and reduce premature mortality associated with MI. Improved secondary prevention strategies to reduce the ongoing circulatory disease burden, as well as increased investigation of the physiological and behavioural pathways contributing to the neuro-psychiatric trajectories for survivors of MI, may help to mitigate the excess risks observed.

## Introduction

Improved survivorship following myocardial infarction (MI) has led to an increasing number of individuals vulnerable to the development of adverse health outcomes.^1^, ^2^, ^3^ This morbidity can include frequent readmission to hospital, the accrual of new disease states, reduction in quality of life, increased healthcare-associated costs, greater dependency, and premature mortality.^3^, ^4^, ^5^

Multimorbidity (the presence of two or more long-term health conditions) is common among patients with MI, confers a greater risk of mortality during both the initial MI hospitalisation and over the longer term, and presents a challenge to healthcare systems.^3^, ^4^, ^5^, ^6^ Although several studies report on the development of individual disease states following MI (including recurrent MI,^7^ heart failure,^8^ cancer,^8^ and dementia^9^), or cross-sectional clusters of multimorbidity,^3^ there are no studies to date that comprehensively describe the temporally-sequenced pattern of disease accrual for survivors of MI. Defining the course and progression of multiple diseases over time is crucial to determine early intervention points for the interruption or delay of the most fatal pathways.

In order to identify such disease trajectories—a generalised model of the likely sequence of related diseases that can be identified as patterns within a given population—longitudinal, individual-level datasets at scale are required which cover the full spectrum of diseases, alongside methodology that can handle the combinatorial growth from studying sequences of all disease types.^10^ The Hospital Episode Statistics (HES) data warehouse of all admissions to National Health Service (NHS) hospitals in England provide an opportunity to study disease accrual patterns for a ‘real world’ nationwide population across the full breadth of conditions.^11^ Whilst classical epidemiological methods, such as multistate models, are powerful for modelling known pathways of a limited number of disease states, they scale poorly beyond 10 or 15 conditions. Therefore, data-driven approaches, such as process mining,^12^ which do not rely on *a priori* knowledge of the order of occurrence of events, are crucial to the discovery of novel trajectories. Previous healthcare applications of process mining predominantly focussed on care pathways, with examples in oncology,^13^ diabetes,^14^ and stroke.^15^ Two prior studies focussed on pathways of disease including cardiovascular conditions—the first of these produced abstractions of generalised trajectories constructed from pairwise associations that did not preserve the observed pathways followed by individuals or assess their impact on outcomes^16^—the second study obtained sequences of cardiovascular disease but was limited to a select cohort of 6300 individuals from the Scottish population.^17^

We applied process mining to discover patient-level disease trajectories following MI in a nationwide cohort study of 145 million hospitalisation episodes for 34 million individuals across England. In doing so, we have established a taxonomy of post-MI temporal disease profiles, simultaneously capturing the combination of disease states, their temporal sequence, their relative risk, and association with long-term survival compared with non-MI individuals. Our results provide healthcare professionals—as well as patients and their carers—with detailed insight into the likely course of disease following MI. This study highlights the most pertinent early intervention points to interrupt disease pathways, reducing premature morbidity and mortality among the growing cohort of survivors of MI.

## Methods

This retrospective cohort study includes all individuals over 18 years of age in England admitted to hospital between 1st January 2008 and 31st January 2017.

### Data

Pseudonymised Admitted Patient Care data were obtained from the Hospital Episode Statistics (HES) data warehouse, including admissions to all NHS hospital trusts in England. HES data were linked to all-cause mortality records from the Office for National Statistics up to the final follow-up date (27th March 2017).

Each episode within HES constitutes a period of care under one consultant—a string of such episodes forms a full hospital admission. Within each episode, a single primary diagnosis and up to 19 secondary diagnoses are recorded and coded according to the International Classification of Disease version 10 (ICD-10). In addition to diagnoses, we extracted episode start and end dates, the order of episodes within admissions, admission and discharge dates, patient demographic details (age, sex, and the Index of Multiple Deprivation (IMD)^18^), and a hospital identifier for each admission (Table S1).

Access to data was governed by our data sharing agreement with NHS Digital (ref: NIC-17649) and privacy notice.^19^ Further ethical approval was not required for this study which solely relied on the secondary use of non-confidential healthcare data collected during the normal course of care. As part of legal requirement for data minimisation, data were pseudonymised and dates were aggregated to month and year. Furthermore, age and number of days survived were provided as derived fields rather than full date of birth and death respectively. Data minimisation was completed by NHS Digital prior to the release of data to the study team, in line with GDPR regulation.

### Cohort definition

**Fig. 1:**
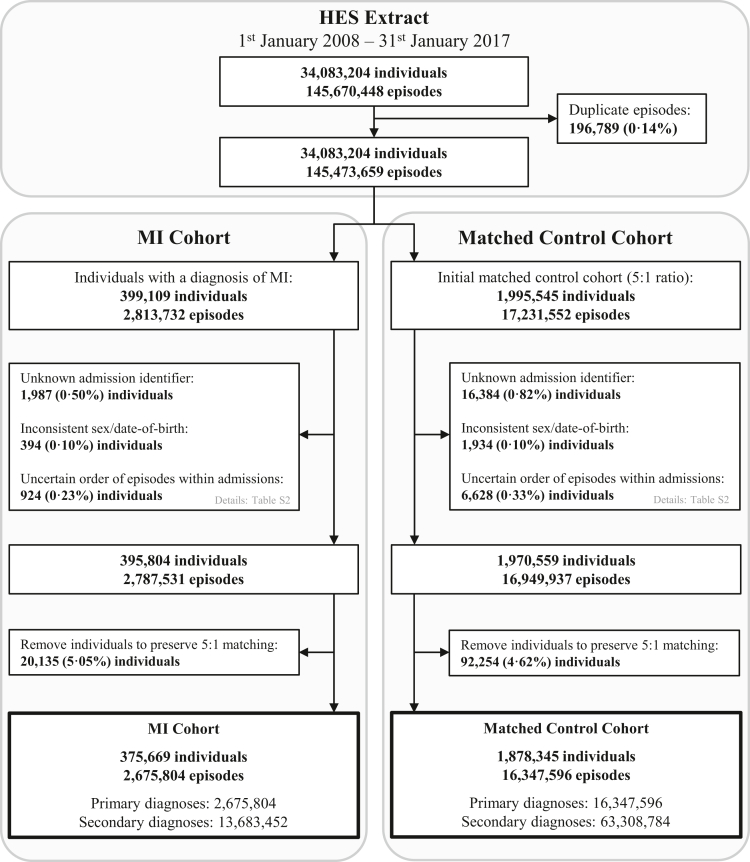
**Derivation of myocardial infarction (MI) and risk-set matched control cohorts from Hospital Episode Statistics (HES) hospitalisation episodes for adults over the age of 18 in England, 2008–2017**.

The primary analytical cohort included individuals hospitalised with MI at any point during the study period (ICD-10 codes I21, I22, or I23, occurring in the primary or any secondary diagnosis field) (Fig. 1). All individuals with MI recorded within HES prior to the study start date were excluded at source. A risk-set matched control cohort was defined, based on a 5:1 matching algorithm for single year of age, sex, month and year of admission, and NHS trust (Section S1).^20^ Individuals with missing data for age and sex were excluded from the matching risk set. For each individual, study entry date was defined as the first episode of MI or first matched hospitalisation for any other cause. The study exit date for an individual was either: (i) their date of death, (ii) the date of developing an MI for those in the matched comparator group, or (iii) 31st January 2017 (the censoring date).

### Statistical analyses

#### Disease trajectory definition

Disease trajectories were generated for all individuals in the MI and matched control cohorts through determining the sequence of disease occurrence based on the order of hospitalisation episodes. The concept of process mining relies on using timestamp information to infer an underlying process; we used the date associated with the start of each hospitalisation episode for a specific diagnosis to infer patterns of disease accrual.

Our primary analyses included sequences of primary diagnoses relating to the following broad disease groupings: infectious and parasitic diseases (ICD-10 chapter: A00-B99); neoplasms (C00-D48); diseases of the blood and blood-forming organs (D50-D89); endocrine, nutritional, and metabolic diseases (E00-E90); mental and behavioural disorders (F00-F99); diseases of the nervous system (G00-G99); eye and adnexa (H00-H59); ear and mastoid process (H60-H95); circulatory system (I00-I99); digestive system (K00-K93); skin and subcutaneous tissue (L00-L99); musculoskeletal system and connective tissue (M00-M99); and diseases of the genitourinary system (N00-N99) to form chronologically ordered disease trajectories for the MI and matched control cohorts. Diagnoses from any other ICD-10 chapter (non-disease hospitalisations) were excluded from trajectories. Secondary analyses incorporated both primary and secondary diagnoses into disease trajectories in the order that they appeared in each episode (Section S2). Additionally, to assess whether sex played a role in the outcomes associated with each trajectory, we performed analyses separately for males and females for both primary and secondary analyses. And finally, to determine the longer-term disease trajectory over and above the immediate health decline and/or the diagnostic investigations conducted in the short-term following MI, we conducted a landmark analysis restricted to hospitalisation episodes occurring more than six months after an individual's study entry date. For all analyses, chronic conditions were included in trajectories only if their first occurrence occurred after a first MI for the MI cohort, or after a matching date in the matched control cohort. Therefore, chronic conditions which first appeared prior to MI did not contribute to the post-MI disease trajectory for that individual. In contrast, acute conditions contributed to disease trajectories regardless of prior occurrence, and were additionally allowed to repeat within trajectories (Section S3). Trajectories consisted of sequences of three-character ICD-10 codes, before being aggregated and presented at the level of ICD-10 chapters (Section S4). The number of conditions that could contribute to any one trajectory was not predefined; the length of each trajectory was driven by the observed number of conditions per individual within the dataset.

For individuals with tied admissions—i.e. those occurring in same month and year for which the order could not be determined—a random order was assigned and its sensitivity checked with a different random order (individuals with at least one tied admission: MI—95,094 (25.3%); matched controls—289,956 (15.4%)).

Individuals whose trajectories could not be constructed due to invalid episode-order values and those with inconsistent recorded sex or date-of-birth were excluded from analyses (Table S2). Rare diagnoses—occurring in <0.1% of individuals within each cohort—were excluded from trajectories (Table S3).

#### Risk, all-cause mortality, and duration of disease trajectories

For all trajectories meeting our minimum inclusion threshold (occurring in at least 1 in 1000 MI individuals), we calculated a) the relative risk (RR) of the occurrence of a trajectory following MI vs. the matched control cohort, b) the likelihood of all-cause mortality per trajectory in the MI cohort compared with matched controls—presented as hazard ratios (HR), and c) the absolute difference in restricted mean survival time (RMST)^21^—i.e. the difference in follow-up time between the MI and matched control cohorts accounting for censoring—alongside 95% confidence intervals for the MI cohort compared with matched controls. RRs were calculated based on the number of individuals per trajectory among the MI and matched control groups, and HRs for all-cause mortality were derived using Cox proportional hazards mixed-effects models or flexible parametric survival models in the event of non-proportional hazards—based on the study entry and exit dates for each individual (see cohort definition section). Models were adjusted for age, sex, deprivation score, and year of admission for index hospitalisation, with a random intercept accounting for clustering at the NHS hospital trust level. Schoenfeld residuals were used to determine violation of the proportional hazards assumption,^22^ and choice of model scale and degrees of freedom for flexible parametric survival models was determined by minimising the Bayesian Information Criteria. To ensure sufficient statistical power, we modelled only those trajectories in which there were a minimum of 20 deaths per variable.

Analyses were conducted in Python (version 3.9) and R (version 4.0.2.) and data stored and processed within the Microsoft Azure-based Leeds Analytics Secure Environment for Research (LASER) Trusted Research Environment at the University of Leeds. Trajectories were generated using PM4Py version 2.2.11.1 (Section S5).^23^ Results are presented in the form: mean ± standard deviation for continuous data (or median [quartile 1, quartile 3], when skewed) and count (percentage) for categorical data. This study complies with RECORD^24^ and CODE-EHR^25^ reporting standards (Tables S4 and S5).

### Role of the funding source

This study was funded by the British Heart Foundation and Alan Turing Institute joint Cardiovascular Data Science Award (BHF-Turing-19/02/1022), and the Alan Turing Institute AI for Science and Government Funding (TU/ASG/R-SPEH-114). The study sponsors had no role in the study design, collection, analyses, or interpretation of data, in the writing of the report, or the decision to submit the manuscript for publication.

## Results

From 145,670,448 hospitalisation episodes for 34,083,204 individuals, we extracted a final cohort of 375,669 individuals with MI alongside their longitudinal causes for hospitalisation for inclusion in the analyses (N = 2,675,804 primary and 13,683,452 secondary diagnoses). A further 1,878,345 individuals were identified for inclusion in the matched control cohort (N = 16,347,596 primary and 63,308,784 secondary diagnoses) (Fig. 1). The median number of primary diagnoses per individual over the study period did not vary between cohorts (median [Q1,Q3]: 5 [3–9] and 5 [3–10] for the MI and matched control cohorts respectively; Figure S1).

Individuals in the MI cohort were older (67.6 ± 14.7 years) and more likely to be male (n = 244,911; 65.2%) compared with individuals in the full data extract (mean age 46.5 ± 36.5 years; 15,155,471 (44.5%) males). Exact matching achieved satisfactory balance in the matching variables between cohorts (mean age 67.6 ± 14.8 years; 1,224,555 (65.2%) males) (Table 1). Socioeconomic deprivation and overall follow-up time was similar in each cohort (IMD score: 17.6 [10.1–30.6] and 16.7 [9.8–29.3]; follow-up time: 3.6 [1.4–6.0] and 3.7 [1.7–6.1] years) and overall crude mortality was 27.3% (n = 102,533) vs. 24.9% (n = 467,791) for the MI and matched control cohorts respectively.

**Table 1:** Hospital Episode Statistics (HES) patient characteristics and hospitalisation episodes for adults over the age of 18 in England, 2008–2017.

	Full HES extract	Myocardial infarction (MI) cohort	Matched control cohort
Individuals, N	34,083,204	375,669	1,878,345
Episodes, N	145,670,448	2,675,804	16,347,596
Age at study entry (years), mean (SD)	46.5 (36.5)	67.6 (14.7)	67.6 (14.8)
Missing	770,761 (2.3)	0 (0)^a^	0 (0)^a^
Sex, n (%)			
Female	18,893,928 (55.4)	130,758 (34.8)	653,790 (34.8)
Male	15,155,471 (44.5)	244,911 (65.2)	1,224,555 (65.2)
Missing	33,805 (0.1)	0 (0)^a^	0 (0)^a^
Index of Multiple Deprivation (IMD), median [Q1-Q3]^b^	18.0 [10.1–31.4]	17.6 [10.1–30.6]	16.7 [9.8–29.3]
Missing	4,222,362 (12.4)	9684 (2.6)	29,052 (1.5)
All-cause mortality, n (%)	3,232,033 (9.5)	102,533 (27.3)	467,791 (24.9)
Missing	0 (0)	0 (0)	0 (0)
Study entry period, n (%)			
2008–2010	n/a	132,327 (35.2)	661,635 (35.2)
2011–2013	n/a	132,520 (35.3)	662,600 (35.3)
2014–2017	n/a	110,822 (29.5)	554,110 (29.5)
Missing	n/a	0 (0)^a^	0 (0)^a^
Follow-up duration (from study entry for cohorts) (years), median [Q1-Q3]	5.2 [2.7–7.3]	3.6 [1.4–6.0]	3.7 [1.7–6.1]
Number of primary diagnoses per individual, median [Q1-Q3]	2 [1–4]	5 [3–9]	5 [3–10]
Infectious/Parasitic (A00-B99)			
Primary N (%)	2,713,174 (1.9)	28,906 (1.1)	234,684 (1.4)
Secondary N (%)	7,204,543 (1.6)	147,448 (1.1)	980,636 (1.5)
Neoplasms (C00-D48)			
Primary N (%)	18,300,517 (12.6)	139,194 (5.2)	2,896,865 (17.7)
Secondary N (%)	15,214,641 (3.4)	159,028 (1.2)	2,513,841 (4.0)
Blood/Immune (D50-D89)			
Primary N (%)	2,531,850 (1.7)	41,498 (1.6)	378,420 (2.3)
Secondary N (%)	6,539,687 (1.5)	166,914 (1.2)	976,839 (1.5)
Endocrine/Nutritional/Metabolic (E00-E90)			
Primary N (%)	2,223,783 (1.5)	26,177 (1.0)	321,327 (2.3)
Secondary N (%)	37,049,390 (8.3)	1,215,564 (8.9)	5,582,965 (8.8)
Mental/Behavioural (F00-F99)			
Primary N (%)	1,842,684 (1.3)	13,107 (0.5)	174,138 (1.1)
Secondary N (%)	24,999,313 (5.6)	562,898 (4.1)	3,015,626 (4.8)
Nervous System (G00-G99)			
Primary N (%)	3,091,760 (2.1)	33,098 (1.2)	366,347 (2.2)
Secondary N (%)	9,267,219 (2.1)	174,699 (1.3)	1,384,967 (2.2)
Eye/Adnexa (H00-H59)			
Primary N (%)	5,095,811 (3.5)	85,583 (3.2)	741,855 (4.5)
Secondary N (%)	5,096,489 (1.1)	104,064 (0.8)	810,651 (1.3)
Ear/Mastoid Process (H60-H95)			
Primary N (%)	507,956 (0.3)	4498 (0.2)	36,929 (0.2)
Secondary N (%)	1,621,664 (0.4)	38,649 (0.3)	248,787 (0.4)
Circulatory System (I00-I99)			
Primary N (%)	11,047,088 (7.6)	1,137,804 (42.5)	1,602,711 (9.8)
Secondary N (%)	72,641,270 (16.4)	4,792,692 (35.0)	12,033,739 (29.3)
Respiratory System (J00-J99)			
Primary N (%)	9,216,649 (6.3)	186,087 (7.0)	1,227,509 (7.5)
Secondary N (%)	24,537,038 (5.5)	734,023 (5.4)	3,560,776 (5.6)
Digestive System (K00-K93)			
Primary N (%)	16,446,316 (11.3)	212,102 (7.9)	1,814,935 (11.1)
Secondary N (%)	23,693,961 (5.3)	487,871 (3.6)	3,372,573 (5.3)
Skin/Subcutaneous Tissue (L00-L99)			
Primary N (%)	2,854,713 (2.0)	31,578 (1.2)	304,392 (1.9)
Secondary N (%)	5,145,423 (1.2)	115,260 (0.8)	759,289 (1.2)
Musculoskeletal System (M00-M99)			
Primary N (%)	10,558,860 (7.2)	127,314 (4.8)	1,175,278 (7.2)
Secondary N (%)	20,360,936 (4.6)	485,328 (3.5)	3,032,039 (4.8)
Genitourinary System (N00-N99)			
Primary N (%)	14,005,732 (9.6)	131,076 (4.9)	1,874,474 (11.5)
Secondary N (%)	18,387,692 (4.2)	457,439 (3.3)	2,847,848 (4.5)

a Zero missing data by design—individuals with missing data in core matching fields were excluded prior to the matching process.

b For the full HES extract: data correspond to the individual's earliest recorded episode; for the cohorts: data correspond to the episode on the matched date.

### Post-MI disease trajectories

**Fig. 2:**
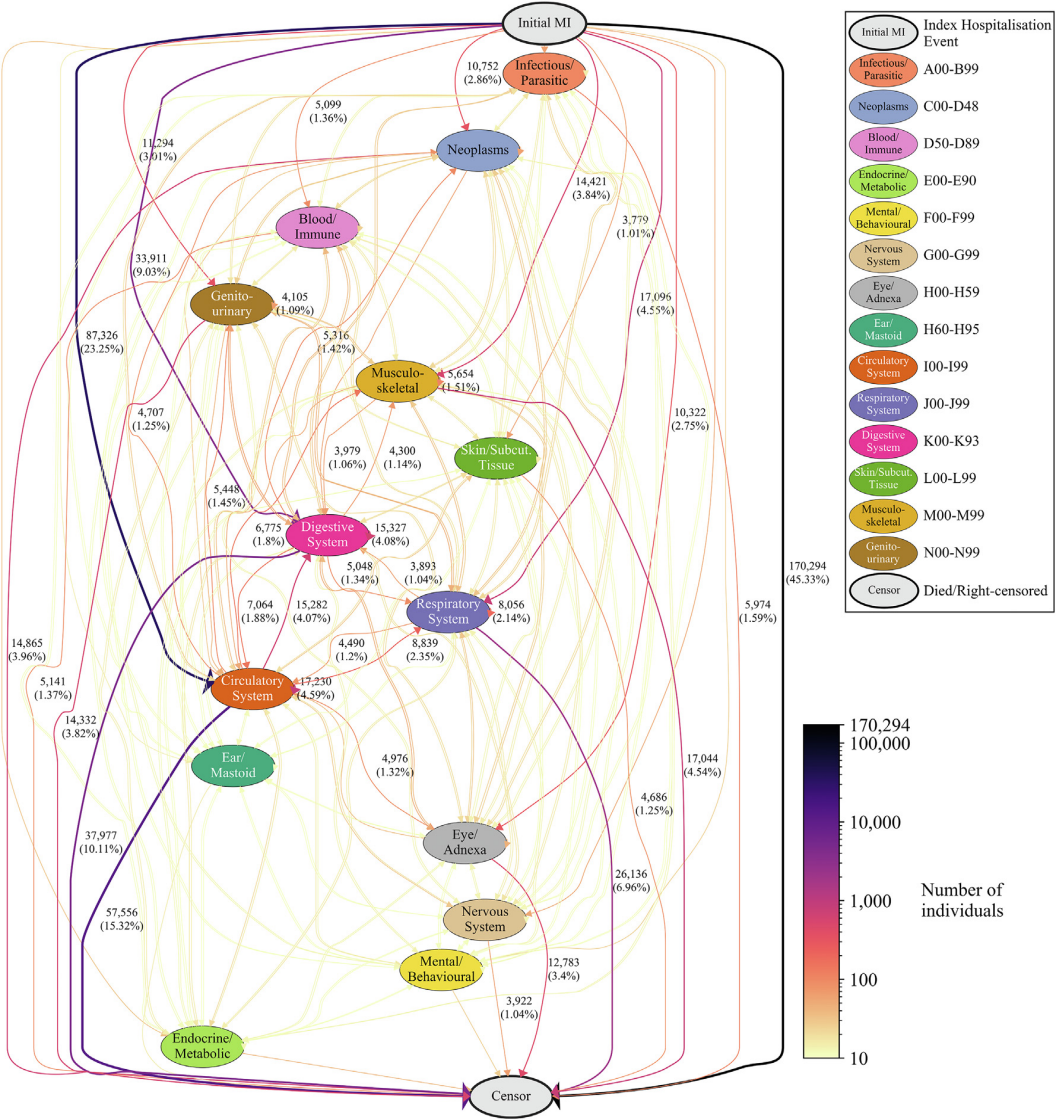
**The network of disease transitions for temporally sequenced diseases following myocardial infarction (MI), for adults with MI in England, 2008–2017**. Directed edges between chapter headings represent progression of individuals from one primary diagnosis to the next, grouped by ICD-10 chapters. Disease trajectories from all individuals within the MI cohort (n = 375,669) are used to construct this network. The number of individuals (and percentage of cohort) who progress from one disease chapter to the next is shown when at least 1% of individuals contribute to that progression. Edge colour (ref: colour-bar) and thickness are proportional to the number of individuals that progress from one chapter to another. Index Hospitalisation Event is defined as the first primary diagnoses (MI) at the time of matching. An interactive version of this figure—showing counts for all connections; the network for the secondary analyses; and granular sub-chapter transitions—is available at https://multimorbidity-research-leeds.github.io/.

We discovered 28,799 and 194,913 unique disease trajectories following MI for the primary and secondary analyses respectively, forming a network of transitions between disease states (Fig. 2, Figure S2; view interactively via https://multimorbidity-research-leeds.github.io/). Post-MI trajectories ranged between zero and 29 diagnoses in length in the primary analyses, and between zero and 134 diagnoses in the secondary analyses (Table S6). The most commonly featured primary diagnoses post MI related to circulatory, digestive, and respiratory diseases (present in 17,946 (62.3%), 16,831 (58.4%), and 12,766 (44.3%) trajectories, respectively) (Table S7); the most frequent post–MI diagnoses were chronic ischaemic heart disease (36,220; 7.3%), pneumonia (24,623; 5.0%), and angina pectoris (18,675; 3.8%), compared with pneumonia (143,663; 5.1%), urinary tract infection (106,522; 3.8%), and ‘other cataract’ (71,624; 2.5%) for the matched control cohort (Table S8).

**Fig. 3:**
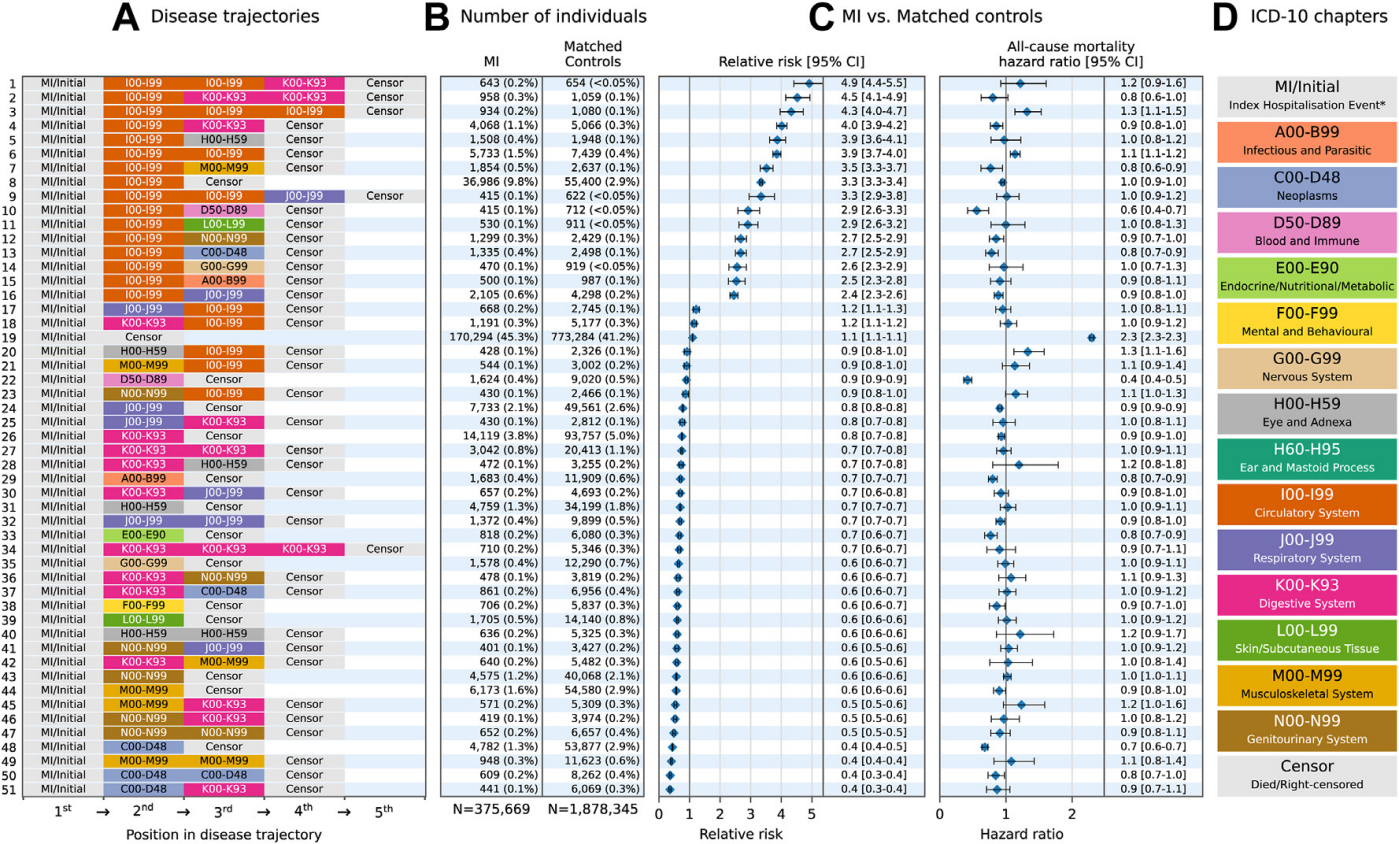
**Disease trajectories† of primary hospitalisation cause for adults with myocardial infarction (MI) in England, compared with an age, sex, and year matched control cohort, 2008–2017**. **A:** Chronological disease trajectories including primary diagnoses codes according to ICD-10 chapter headings, ordered by relative risk. **B:** Number of MI cases and matched controls following each trajectory. **C:** The relative risk and 95% confidence intervals of disease trajectories between MI and matched control cases, and hazard ratios—all-cause mortality calculated using time-to-event models adjusted for age, sex, year, and deprivation, accounting for cases nested within hospital trusts using random effects. **D:** ICD-10 chapter headings. An interactive version of this figure—showing a breakdown of disease counts within chapters—is available at https://multimorbidity-research-leeds.github.io/. The RMST for each trajectory is available in Figure S7. ∗Index Hospitalisation Event refers to the initial MI diagnoses for the MI cohort and the first primary diagnoses at the time of matching for the matched control cohort. †Only disease trajectories which were followed by at least 0.1% of the MI cohort are shown.

**Fig. 4:**
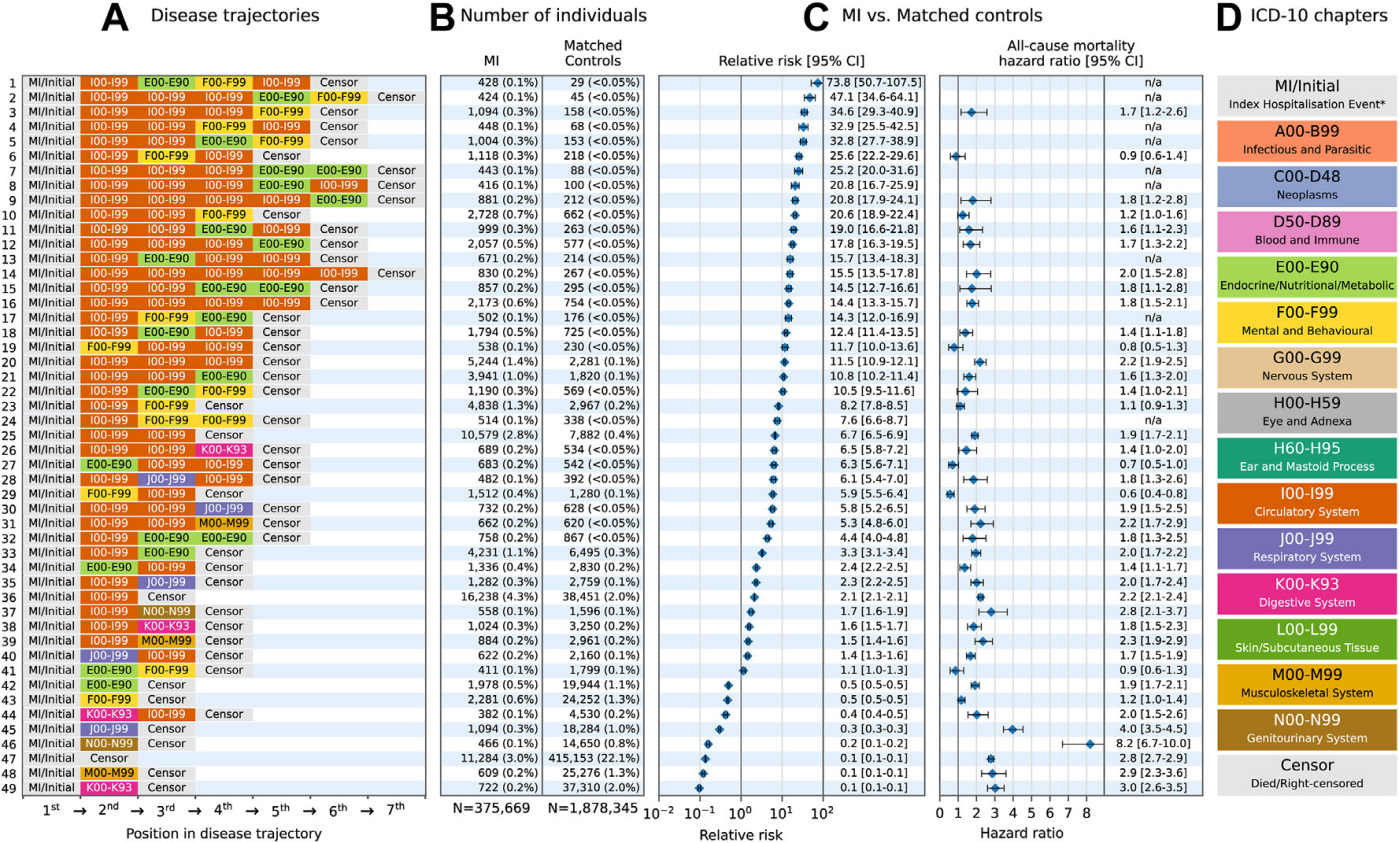
**Disease trajectories† of primary and secondary hospitalisation cause for adults with myocardial infarction (MI) in England, compared with an age, sex, and year matched control cohort, 2008–2017**. **A:** Chronological disease trajectories including primary and secondary diagnoses codes according to ICD-10 chapter headings, ordered by relative risk. **B:** Number of MI cases and matched controls following each trajectory. **C:** The relative risk and 95% confidence intervals of disease trajectories between MI and matched control cases, and hazard ratios—all-cause mortality calculated using time-to-event models adjusted for age, sex, year, and deprivation, accounting for cases nested within hospital trusts using random effects. Hazard ratios are missing (n/a) where the number of deaths fell below the events-per-variable threshold, or too few cases existed for the model to converge. **D:** ICD-10 chapter headings. An interactive version of this figure—showing a breakdown of disease counts within chapters—is available at https://multimorbidity-research-leeds.github.io/. The RMST for each trajectory is available in Figure S11. ∗Index Hospitalisation Event refers to the initial MI diagnoses for the MI cohort and the first diagnoses at the time of matching for the matched control cohort. †Only disease trajectories which were followed by at least 0.1% of the MI cohort are shown.

A total of 51 and 49 disease trajectories—for the primary and secondary analyses respectively—met the minimum inclusion threshold for further analyses (observed in 296,902 (79.0%) and 96,631 (25.7%) MI patients respectively; Figs. 3 and 4; view interactively via https://multimorbidity-research-leeds.github.io/).

#### Multiple circulatory diseases

There was an increased risk of hospitalisation with multiple circulatory diseases following an initial MI (RR: 3.85 [3.72–3.99] and 4.32 [3.96–4.72] for two and three circulatory diseases, respectively) which also conferred an increased risk of death (HR: 1.14 [1.07–1.21] and 1.32 [1.13–1.53] respectively) compared with matched controls (Fig. 3). The increased risk of death for multiple circulatory diagnoses following MI remained in the landmark analyses (HR: 1.24 [1.14–1.35]) (Figure S3), and in the sensitivity analysis implementing alternative ordering for tied admissions (HR: 1.14 [1.07–1.21] and 1.34 [1.16–1.56] for two and three circulatory diagnoses, respectively) (Figure S4).

When stratifying the primary analysis by sex, males and females displayed similar increases in risk of death for multiple circulatory diagnoses following MI—trajectories consisting of two and three circulatory disorders following MI were associated with a 7% and 35% increase in risk of death for males (HR: 1.07 [0.98–1.17]; 1.35 [1.11–1.63]), and a 21% and 34% increase in risk of death for females (HR: 1.21 [1.10–1.33]; 1.34 [1.06–1.71]), respectively (Figures S5 and S6).

#### Circulatory, digestive system, and respiratory diseases

There was an increased risk of post-MI trajectories containing circulatory diseases followed by diseases of the digestive system (RR: 4.02 [3.85–4.18] to 4.92 [4.41–5.48]), and circulatory diseases followed by respiratory diseases (RR: 2.45 [2.32–2.58] to 3.34 [2.95–3.78]) compared with matched controls. Reduced mortality was observed in individuals with MI who followed {MI/initial→circulatory→digestive→censor} (HR: 0.85 [0.77–0.95]) with no significant difference for {MI/initial→circulatory→circulatory→digestive→censor} (HR: 1.22 [0.92–1.61]). For post-MI trajectories containing circulatory and digestive diseases, the most common digestive system diagnoses were gastritis and duodenitis (n = 792; 10.1% of digestive diagnoses), inguinal hernia (n = 612; 7.8%), and diverticular disease of the intestine (n = 578; 7.4%).

There was no consistent difference in the risk of death for those with circulatory and respiratory diseases between the MI and matched control cohort. The most common post–MI respiratory conditions related to pneumonia (1418; 20.9% of respiratory diagnoses), unspecified acute lower respiratory infection (602; 8.9%), and other chronic obstructive pulmonary disease (319; 4.7%).

#### Circulatory and ‘eye and adnexa’ diseases

Following MI, diagnoses of the eye and adnexa predominantly included ‘other cataract’ (n = 3779; 44.8% of eye/adnexa diagnoses) and senile cataract (n = 2860; 33.9%) diagnoses. Although the {MI/initial→circulatory→eye/adnexa→censor} trajectory was more common post MI compared with matched controls (RR: 3.87 [3.62–4.14]), only the {MI/initial→eye/adnexa→circulatory→censor} trajectory led to an increased risk of death (HR: 1.33 [1.12–1.58]), and a reduced RMST (MI: 6.55 [6.27–6.84] years; matched controls: 6.89 [6.77–7.02]) (Figure S7). The most common circulatory diagnoses for {MI→eye/adnexa→circulatory→censor} were cerebral infarction (70; 16.4% of circulatory diagnoses) and heart failure (62; 14.5%).

#### Cancer, digestive, musculoskeletal, and genitourinary diseases

Disease trajectories with cancer, digestive, musculoskeletal, and genitourinary diseases—but not containing a further circulatory disease—occurred less frequently in the post-MI cohort. These trajectories had a mixed but significant effect on survival, including a 32% reduced risk of death for the {MI/initial→cancer→censor} trajectory (RR: 0.44 [0.43–0.46]; HR: 0.68 [0.64–0.73]), and 16% reduced risk of death for the {MI/initial→cancer→cancer→censor} trajectory (RR: 0.37 [0.34–0.40]; HR: 0.84 [0.73–0.98]) compared with matched controls. Although trajectories including two musculoskeletal or two genitourinary conditions were 59% and 51% less likely in the MI cohort respectively, there was no observed difference in survival.

#### ‘Infectious and parasitic’ and ‘blood and immune’ diseases

Trajectories of a single ‘infectious and parasitic’ or ‘blood and immune’ disease were associated with reduced mortality in the MI cohort, particularly so for {MI/initial→blood/immune→censor} which had a 58% reduced risk of death for the MI cohort (RR: 0.90 [0.85–0.95]; HR: 0.42 [0.36–0.48]). The most common post-MI blood/immune diagnoses for this trajectory were iron deficiency anaemia (232; 55.9%) and other anaemia (124; 32.3%)—these diagnoses were also common in the matched controls: 314 (44.1%) and 255 (35.8%), respectively.

#### Mental and behavioural disorders

Post-MI trajectories involving multiple circulatory diseases and mental and behavioural disorders became evident in the secondary analyses. Trajectories containing circulatory diseases prior to mental disorders occurred more frequently and conferred an increased risk of death post MI (RR: 8.15 [7.79–8.53], 20.60 [18.93–22.43], 34.62 [29.30–40.90] and HR: 1.11 [0.92–1.34], 1.25 [0.98–1.59], 1.73 [1.16–2.58] for one, two, and three circulatory diseases followed by a mental and behavioural disorder respectively) (Fig. 4). Chronic ischaemic heart disease (6954; 51.2%), hypertension (1592; 11.7%), and heart failure (954; 7.0%); and mental and behavioural disorders due to tobacco use (7681; 88.7%), depressive episodes (282; 3.3%), and other anxiety disorders (201; 2.3%) were the three most common diagnoses amongst the circulatory and mental health conditions within these trajectories, respectively (view interactively via https://multimorbidity-research-leeds.github.io/). The increased risk of death post MI for individuals with trajectories containing circulatory diseases followed by mental and behavioural disorders remained in the sensitivity analysis implementing alternative ordering for tied admissions (HR: 1.13 [0.94–1.36], 1.23 [0.96–1.57], 1.72 [1.15–2.56] for one, two, and three circulatory diseases followed by a mental and behavioural disorder respectively) (Figure S8).

Additionally, the elevated risk of death for multiple circulatory diseases followed by a mental/behavioural disorder post MI remained when analysing males and females separately, however females had a greater risk of death compared to males—for two circulatory diseases followed by a mental/behavioural disorder, the increase in risk of death post MI for females was 62% (HR: 1.62 [1.00–2.60]) compared to approximately 21% for males (HR: 1.21 [0.86–1.71]) (Figures S9 and S10).

#### Endocrine, nutritional, and metabolic diseases

Trajectories including circulatory and ‘endocrine, nutritional, and metabolic’ diseases also become more evident when including secondary diagnoses in the analyses, and were more common (RR: 2.36 [2.21–2.52] to RR: 73.79 [50.66–107.48]) with risk of death ranging from a reduction of 30% to an increase of 97% (HR: 0.70 [0.48–1.02] to HR: 1.97 [1.74–2.23]) for MI vs. matched controls.

## Discussion

In this study, a data-driven, process mining-based approach was used to identify and describe 28,799 unique longitudinal disease trajectories experienced by individuals hospitalised with MI in England. Our results highlight the 4-fold increased risk of hospitalisation with further multiple circulatory conditions for survivors of their first MI, associated with a 32% increased risk of death compared with matched non-MI controls. Trajectories containing circulatory diseases paired with digestive or respiratory conditions were notably more frequent following MI than amongst non-MI individuals with between 15% and 4.9-fold increased risk, however, despite occurring more frequently they did not confer an increased risk of all-cause mortality over and above the same trajectories amongst the non-MI population. Notably, after MI, there was between an 8- and 35-fold increase in the risk of neuro-psychiatric diagnoses (including anxiety and depression) following multiple circulatory disorders, which conferred between 11 and 73% increased mortality compared with the non-MI population.

The results of this study identified risk of hospitalisation with further circulatory conditions following MI, similar to the cardiovascular trajectories observed in the Danish population.^16^ Our study further quantifies both the increased likelihood (3.9-fold and 4.3-fold) and increased risk of death (14% and 32%) of two and three subsequent circulatory diagnoses following MI compared with matched individuals without MI. Greater risk of accruing additional cardiovascular disease states may be due to either a direct effect of the infarct (e.g., cardiac dysfunction, and remodelling increasing the risk of atrial fibrillation and heart failure) or due the presence of shared underlying cardiovascular risk factors (e.g., smoking, hyperlipidaemia, and hypertension, which predispose an individual to developing extra-cardiac arterial disease). That these trajectories were observed despite the widespread use of contemporary, guideline-directed secondary prevention drugs suggests that more aggressive risk factor modification and new secondary prevention pharmaco-therapeutic strategies may be required to prevent the accrual of further cardiovascular disease and reduce premature mortality in the post-infarct period.

The emergence of trajectories including gastrointestinal diagnoses following MI may be attributed in part to the erosive side-effects of secondary prevention medication, including antiplatelet agents.^26^ Indeed, these trajectories were characterised by a high prevalence of gastritis and duodenitis which are recognised side effects of aspirin therapy.^27^ The gastrointestinal sequelae of MI treatment may be prevented by the use of gastro-protective agents (such as proton-pump inhibitors) in patients receiving dual antiplatelet therapy, as recommended in recent guidelines.^28^

Notably, there was a significant burden of neuro-psychiatric diagnoses (driven by smoking, depression, and anxiety) following MI and multiple additional circulatory disorders, with significant impact on all-cause mortality over and above the matched non-MI patients. These findings extend previous efforts describing disease accrual following cardiovascular disorders by highlighting mental health disorders and risk of death post MI.^16^^,^^17^ Although several studies report increased prevalence of depression and related mental health conditions following MI, estimates of prevalence vary widely and are predominantly based on small select cohorts.^29^^,^^30^ A number of mechanisms for the development of depression following MI have been proposed, including dysregulation of coping mechanisms after a major adverse life event, and shared underlying risk factors including greater socioeconomic deprivation. Depression is shown to reduce secondary prevention medication adherence and engagement with cardiac rehabilitation and is therefore a major target for treatment to improve quality of life and patient outcomes. Further, the elevated risk of death for females compared with males observed in this study suggests the need for closer surveillance for females experiencing circulatory and neuro-psychiatric disorders after MI.

Several trajectories appeared less frequently in the MI cohort and conferred a reduced risk of death compared with controls, including trajectories that included cancer, and those with blood and immune diseases. The effects of post-MI aspirin therapy may explain the reduced risk of developing cancer following an MI.^31^ The reduced risk of death for trajectories with blood and immune diseases—composed primarily of anaemia in both MI and non-MI cohorts—may be due to patients receiving antiplatelet agents with anaemia receiving more intensive investigation and management for anaemia. Despite occurring more frequently in the MI cohort compared with controls, trajectories with a circulatory disease followed by a digestive disorder also indicated a reduced risk of death compared with controls, an observation which may be mediated by post-MI medication, increased investigation and greater-intensity management.

This study identifies and characterises specific patterns of disease trajectories that: (i) occur more frequently, and (ii) are associated with increased risk of all-cause mortality following MI compared with matched non-MI controls. In contrast with previous approaches—including the work by Jensen et al. who concatenated pairs of disease transitions from multiple patients into generalised trajectories for 6.2 million patients of the Danish population—our study utilises the observed sequences of diseases accrued amongst individual patients, providing insights into the specific disease burden following MI on a nationwide scale.^16^ The reported frequency of occurrence and risk of death associated with these trajectories provides crucial information for healthcare professionals and policy makers, acting as a foundation to inform clinical practice. Our findings offer an insight into the future health needs for this multimorbid population and suggest targets for intervention for the most fatal pathways. Our analysis also indirectly demonstrates the importance of new and improved preventative strategies for MI—by preventing MI in an individual, they are less likely to undergo a trajectory of subsequent hospitalisations with a greater burden of cardiovascular morbidity and greater risk of mortality.

Process mining is capable of discovering sequenced trajectories based on discrete time event data such as hospitalisation records, making it a powerful discovery tool that can feed into subsequent epidemiological methods that require *a priori* knowledge of the likely order of events. Future work could consider multi-state modelling for the most fatal trajectories to further understand disease progression following MI and the factors that influence transitions from one condition to the next.

There are several study limitations. Firstly, the pseudonymised nature of the data meant that dates of diagnoses were only available at month and year granularity, resulting in a small number of individuals for whom the true sequence of disease could not be deciphered. Moreover, due to the observational cohort design, our dataset covers a defined calendar period (2008–2017) with individuals entering the cohort at point of index MI, and as such follow-up time per individual varies which can impact upon the trajectories observed. Whilst our survival analyses account for differential follow-up time because of censoring, our relative risk analyses did not. We are assured however that the impact of this on our analyses was likely small, given that we observed minimal differences in restricted mean survival time between the MI and matched control cohorts. Secondly, whilst we were able to identify individuals with index MI within our study period—given that individuals with MI prior to the study start date were removed at source (by NHS Digital)—we did not have access to data on possible baseline conditions for the full breadth of ICD-10 diagnoses prior to the study start in 2008. Whilst we have minimised bias from pre-existing co-morbidities on the resulting trajectories as far as possible by only including chronic conditions whose first occurrence appear after index MI, we acknowledge that we were unable to account for historical diagnoses as a limitation, with potential impact on the resulting trajectories.

Regarding further study limitations, all secondary diagnoses are attributed to the same date as the parent hospital episode due to the structure of HES data. Nonetheless, our sensitivity analyses showed consistent results with the primary findings, and the inclusion of secondary diagnoses revealed important patterns of neuro-psychiatric diagnoses following MI with increased risk of mortality which could not be observed through primary diagnoses alone. Furthermore, whilst the study excluded all patients with a prior diagnosis of MI, we do not have data on diagnoses made outside of secondary care for the full set of conditions included in the study. Future studies combining primary and secondary care data may lead to more comprehensive trajectories from a greater proportion of the population. Similarly, whilst we have attempted to minimise the impact of over-counting of chronic events, we acknowledge that the lack of data on diagnoses made outside of hospital introduces uncertainty concerning the position of chronic diseases in trajectories. Nonetheless, our matched cohort design allowed for comparison of the burden of disease amongst survivors of MI over and above that of non-MI patients regardless of diagnoses made outside of secondary care.

### Conclusion

Using a systemic, data-driven, and hypothesis-free methodology, this study quantifies real-world post-MI disease trajectories among a nationwide cohort. By moving beyond cross-sectional multimorbidity analyses and quantifying the relative risks of disease pathways and their association with mortality, we have identified clear targets for public health intervention. Our study suggests that improved secondary prevention strategies, and a focus on reducing the neuro-psychiatric disease burden following MI, may minimise increased dependency and premature mortality for survivors of MI. Further understanding of the factors contributing to these trajectories is required to facilitate identification of patients in the early post-MI setting who would benefit the most from closer follow-up and prompt intervention.

## Contributors

All authors meet the criteria for authorship, with individual contributions highlighted for each criteria. MH contributed to the conception and design of the work; MH, CPG, DRW, and JW contributed to the acquisition of data and funding; CJH, MH, JAB, and JW conducted literature searching; CJH, MH, and JW contributed to the analysis and verified the underlying data; CJH constructed data visualisations; all authors contributed to interpretation of the data for the work; all authors contributed to drafting the work and revising it critically for important intellectual content; all authors approved the final version of the manuscript for publication; and all authors agreed to be accountable for all aspects of the work in ensuring that questions related to the accuracy or integrity of any part of the work are appropriately investigated and resolved.

## Data sharing statement

### Hospitalisation data:

HES data are managed and released by NHS Digital. The specific extract provided to the research team can only be used for the stated purpose of the study and for the length of time necessary to conduct the study. The extract cannot be shared outside of the research team or for any other purpose according to the legally binding terms under which they were released. Please see the privacy notice for further information on the purpose and legal basis of the use of these data: https://digital.nhs.uk/data-and-information/data-tools-and-services/data-services/hospital-episode-statistics; also available at https://multimorbidity-research-leeds.github.io/.

Access to HES data is available by direct application to NHS Digital and is available to anyone who has a legal basis for accessing these data, meets the requirements for safe and secure use of these data, and intends to use these data for demonstrable benefit to health and social care in the UK. A full HES data dictionary, information of how to apply, and the costs associated with data applications are available publicly via the NHS digital website: https://digital.nhs.uk. All diagnostic codes used to define specific study outcomes are provided in the supplementary online material released at time of publication.

### Programming code:

The programming code used to process and analyse the HES data is publicly available at https://multimorbidity-research-leeds.github.io/.

## Declaration of interests

CJH, JAB, DRW, OJ, JW, and MH have no conflicts of interest to declare. CPG has received funding from Abbott Diabetes, Bristol Myers Squibb and the European Society of Cardiology, and consulting fees from AI Nexus, AstraZeneca, Amgen, Bayer, Bristol Myers Squibb, Boehrinher-Ingleheim, CardioMatics, Chiesi, Daiichi Sankyo, GPRI Research B.V., Menarini, Novartis, iRhyth, Organon as well as payment for honoraria or lectures from AstraZeneca, Boston Scientific, Menarini, Novartis, Raisio Group, Wondr Medical, Zydus. CPG declares participation on Data Safety Monitoring or Advisory boards for the DANBLCOK and TARGET CTCA trials and editorial and committee membership of the NICE Indicator Advisory Committee, EHJ Quality of Care and Clinical Outcomes and ESC Quality Indicator Committee.
