# Supplementary material for: Disease trajectories following myocardial infarction: insights from process mining of 145 million hospitalisation episodes
Source: eBioMedicine. 2023 Sep 21;96:104792. doi: 10.1016/j.ebiom.2023.104792 (PMC10520333; doi:10.1016/j.ebiom.2023.104792)
Supplement: Supplementary Material [file mmc1.docx]

# Supplementary material

**Disease trajectories following myocardial infarction: insights from process mining of 145 million hospitalisation episodes**

**Authors:** Christopher J. Hayward (PhD),^1,2^ Jonathan A. Batty (MBChB),^1,2^ David R. Westhead (PhD),^2,3^ Owen Johnson (MSc),^4^ Chris P. Gale (PhD, FRCP),^1,2,5^ Jianhua Wu (PhD),^2,6^ Marlous Hall (PhD)^1,2^

^1^Clinical and Population Sciences Department, Leeds Institute of Cardiovascular and Metabolic Medicine, School of Medicine, Faculty of Medicine and Health, University of Leeds, Leeds, LS2 9JT, UK
^2^Leeds Institute for Data Analytics, University of Leeds, Leeds, LS2 9JT, UK
^3^School of Molecular and Cellular Biology, Faculty of Biological Sciences, University of Leeds, Leeds, LS2 9JT, UK

^4^School of Computing, Faculty of Engineering and Physical Sciences, University of Leeds, LS2 9JT, UK

^5^Department of Cardiology, Leeds Teaching Hospitals NHS Trust, Great George Street, Leeds, LS1 3EX, UK

^6^Wolfson Institute of Population Health, Queen Mary University of London, London, E1 4NS, UK

#### Section S1: Matching procedure

The risk-set matched control cohort was derived by matching any new case of myocardial infarction (MI) occurring at time *t* to any five individuals who had not yet developed MI by time t. Matching was based on an individual’s first MI episode using age, sex, month and year of episode date, and NHS trust. Where an exact matching resulted in fewer than five matched individuals per case of MI, the age criteria was relaxed by a two-year window (n=10,550; 2·6%). Study entry was either the date of the first episode with MI or first matched episode in the control cohort. All individuals were included in the risk-set matched pool, and matched individuals who later developed MI were censored at the date of MI in further analyses.

#### Section S2: Disease trajectory construction using secondary diagnoses

Our secondary analyses included both primary and secondary diagnoses in the construction of disease trajectories. There were several important considerations to consider in this analysis: Within admissions, the order of episodes is obtained using the episode order field [HES field: EPIORDER]. Each episode is associated with a single primary diagnosis [HES field: DIAG_01] – the primary analysis consists of trajectories of these primary diagnoses. Because each episode can be associated with more than one secondary diagnosis [HES field: DIAG_02 – DIAG_20] – also associated with the same timestamp [HES field: EPISTART] – temporal information on individual secondary diagnoses per episode is not explicitly present in the data. Nonetheless, in order to capture the broadest range of diagnoses possible for any given individual – representing a more complete picture of disease progression – for secondary analyses we included secondary diagnoses in trajectories as they appeared in each row of the data (as recorded by clinical coders from diagnosis field two to field 20), where each row corresponds to a single episode. For the secondary analyses, non-empty secondary diagnoses were positioned after the primary diagnosis for each episode, resulting in up to 19 secondary diagnoses following every primary diagnosis.

#### Section S3: Chronic and acute condition definitions

Chronic conditions were only included in trajectories at their first occurrence, irrespective of primary or secondary diagnosis status. Where the first occurrence of a chronic condition occurred before the index date, the condition was not present in the corresponding trajectory. Chronic conditions which first occurred after the index date were included only once in the corresponding trajectory – at their first occurrence.

Prior to trajectory construction, ICD-10 primary and secondary diagnoses codes were labelled as either acute, chronic, ‘acute and chronic’, ‘not applicable’, or ‘unknown’ using the chronic condition indicator for ICD-10-CM mapping tool (<https://hcup-us.ahrq.gov/toolssoftware/chronic_icd10/chronic_icd10.jsp>; version: v2021-1) based on the full five-character ICD-10 codes. We used a mapping algorithm which applied a ‘nearest match’ approach in cases where mappings to disease codes were not exact. In cases where a shorter version of the ICD-10 code was in the mapping, an iterative cropping process was applied to the diagnoses in an attempt to provide a match. Where several mappings existed for a shortened version of the code, an acute/chronic label was only applied where all matches indicated the same label. Of the 17,358 unique diagnosis codes across both cohorts and after applying the mapping algorithm, a mapping did not exist for 1,335 (7·7%) diagnoses, and these were marked as ‘unknown’. 2,028 (11·7%) codes had a mixture of acute and chronic labels, and were also marked as ‘unknown’. 6,323 (36·4%) were chronic; 5,330 (30·7%) were acute; 2,327 (13·4%) were defined as ‘not applicable’ to acute/chronic labelling; and 15 (0·1%) were both acute and chronic. For any codes resulting in a ‘chronic’ categorisation, these were treated as chronic and were not allowed to repeat in trajectories; codes not marked as ‘chronic’ were subsequently considered ‘acute’ and could repeat in trajectories, and were not limited to their first occurrence.

To avoid over-counting of repeat acute diagnoses within trajectories, any new acute diagnosis was counted only if it occurred a minimum of two months after a previous episode with the same diagnosis, based on the three-character ICD-10 codes.

#### Section S4: Presentation of disease trajectories

Trajectories are presented as broad disease groups based on ICD-10 chapters. This course-grained approach was necessary for the interpretability of resulting trajectories and to ensure sufficient events per trajectory for modelling of outcomes. Trajectories were generated from specific diagnoses (detailed three-character ICD-10 codes) prior to aggregation to ICD-10 chapter-level allowing for the limiting of chronic conditions to their first (earliest) occurrence, and the exclusion from trajectories of acute diagnoses that happen soon after a previous occurrence. The complete list of ICD-10 three-character codes which were considered in the construction of trajectories is presented in Table S9.

The online interactive disease trajectory tool supports our results by facilitating exploration of the specific diagnoses relating to each chapter within trajectories: <https://multimorbidity-research-leeds.github.io/>. Further detail is available via the ‘disease transition’ online application, which displays networks of the number of individuals that transition between groups of three-character ICD-10 codes: <https://multimorbidity-research-leeds.github.io/>.

**Section S5: Using the PM4Py package**

The PM4Py (Process Mining for Python) package (version 2.2.11.1) was used to speed-up the extraction of disease sequences from the data, and to support the arranging of those sequences into appropriate data structures for subsequent analyses; this tool helped to support the inferring of an underlying disease-accrual process from hospitalisation records.

1. Table S1: List of relevant Hospital Episode Statistics (HES) data columns used in the construction of disease trajectories

| Field title | Field name in HES data | Field description | Description of invalid/unknown values | Associated with |
| --- | --- | --- | --- | --- |
| HES ID | ENCRYPTED_HESID | Unique patient identifier | *Not applicable* | Patient |
| Month and year of birth | DOB | Individual’s date-of-birth (at month and year level; day data is not present) | On or before 01/01/1801 | Patient |
| Sex | SEX | Individual’s sex | 0 (not known) or 9 (not specified) | Patient |
| Admission ID | PROVSPNOPS | Admission identifier, corresponding to a single patient admission (unique within healthcare providers) | NaN (empty) | Admission |
| Discharge date | DISDATE | Discharge date corresponding to the time when the individual ends an admission (the end date of the final episode within the admission) | On or before 01/01/1801 | Admission |
| Provider code | PROCODE | Unique identifier for healthcare providers. To reduce the number of unique provider codes and enable larger groups of patients associated with a particular healthcare provider, provider codes are shortened to the first three characters. The excluded final two characters act as an identifier for the site within the organisation. | NaN (empty) | Admission |
| Deprivation index | IMD04 | A numeric score corresponding to the level of deprivation attached to the geographic area for the healthcare provider for this admission. | NaN (empty) | Admission |
| Episode start month and year | EPISTART | The episode start date (at month and year level) | On or before 01/01/1801 | Episode |
| Episode end month and year | EPIEND | The episode end date (at month and year level) | On or before 01/01/1801 | Episode |
| Survival time (days) | n/a (ONS linked) | From the corresponding episode, the number of days till patient death (only applicable if the patient died within the study period, i.e. up until March 27^th^ 2017). | *Not applicable* | Episode |
| Primary diagnosis code | DIAG_01 | ICD-10 code corresponding to the episode’s primary diagnosis | ‘R69’, ‘R69X’, ‘R69X3’, ‘R69X6’, ‘R69X8’ | Episode |
| Secondary diagnosis code(s) | DIAG_02 to DIAG_20 | Zero to nineteen ICD-10 codes corresponding to the secondary diagnoses for this episode | ‘R69’, ‘R69X’, ‘R69X3’, ‘R69X6’, ‘R69X8’ | Episode |
| Episode order | EPIORDER | A numeric value denoting the chronological order of episodes within an admission | 99 (not known) | Episode |
| Episode status | EPISTAT | A numeric value denoting whether this episode finished within this financial year (April-April); indicates duplicate episodes which were excluded. | *Not applicable* | Episode |

Invalid/unknown values are obtained from the NHS HES data dictionary, and are listed here for reference.

1. Table S2: Cleaning steps used to remove individuals for derivation of myocardial infarction (MI) and matched control cohorts

| Step | Description |
| --- | --- |
| 1 | Individuals with episodes which are not associated with an admission ID (PROVSPNOPS) are removed.  **Reason:** Episodes are ordered using the episode order field (EPIORDER) within their admission, which is determined by the admission ID; ordering of episodes is not possible without the admission ID.  **Number of excluded individuals:** MI – 1,987 (0·50%); matched controls – 16,384 (0·82%). |
| 2 | Individuals with inconsistent patient-specific data are removed (sex [SEX], date of birth [DOB]). An individual was deemed as having consistent sex/DOB where more than 75% of the episodes for that individual had matching values. **Reason:** Downstream models use sex and age as covariates, and rely on confidently knowing sex and age for individuals. **Number of excluded individuals:** MI – 394 (0·10%); matched controls – 1,934 (0·10%). |
| 3 | Individuals with admissions that contain duplicate episode order values (EPIORDER) are removed. **Reason:** Without episode order, episodes cannot be ordered with confidence within admissions. **Number of excluded individuals:** MI – 924 (0·23%); matched controls – 6,628 (0·33%). |
| 4 | As a final step in the cleaning procedure, all controls which were matched with one of the cases from the MI cohort removed during cleaning steps were also removed, and vice versa.  **Reason:** This preserves the original 5:1 ratio: five matched controls for each individual with MI.  **Number of excluded individuals:** MI – 20,135 (5·05%); matched controls – 92,254 (4·62%). |

1. Table S3: Steps for selecting diagnoses for inclusion in disease trajectories

| Step | Description |
| --- | --- |
| 1 | Exclude episodes with unknown episode start/end date (EPISTART/EPIEND).  **Number of excluded episodes:** MI – 11 (0·00041%); matched controls – 69 (0·00042%) |
| 2 | Exclude episodes with unknown or invalid diagnosis (DIAG_01). When building trajectories using both primary and secondary diagnoses, then DIAG_02 to DIAG_20 are also considered.  **Number of excluded episodes:** MI – 15,136 (0·57%); matched controls – 122,521 (0·75%) |
| 3 | Exclude episodes with episode start date (EPISTART) occurring before an individual’s matched (index) date (exclude those before the start of the trajectory).  **Number of excluded episodes:** MI – 358,503 (13·40%); matched controls – 3,636,559 (22·25%) |
| 4 | For risk-set matched controls with an MI episode, exclude episodes with an episode start date (EPISTART) on or after the date of the first MI episode.  **Number of excluded episodes:** MI – 0 (0·0%); matched controls – 110,330 (0·67%) |
| 5 | Separately for the MI and control cohorts: Exclude episodes with rare diagnoses (DIAG_01 – DIAG_20) i.e. diagnoses which appear in less than 0·1% of individuals. Occurrences of diagnoses in both primary and secondary fields are used when determining rarity.  **Number of excluded episodes:** MI – 41,037 (1·53%); matched controls – 267,183 (1·63%). |
| At this stage, admissions and episodes within admissions are assigned a fixed chronological order, using admission dates, episode dates, and episode survival time^*^. Episodes within admissions are ordered using the episode-order field (EPIORDER). Where date anonymisation has meant that the true order cannot be determined (i.e. where more than one admission occurs in the same month), a fixed random order is chosen for admissions within their respective month. When building trajectories using both primary and secondary diagnoses, secondary diagnoses are included in the order that they appear within each episode after the primary diagnosis, i.e. from DIAG_01 to DIAG_20. | |
| 6 | At any point (before, on, or after the index date), for diagnoses marked as chronic, include only its first occurrence and exclude subsequent occurrences. Acute diagnoses are unaffected.  **Number of excluded episodes:** MI – 772,723 (28·88%); matched controls – 4,362,756 (26·69%) |
| 7 | Exclude diagnoses occurring within two months from the most recent occurrence (using episode start and end dates: EPISTART and EPIEND). i.e. episodes with the same diagnosis which happen close together are regarded as a single instance of that diagnosis, appearing as a single step in the trajectory.  **Number of excluded episodes:** MI – 328,940 (12·30%); matched controls – 2,165,239 (13·25%) |
| 8 | Exclude non-disease diagnoses, i.e. those outside of the range A00-N99 (e.g. related to: injury, pregnancy).  **Number of excluded episodes:** MI – 261,539 (9·77%); matched controls – 1,619,184 (9·90%) |
| 9 | Assign the ‘initial episode’ for each individual (the starting ‘MI/Initial’ event in trajectories). For individuals with MI, this is the episode containing the first instance of MI, in a primary or secondary diagnosis field. For controls, an episode on the index date (at month level) is chosen at random (an episode from any admission within that month). Episodes which occur before the selected initial episode (based on the fixed assigned ordering) – but existing in the same month due to the resolution of the timestamps – are excluded.  **Caused by including the initial episode in trajectories, number of episodes un-excluded:** MI – 17,819 (0·58%); matched controls – 804,255 (4·41%)  **Excluded episodes occurring before this selected initial episode:** MI – 43,308 (1·42%); matched controls: – 154,648 (0·85%)  **Net change:** MI – 25,489 (0·84%) more exclusions; matched controls: – 649,607 (3·56%) fewer exclusions |

* Survival time is measured in the number of days till death, linked to each episode. Using survival time helps to identify the true order of admissions when admissions – recorded at month-level granularity – are tied.

**Table S4: REporting of studies Conducted using Observational Routinely-collected Data (RECORD) Standard**

**[Extension of the existing STROBE guidelines (STrengthening the Reporting of OBservational studies in Epidemiology)]**

|  | **Item No.** | **STROBE items** | **RECORD items** | **Location in manuscript where items are reported** |
| --- | --- | --- | --- | --- |
| Title and Abstract | | | | |
|  | 1 | (a) Indicate the study’s design with a commonly used term in the title or the abstract (b) Provide in the abstract an informative and balanced summary of what was done and what was found | RECORD 1.1: The type of data used should be specified in the title or abstract. When possible, the name of the databases used should be included.  RECORD 1.2: If applicable, the geographic region and timeframe within which the study took place should be reported in the title or abstract.  RECORD 1.3: If linkage between databases was conducted for the study, this should be clearly stated in the title or abstract. | Abstract |
| Introduction | | | | |
| Background rationale | 2 | Explain the scientific background and rationale for the investigation being reported |  | Research in Context;  Introduction – Paragraph 1, 2, 3 |
| Objectives | 3 | State specific objectives, including any prespecified hypotheses |  | Introduction – Paragraph 1, 2 |
| Methods | | | | |
| Study Design | 4 | Present key elements of study design early in the paper |  | Methods – Paragraph 1 and *Data*; |
| Setting | 5 | Describe the setting, locations, and relevant dates, including periods of recruitment, exposure, follow-up, and data collection |  | Methods – Paragraph 1 and *Data*; |
| Participants | 6 | *(a) Cohort study* - Give the eligibility criteria, and the sources and methods of selection of participants. Describe methods of follow-up  *Case-control study* - Give the eligibility criteria, and the sources and methods of case ascertainment and control selection. Give the rationale for the choice of cases and controls  *Cross-sectional study* - Give the eligibility criteria, and the sources and methods of selection of participants  *(b) Cohort study* - For matched studies, give matching criteria and number of exposed and unexposed  *Case-control study* - For matched studies, give matching criteria and the number of controls per case | RECORD 6.1: The methods of study population selection (such as codes or algorithms used to identify subjects) should be listed in detail. If this is not possible, an explanation should be provided.  RECORD 6.2: Any validation studies of the codes or algorithms used to select the population should be referenced. If validation was conducted for this study and not published elsewhere, detailed methods and results should be provided.  RECORD 6.3: If the study involved linkage of databases, consider use of a flow diagram or other graphical display to demonstrate the data linkage process, including the number of individuals with linked data at each stage. | Methods (cohort definition paragraph)  Figure 1 gives detailed cohort definition as flow chart;  Code lists are provided in the supplementary material;  Matching method explained in supplementary material – Section S1 |
| Variables | 7 | Clearly define all outcomes, exposures, predictors, potential confounders, and effect modifiers. Give diagnostic criteria, if applicable. | RECORD 7.1: A complete list of codes and algorithms used to classify exposures, outcomes, confounders, and effect modifiers should be provided. If these cannot be reported, an explanation should be provided. | List of confounding variables is provided in Methods;  Code lists are provided in the supplementary material;  Method of incorporating secondary diagnoses in explained in the supplementary material (Section S2);  Acute/chronic mapping algorithm is explained in the supplementary material (Section S3) |
| Data sources/ measurement | 8 | For each variable of interest, give sources of data and details of methods of assessment (measurement).  Describe comparability of assessment methods if there is more than one group |  | Details of data source provided in methods, *Data*;  Table S1 provides list of variables of interest |
| Bias | 9 | Describe any efforts to address potential sources of bias |  | Methods detail adjustment for confounders;  Risk-set matching procedure – Cohort definition and Section S1 |
| Study size | 10 | Explain how the study size was arrived at |  | Definition of exposed cohort provided in cohort definition paragraph;  Exclusion criteria provided in methods, and in the supplementary material (Table S2);  Figure 1 gives detailed cohort definition |
| Quantitative variables | 11 | Explain how quantitative variables were handled in the analyses. If applicable, describe which groupings were chosen, and why |  | Handling of variables described in Statistical Analyses section (Methods) |
| Statistical methods | 12 | (a) Describe all statistical methods, including those used to control for confounding  (b) Describe any methods used to examine subgroups and interactions  (c) Explain how missing data were addressed  (d) *Cohort study* - If applicable, explain how loss to follow-up was addressed  *Case-control study* - If applicable, explain how matching of cases and controls was addressed  *Cross-sectional study* - If applicable, describe analytical methods taking account of sampling strategy  (e) Describe any sensitivity analyses |  | Statistical Analyses section (Methods): Description of statistical methods, including sensitivity analyses, landmark analyses, model design, and approach of sub-group analyses. |
| Data access and cleaning methods |  | .. | RECORD 12.1: Authors should describe the extent to which the investigators had access to the database population used to create the study population.  RECORD 12.2: Authors should provide information on the data cleaning methods used in the study. | Description of access to data provided in Methods;  Data cleaning steps provided in supplementary material – Table S2 |
| Linkage |  | .. | RECORD 12.3: State whether the study included person-level, institutional-level, or other data linkage across two or more databases. The methods of linkage and methods of linkage quality evaluation should be provided. | Description of linkage provided in Methods |
| Results | | | | |
| Participants | 13 | (a) Report the numbers of individuals at each stage of the study (*e.g.*, numbers potentially eligible, examined for eligibility, confirmed eligible, included in the study, completing follow-up, and analysed)  (b) Give reasons for non-participation at each stage.  (c) Consider use of a flow diagram | RECORD 13.1: Describe in detail the selection of the persons included in the study (*i.e.,* study population selection) including filtering based on data quality, data availability and linkage. The selection of included persons can be described in the text and/or by means of the study flow diagram. | Definition of exposed cohort provided in cohort definition paragraph;  Exclusion criteria provided in methods, and in the supplementary material (Table S2 and S3);  Figure 1 gives detailed cohort definition |
| Descriptive data | 14 | (a) Give characteristics of study participants (*e.g.*, demographic, clinical, social) and information on exposures and potential confounders  (b) Indicate the number of participants with missing data for each variable of interest  (c) *Cohort study* - summarise follow-up time (*e.g.*, average and total amount) |  | Patient characteristics, missing data counts, follow-up time, provided in Table 1 |
| Outcome data | 15 | *Cohort study* - Report numbers of outcome events or summary measures over time  *Case-control study* - Report numbers in each exposure category, or summary measures of exposure  *Cross-sectional study* - Report numbers of outcome events or summary measures |  | Results section;  Table 1  Figures 2, 3, and 4 |
| Main results | 16 | (a) Give unadjusted estimates and, if applicable, confounder-adjusted estimates and their precision (e.g., 95% confidence interval). Make clear which confounders were adjusted for and why they were included  (b) Report category boundaries when continuous variables were categorized  (c) If relevant, consider translating estimates of relative risk into absolute risk for a meaningful time period |  | Results section, describing relevant statistics including relative risks and hazard ratios;  Methods section details adjustment for confounders |
| Other analyses | 17 | Report other analyses done—e.g., analyses of subgroups and interactions, and sensitivity analyses |  | Description of sensitivity analyses (landmark, and ordering of trajectories), and subgroup analyses (sex) presented in Results |
| Discussion | | | | |
| Key results | 18 | Summarise key results with reference to study objectives |  | Discussion section, first paragraph |
| Limitations | 19 | Discuss limitations of the study, taking into account sources of potential bias or imprecision. Discuss both direction and magnitude of any potential bias | RECORD 19.1: Discuss the implications of using data that were not created or collected to answer the specific research question(s). Include discussion of misclassification bias, unmeasured confounding, missing data, and changing eligibility over time, as they pertain to the study being reported. | Discussion, final paragraphs detail limitations |
| Interpretation | 20 | Give a cautious overall interpretation of results considering objectives, limitations, multiplicity of analyses, results from similar studies, and other relevant evidence |  | Clinical interpretation presented in Discussion, including conclusion;  Overall interpretation described in ‘Research in Context’. |
| Generalisability | 21 | Discuss the generalisability (external validity) of the study results |  | Discussion section and Conclusion |
| Other Information | | | | |
| Funding | 22 | Give the source of funding and the role of the funders for the present study and, if applicable, for the original study on which the present article is based |  | End of methods section;  Acknowledgements. |
| Accessibility of protocol, raw data, and programming code |  | .. | RECORD 22.1: Authors should provide information on how to access any supplemental information such as the study protocol, raw data, or programming code. | Link to study protocol and programming code provided on group website: [*https://multimorbidity-research-leeds.github.io/*](https://multimorbidity-research-leeds.github.io/)*.* |

**Table S5: CODE-EHR framework: best practice checklist to report on the use of structured electronic healthcare records in clinical research**

| **Item** | **Objective** | **Framework standards** | **Minimum information to provide** | **Lead author acknowledgment** |
| --- | --- | --- | --- | --- |
| 1. Dataset construction and linkage | To provide an understanding of how the structured healthcare data were identified and used. | Minimum: Flow diagram of datasets used in the study, and description of the processes and directionality of any linkage performed, published within the research report or supplementary documents. Preferred: Provided within a pre-published protocol or open access document. | (a) State the source of any datasets used. (b) Comment on how the observed and any missing data were identified and addressed, and the proportion observed for each variable. (c) Provide data on completeness of follow-up. (d) For linked datasets, specify how linkage was performed and the quality of linkage methods. | Choose one from: (1) Minimum standard not met  (2) Minimum standard met OR (3) Preferred standard met |
| 2. Data fit for purpose | To ensure transparency with the approach taken, with respect to coding of the structured healthcare data. | Minimum: Clear unambiguous statements on the process of coding in the methods section of the research report. Preferred: Provided within a pre-published protocol or open access document. | (a) Confirm origin, clinical processes, and the purpose of data. (b) Specify coding systems, clinical terminologies, or classification used and their versions, and any manipulation of the coded data. (c) Provide detail on quality assessment for data capture. (d) Outline potential sources of bias. | Choose one from: (1) Minimum standard not met  (2) Minimum standard met OR (3) Preferred standard met |
| 3. Disease and outcome definitions | To fully detail how conditions AND outcome events were defined, allowing other researchers to identify errors and repeat the process in other datasets. | Minimum: State what codes were used to define diseases, treatments, conditions, and outcomes *prior to statistical analysis*, including those relating to patient identification, therapy, procedures, comorbidities, and components of any composite endpoints. Preferred: Provided within a pre-published protocol or open access document *prior to statistical analysis*. | (a) Detailed lists of codes used for each aspect of the study. (b) Date of publication and access details for the coding manual (please add to box below). (c) Provide definitions, implementation logic and validation of any phenotyping algorithms used. (d) Specify any processes used to validate the coding scheme or reference to prior work. | Choose one from: (1) Minimum standard not met  (2) Minimum standard met OR (3) Preferred standard met |
| 4. Analysis | To fully detail how outcome events were analysed and allow independent assessment of the authenticity of study findings. | Minimum: Describe the process used to analyse study outcomes, including statistical methods and use of any machine learning or algorithmic approaches. Preferred: Provide a statistical analysis plan as a supplementary file, locked before analyses commencing. | (a) Provide details on all statistical methods used. (b) Provide links to any machine code or algorithms used in the analysis, preferably as open source. (c) Specify the processes of testing assumptions, assessing model fit and any internal validation. (d) Specify how generalisability of results was assessed, the replication of findings in other datasets, or any external validation. | Choose one from: (1) Minimum standard not met  (2) Minimum standard met OR (3) Preferred standard met |
| 5. Ethics and governance | To provide patients, who might or might not have given consent, and regulatory authorities the ability to interrogate the security and provenance of the data. | Minimum: Clear unambiguous statements on how the principles of Good Clinical Practice and Data Protection will be/were met, provided in the methods section of the research report. Preferred: Provided within a pre-published protocol or open access document with evidence of patient and public engagement. | (a) State how informed consent was acquired, or governance if no patient consent. (b) Specify how data privacy was protected in the collection and storage of data. (c) Detail what steps were taken for patient and public involvement in the research study. (d) Provide information on where anonymised source data or code can be obtained for verification and further research. | Choose one from: (1) Minimum standard not met  (2) Minimum standard met OR (3) Preferred standard met |
| 6. Coding manual | DOI of publication or website address: *Included in manuscript online supplement - https://multimorbidity-research-leeds.github.io/.* Date published: 09/2023 | | | |
| 7. Comments |  | | | |
| **8. Summary declaration** | Choose one from: One or more minimum standards not met OR **All minimum standards met** | | | |

1. Table S6: The number of individuals following trajectories of a given length, per cohort, per analysis, and the corresponding average follow-up duration

|  | **For trajectories including primary diagnoses only (primary analysis)** | | | | **For trajectories including primary and secondary diagnoses (secondary analysis)** | | | |
| --- | --- | --- | --- | --- | --- | --- | --- | --- |
|  | **MI Cohort** | | **Matched Controls Cohort** | | **MI Cohort** | | **Matched Controls Cohort** | |
| Number of diagnoses post-MI/Initial | Number of individuals | Follow-up duration (years), median [Q1,Q3] | Number of individuals | Follow-up duration (years), median [Q1,Q3] | Number of individuals | Follow-up duration (years), median [Q1,Q3] | Number of individuals | Follow-up duration (years), median [Q1,Q3] |
| 0 | 170,294 (45·3%) | 2·1 [0·4,4·5] | 773,284 (41·2%) | 2·6 [1·1,4·8] | 11,284 (3·0%) | 2·7 [0·2,6·0] | 415,153 (22·1%) | 2·7 [1·1,5·0] |
| 1 | 87,527 (23·3%) | 3·9 [1·9,6·1] | 442,870 (23·6%) | 3·7 [1·7,5·9] | 24,439 (6·5%) | 2·9 [0·8,5·6] | 227,536 (12·1%) | 3·1 [1·4,5·4] |
| 2 | 48,678 (13·0%) | 4·6 [2·6,6·7] | 262,622 (14·0%) | 4·3 [2·3,6·5] | 34,591 (9·2%) | 2·8 [1·0,5·3] | 177,648 (9·5%) | 3·4 [1·6,5·7] |
| 3 | 28,442 (7·6%) | 5·1 [3·1,7·0] | 155,866 (8·3%) | 4·9 [2·8,6·9] | 38,071 (10·1%) | 3·0 [1·1,5·3] | 145,161 (7·7%) | 3·7 [1·7,6·0] |
| 4 | 16,393 (4·4%) | 5·4 [3·5,7·2] | 93,371 (5·0%) | 5·3 [3·3,7·1] | 36,162 (9·6%) | 3·1 [1·1,5·5] | 121,562 (6·5%) | 3·8 [1·7,6·1] |
| 5 | 9,587 (2·6%) | 5·7 [3·9,7·3] | 55,955 (3·0%) | 5·7 [3·8,7·3] | 32,013 (8·5%) | 3·2 [1·1,5·7] | 102,984 (5·5%) | 4·0 [1·8,6·2] |
| 6 | 5,805 (1·5%) | 5·9 [4·1,7·4] | 33,957 (1·8%) | 5·9 [4·1,7·5] | 27,305 (7·3%) | 3·4 [1·3,5·9] | 88,238 (4·7%) | 4·1 [1·8,6·3] |
| 7 | 3,452 (0·9%) | 6·1 [4·5,7·6] | 21,477 (1·1%) | 6·2 [4·5,7·7] | 22,911 (6·1%) | 3·6 [1·4,6·0] | 75,766 (4·0%) | 4·1 [1·9,6·4] |
| 8 | 2,022 (0·5%) | 6·3 [4·7,7·7] | 13,595 (0·7%) | 6·5 [4·8,7·8] | 19,846 (5·3%) | 3·7 [1·4,6·1] | 65,702 (3·5%) | 4·2 [2·0,6·5] |
| 9 | 1,231 (0·3%) | 6·6 [5·0,7·8] | 8,463 (0·5%) | 6·7 [5·0,7·9] | 16,751 (4·5%) | 3·8 [1·6,6·1] | 56,957 (3·0%) | 4·2 [2·0,6·5] |
| 10 | 792 (0·2%) | 6·6 [5·2,7·7] | 5,486 (0·3%) | 6·9 [5·3,8·0] | 14,382 (3·8%) | 3·8 [1·6,6·2] | 49,144 (2·6%) | 4·2 [2·1,6·6] |
| 11 | 483 (0·1%) | 6·8 [5·2,7·9] | 3,490 (0·2%) | 7·0 [5·4,8·1] | 12,418 (3·3%) | 3·9 [1·7,6·2] | 43,390 (2·3%) | 4·4 [2·2,6·7] |
| 12 | 318 (0·1%) | 6·8 [5·5,8·0] | 2,413 (0·1%) | 7·1 [5·7,8·1] | 10,320 (2·7%) | 3·9 [1·7,6·3] | 37,515 (2·0%) | 4·4 [2·3,6·7] |
| 13 | 201 (0·1%) | 7·0 [5·9,8·1] | 1,676 (0·1%) | 7·2 [6·0,8·1] | 9,262 (2·5%) | 4·1 [1·9,6·4] | 32,895 (1·8%) | 4·5 [2·4,6·7] |
| 14 | 136 (< 0·05%) | 7·2 [5·6,8·1] | 1,121 (0·1%) | 7·3 [6·1,8·2] | 8,012 (2·1%) | 4·1 [1·9,6·3] | 28,998 (1·5%) | 4·5 [2·4,6·7] |
| 15 | 102 (< 0·05%) | 6·9 [5·6,8·0] | 821 (< 0·05%) | 7·4 [6·2,8·2] | 6,976 (1·9%) | 4·2 [2·1,6·4] | 24,912 (1·3%) | 4·7 [2·6,6·8] |
| 16 | 73 (< 0·05%) | 7·7 [6·4,8·1] | 531 (< 0·05%) | 7·6 [6·5,8·4] | 6,100 (1·6%) | 4·2 [2·1,6·4] | 21,945 (1·2%) | 4·7 [2·7,6·8] |
| 17 | 48 (< 0·05%) | 7·7 [6·7,8·6] | 386 (< 0·05%) | 7·7 [6·5,8·3] | 5,374 (1·4%) | 4·3 [2·2,6·5] | 19,284 (1·0%) | 4·8 [2·8,6·8] |
| 18 | 23 (< 0·05%) | 7·5 [6·8,7·9] | 257 (< 0·05%) | 7·7 [6·8,8·4] | 4,640 (1·2%) | 4·3 [2·3,6·4] | 16,614 (0·9%) | 4·8 [2·9,6·9] |
| 19 | 22 (< 0·05%) | 7·4 [6·6,8·4] | 193 (< 0·05%) | 7·8 [6·8,8·5] | 4,160 (1·1%) | 4·3 [2·4,6·4] | 14,859 (0·8%) | 5·0 [3·0,6·9] |
| 20 | 11 (< 0·05%) | 7·1 [5·8,7·9] | 144 (< 0·05%) | 7·9 [7·0,8·4] | 3,577 (1·0%) | 4·5 [2·6,6·6] | 12,956 (0·7%) | 5·0 [3·2,7·0] |
| 21 | 11 (< 0·05%) | 7·1 [6·2,8·6] | 101 (< 0·05%) | 7·7 [6·9,8·3] | 3,209 (0·9%) | 4·6 [2·5,6·7] | 11,342 (0·6%) | 5·1 [3·2,7·0] |
| 22 | < 10 (< 0·05%) | 7·9 [7·5,8·0] | 80 (< 0·05%) | 8·1 [7·5,8·6] | 2,802 (0·7%) | 4·5 [2·7,6·6] | 10,070 (0·5%) | 5·2 [3·3,7·1] |
| 23 | < 10 (< 0·05%) | 7·5 [6·9,8·7] | 42 (< 0·05%) | 8·3 [7·5,8·6] | 2,518 (0·7%) | 4·8 [2·8,6·8] | 8,775 (0·5%) | 5·2 [3·3,7·1] |
| 24 | < 10 (< 0·05%) | 7·2 [7·2,7·2] | 39 (< 0·05%) | 8·3 [7·6,8·7] | 2,145 (0·6%) | 4·8 [2·9,6·8] | 7,717 (0·4%) | 5·3 [3·5,7·1] |
| 25 | 0 (0%) | n/a | 21 (< 0·05%) | 8·1 [7·7,8·7] | 1,884 (0·5%) | 4·8 [3·0,6·7] | 6,801 (0·4%) | 5·4 [3·7,7·2] |
| 26 | < 10 (< 0·05%) | 7·2 [7·2,7·2] | 22 (< 0·05%) | 7·9 [7·0,8·6] | 1,771 (0·5%) | 4·8 [2·9,6·7] | 6,010 (0·3%) | 5·5 [3·7,7·2] |
| 27 | < 10 (< 0·05%) | 7·6 [7·6,7·6] | 12 (< 0·05%) | 8·5 [8·0,8·7] | 1,469 (0·4%) | 4·9 [3·1,6·8] | 5,293 (0·3%) | 5·4 [3·7,7·1] |
| 28 | 0 (0%) | n/a | < 10 (< 0·05%) | 8·1 [7·4,8·8] | 1,287 (0·3%) | 5·1 [3·2,6·7] | 4,641 (0·2%) | 5·6 [3·8,7·2] |
| 29 | < 10 (< 0·05%) | 6·7 [6·1,7·3] | < 10 (< 0·05%) | 8·2 [8·1,8·3] | 1,156 (0·3%) | 4·9 [3·2,6·6] | 4,116 (0·2%) | 5·6 [3·8,7·3] |
| 30 | 0 (0%) | n/a | < 10 (< 0·05%) | 8·1 [7·6,8·3] | 1,036 (0·3%) | 5·2 [3·3,7·0] | 3,627 (0·2%) | 5·7 [3·9,7·2] |
| 31 | 0 (0%) | n/a | < 10 (< 0·05%) | 8·7 [7·5,8·9] | 881 (0·2%) | 5·3 [3·6,7·0] | 3,235 (0·2%) | 5·8 [4·1,7·3] |
| 32 | 0 (0%) | n/a | < 10 (< 0·05%) | 8·5 [7·8,8·8] | 792 (0·2%) | 5·2 [3·4,7·0] | 2,886 (0·2%) | 5·7 [4·1,7·3] |
| 33 | 0 (0%) | n/a | < 10 (< 0·05%) | 9·0 [8·9,9·0] | 710 (0·2%) | 5·3 [3·7,6·9] | 2,532 (0·1%) | 5·8 [4·2,7·4] |
| 34 | 0 (0%) | n/a | < 10 (< 0·05%) | 8·8 [8·8,8·8] | 582 (0·2%) | 5·3 [3·7,7·1] | 2,321 (0·1%) | 6·0 [4·1,7·5] |
| 35 | 0 (0%) | n/a | < 10 (< 0·05%) | 7·4 [7·3,8·2] | 548 (0·1%) | 5·3 [3·7,6·9] | 2,087 (0·1%) | 5·9 [4·3,7·5] |
| 36 | 0 (0%) | n/a | < 10 (< 0·05%) | 8·0 [8·0,8·0] | 496 (0·1%) | 5·5 [3·9,7·2] | 1,791 (0·1%) | 5·8 [4·3,7·4] |
| 37 | 0 (0%) | n/a | < 10 (< 0·05%) | 8·3 [8·2,8·4] | 407 (0·1%) | 5·5 [4·0,7·4] | 1,652 (0·1%) | 5·9 [4·4,7·5] |
| 38 | 0 (0%) | n/a | < 10 (< 0·05%) | 8·9 [8·9,8·9] | 388 (0·1%) | 5·7 [3·8,7·4] | 1,473 (0·1%) | 6·1 [4·5,7·6] |
| 39 | 0 (0%) | n/a | < 10 (< 0·05%) | 8·7 [8·7,8·7] | 293 (0·1%) | 5·7 [4·0,7·2] | 1,274 (0·1%) | 6·2 [4·5,7·7] |
| 40 | 0 (0%) | n/a | < 10 (< 0·05%) | 8·7 [8·7,8·7] | 289 (0·1%) | 5·6 [4·1,7·1] | 1,138 (0·1%) | 6·3 [4·6,7·6] |
| 41 | 0 (0%) | n/a | 0 (0%) | n/a | 270 (0·1%) | 5·8 [4·4,7·4] | 1,009 (0·1%) | 6·3 [4·7,7·8] |
| 42 | 0 (0%) | n/a | 0 (0%) | n/a | 226 (0·1%) | 5·5 [4·0,7·1] | 888 (< 0·05%) | 6·3 [4·7,7·7] |
| 43 | 0 (0%) | n/a | 0 (0%) | n/a | 195 (0·1%) | 6·1 [4·4,7·5] | 860 (< 0·05%) | 6·4 [4·6,7·7] |
| 44 | 0 (0%) | n/a | 0 (0%) | n/a | 175 (< 0·05%) | 6·2 [4·8,7·5] | 673 (< 0·05%) | 6·3 [4·7,7·7] |
| 45 | 0 (0%) | n/a | 0 (0%) | n/a | 162 (< 0·05%) | 5·8 [4·2,7·4] | 619 (< 0·05%) | 6·4 [4·8,7·7] |
| 46 | 0 (0%) | n/a | 0 (0%) | n/a | 117 (< 0·05%) | 6·2 [4·7,7·5] | 629 (< 0·05%) | 6·3 [4·8,7·8] |
| 47 | 0 (0%) | n/a | 0 (0%) | n/a | 143 (< 0·05%) | 5·8 [4·4,7·4] | 527 (< 0·05%) | 6·3 [4·8,7·6] |
| 48 | 0 (0%) | n/a | 0 (0%) | n/a | 102 (< 0·05%) | 6·3 [4·0,7·5] | 534 (< 0·05%) | 6·4 [4·7,7·7] |
| 49 | 0 (0%) | n/a | 0 (0%) | n/a | 106 (< 0·05%) | 6·0 [4·5,7·2] | 430 (< 0·05%) | 6·3 [4·9,7·6] |
| 50 | 0 (0%) | n/a | 0 (0%) | n/a | 85 (< 0·05%) | 6·2 [4·6,7·3] | 433 (< 0·05%) | 6·5 [5·0,7·9] |
| 51 | 0 (0%) | n/a | 0 (0%) | n/a | 82 (< 0·05%) | 5·9 [4·8,7·1] | 349 (< 0·05%) | 6·7 [5·2,7·9] |
| 52 | 0 (0%) | n/a | 0 (0%) | n/a | 72 (< 0·05%) | 5·7 [4·4,7·3] | 339 (< 0·05%) | 6·7 [5·3,8·0] |
| 53 | 0 (0%) | n/a | 0 (0%) | n/a | 79 (< 0·05%) | 6·0 [4·8,7·5] | 275 (< 0·05%) | 6·7 [5·0,7·7] |
| 54 | 0 (0%) | n/a | 0 (0%) | n/a | 58 (< 0·05%) | 6·0 [4·7,7·1] | 262 (< 0·05%) | 6·6 [5·2,8·1] |
| 55 | 0 (0%) | n/a | 0 (0%) | n/a | 58 (< 0·05%) | 6·6 [5·5,7·8] | 239 (< 0·05%) | 6·7 [5·1,7·9] |
| 56 | 0 (0%) | n/a | 0 (0%) | n/a | 39 (< 0·05%) | 6·5 [4·9,7·2] | 190 (< 0·05%) | 6·3 [5·3,7·6] |
| 57 | 0 (0%) | n/a | 0 (0%) | n/a | 53 (< 0·05%) | 6·3 [5·0,7·9] | 177 (< 0·05%) | 6·7 [5·2,7·8] |
| 58 | 0 (0%) | n/a | 0 (0%) | n/a | 39 (< 0·05%) | 6·0 [4·7,7·0] | 158 (< 0·05%) | 7·0 [5·4,7·9] |
| 59 | 0 (0%) | n/a | 0 (0%) | n/a | 41 (< 0·05%) | 7·0 [5·3,7·8] | 151 (< 0·05%) | 7·2 [5·9,8·1] |
| 60 | 0 (0%) | n/a | 0 (0%) | n/a | 30 (< 0·05%) | 6·7 [5·4,8·0] | 131 (< 0·05%) | 6·8 [5·7,8·1] |
| 61 | 0 (0%) | n/a | 0 (0%) | n/a | 19 (< 0·05%) | 6·3 [5·6,7·6] | 149 (< 0·05%) | 6·8 [5·3,8·0] |
| 62 | 0 (0%) | n/a | 0 (0%) | n/a | 23 (< 0·05%) | 5·8 [5·0,7·2] | 108 (< 0·05%) | 7·1 [5·8,8·0] |
| 63 | 0 (0%) | n/a | 0 (0%) | n/a | 18 (< 0·05%) | 6·8 [6·2,7·6] | 111 (< 0·05%) | 6·8 [5·7,7·7] |
| 64 | 0 (0%) | n/a | 0 (0%) | n/a | 19 (< 0·05%) | 6·3 [5·4,7·1] | 95 (< 0·05%) | 7·1 [5·6,8·2] |
| 65 | 0 (0%) | n/a | 0 (0%) | n/a | 22 (< 0·05%) | 7·0 [5·8,7·4] | 99 (< 0·05%) | 7·2 [5·8,8·1] |
| 66 | 0 (0%) | n/a | 0 (0%) | n/a | 11 (< 0·05%) | 6·8 [5·9,8·0] | 81 (< 0·05%) | 6·8 [5·8,8·1] |
| 67 | 0 (0%) | n/a | 0 (0%) | n/a | 16 (< 0·05%) | 7·3 [5·7,7·8] | 49 (< 0·05%) | 6·8 [5·8,7·9] |
| 68 | 0 (0%) | n/a | 0 (0%) | n/a | 19 (< 0·05%) | 7·0 [6·0,7·7] | 62 (< 0·05%) | 7·4 [6·0,8·2] |
| 69 | 0 (0%) | n/a | 0 (0%) | n/a | 12 (< 0·05%) | 6·8 [5·8,7·4] | 68 (< 0·05%) | 7·6 [6·3,8·3] |
| 70 | 0 (0%) | n/a | 0 (0%) | n/a | 10 (< 0·05%) | 6·4 [5·1,8·5] | 48 (< 0·05%) | 7·0 [6·3,8·3] |
| 71 | 0 (0%) | n/a | 0 (0%) | n/a | 17 (< 0·05%) | 7·1 [6·2,7·5] | 54 (< 0·05%) | 7·4 [6·3,8·2] |
| 72 | 0 (0%) | n/a | 0 (0%) | n/a | < 10 (< 0·05%) | 5·9 [5·9,6·7] | 44 (< 0·05%) | 7·4 [6·5,7·8] |
| 73 | 0 (0%) | n/a | 0 (0%) | n/a | < 10 (< 0·05%) | 6·4 [5·6,6·9] | 44 (< 0·05%) | 7·1 [6·0,8·0] |
| 74 | 0 (0%) | n/a | 0 (0%) | n/a | < 10 (< 0·05%) | 5·8 [5·3,7·2] | 39 (< 0·05%) | 7·1 [5·5,8·1] |
| 75 | 0 (0%) | n/a | 0 (0%) | n/a | < 10 (< 0·05%) | 7·7 [7·2,8·0] | 37 (< 0·05%) | 7·2 [6·0,7·7] |
| 76 | 0 (0%) | n/a | 0 (0%) | n/a | < 10 (< 0·05%) | 6·2 [5·7,6·3] | 35 (< 0·05%) | 7·7 [6·0,8·4] |
| 77 | 0 (0%) | n/a | 0 (0%) | n/a | < 10 (< 0·05%) | 7·4 [6·9,7·6] | 24 (< 0·05%) | 7·9 [6·6,8·4] |
| 78 | 0 (0%) | n/a | 0 (0%) | n/a | < 10 (< 0·05%) | 6·7 [5·9,7·9] | 19 (< 0·05%) | 7·6 [6·7,8·5] |
| 79 | 0 (0%) | n/a | 0 (0%) | n/a | < 10 (< 0·05%) | 6·8 [5·9,7·8] | 31 (< 0·05%) | 7·4 [5·9,8·0] |
| 80 | 0 (0%) | n/a | 0 (0%) | n/a | < 10 (< 0·05%) | 5·8 [5·8,7·4] | 20 (< 0·05%) | 6·7 [6·1,7·9] |
| 81 | 0 (0%) | n/a | 0 (0%) | n/a | < 10 (< 0·05%) | 6·1 [5·6,7·2] | 17 (< 0·05%) | 7·5 [6·5,8·1] |
| 82 | 0 (0%) | n/a | 0 (0%) | n/a | < 10 (< 0·05%) | 7·4 [6·5,8·1] | 19 (< 0·05%) | 7·3 [6·3,8·1] |
| 83 | 0 (0%) | n/a | 0 (0%) | n/a | < 10 (< 0·05%) | 5·9 [5·9,5·9] | 11 (< 0·05%) | 7·9 [7·3,8·3] |
| 84 | 0 (0%) | n/a | 0 (0%) | n/a | < 10 (< 0·05%) | 7·7 [7·5,7·8] | 13 (< 0·05%) | 8·2 [7·3,8·5] |
| 85 | 0 (0%) | n/a | 0 (0%) | n/a | < 10 (< 0·05%) | 7·2 [7·2,7·8] | 18 (< 0·05%) | 7·4 [6·2,8·3] |
| 86 | 0 (0%) | n/a | 0 (0%) | n/a | < 10 (< 0·05%) | 6·5 [6·5,6·5] | < 10 (< 0·05%) | 7·1 [6·0,7·9] |
| 87 | 0 (0%) | n/a | 0 (0%) | n/a | < 10 (< 0·05%) | 4·8 [4·8,4·8] | 13 (< 0·05%) | 7·5 [7·1,8·3] |
| 88 | 0 (0%) | n/a | 0 (0%) | n/a | < 10 (< 0·05%) | 7·1 [6·5,7·7] | 16 (< 0·05%) | 7·4 [6·7,8·1] |
| 89 | 0 (0%) | n/a | 0 (0%) | n/a | < 10 (< 0·05%) | 7·9 [7·7,8·2] | 15 (< 0·05%) | 7·7 [7·0,8·4] |
| 90 | 0 (0%) | n/a | 0 (0%) | n/a | < 10 (< 0·05%) | 7·5 [7·3,7·6] | < 10 (< 0·05%) | 6·2 [6·1,6·8] |
| 91 | 0 (0%) | n/a | 0 (0%) | n/a | < 10 (< 0·05%) | 7·1 [6·7,7·5] | < 10 (< 0·05%) | 8·1 [6·7,8·5] |
| 92 | 0 (0%) | n/a | 0 (0%) | n/a | 0 (0%) | n/a | < 10 (< 0·05%) | 7·2 [6·5,7·9] |
| 93 | 0 (0%) | n/a | 0 (0%) | n/a | < 10 (< 0·05%) | 6·9 [6·5,7·5] | < 10 (< 0·05%) | 8·1 [7·2,8·6] |
| 94 | 0 (0%) | n/a | 0 (0%) | n/a | 0 (0%) | n/a | < 10 (< 0·05%) | 8·3 [7·8,8·6] |
| 95 | 0 (0%) | n/a | 0 (0%) | n/a | < 10 (< 0·05%) | 7·8 [7·8,7·8] | 13 (< 0·05%) | 7·7 [6·7,8·6] |
| 96 | 0 (0%) | n/a | 0 (0%) | n/a | 0 (0%) | n/a | < 10 (< 0·05%) | 7·0 [5·8,8·2] |
| 97 | 0 (0%) | n/a | 0 (0%) | n/a | < 10 (< 0·05%) | 8·3 [8·3,8·3] | < 10 (< 0·05%) | 8·3 [6·5,8·8] |
| 98 | 0 (0%) | n/a | 0 (0%) | n/a | < 10 (< 0·05%) | 9·0 [9·0,9·0] | < 10 (< 0·05%) | 7·9 [7·4,8·5] |
| 99 | 0 (0%) | n/a | 0 (0%) | n/a | 0 (0%) | n/a | < 10 (< 0·05%) | 7·9 [6·2,8·1] |
| 100 | 0 (0%) | n/a | 0 (0%) | n/a | < 10 (< 0·05%) | 8·1 [8·0,8·4] | < 10 (< 0·05%) | 7·5 [6·6,8·5] |
| 101 | 0 (0%) | n/a | 0 (0%) | n/a | < 10 (< 0·05%) | 4·7 [4·7,4·7] | < 10 (< 0·05%) | 6·2 [5·0,6·6] |
| 102 | 0 (0%) | n/a | 0 (0%) | n/a | 0 (0%) | n/a | < 10 (< 0·05%) | 8·6 [8·6,8·6] |
| 104 | 0 (0%) | n/a | 0 (0%) | n/a | < 10 (< 0·05%) | 5·5 [5·5,5·5] | < 10 (< 0·05%) | 8·3 [8·0,8·6] |
| 105 | 0 (0%) | n/a | 0 (0%) | n/a | 0 (0%) | n/a | < 10 (< 0·05%) | 8·2 [8·0,8·5] |
| 106 | 0 (0%) | n/a | 0 (0%) | n/a | 0 (0%) | n/a | < 10 (< 0·05%) | 4·8 [4·8,4·8] |
| 107 | 0 (0%) | n/a | 0 (0%) | n/a | 0 (0%) | n/a | < 10 (< 0·05%) | 7·8 [7·4,8·4] |
| 108 | 0 (0%) | n/a | 0 (0%) | n/a | 0 (0%) | n/a | < 10 (< 0·05%) | 6·1 [6·1,6·2] |
| 109 | 0 (0%) | n/a | 0 (0%) | n/a | 0 (0%) | n/a | < 10 (< 0·05%) | 7·2 [6·5,7·6] |
| 110 | 0 (0%) | n/a | 0 (0%) | n/a | 0 (0%) | n/a | < 10 (< 0·05%) | 5·4 [5·4,5·4] |
| 111 | 0 (0%) | n/a | 0 (0%) | n/a | 0 (0%) | n/a | < 10 (< 0·05%) | 4·9 [4·9,4·9] |
| 112 | 0 (0%) | n/a | 0 (0%) | n/a | 0 (0%) | n/a | < 10 (< 0·05%) | 8·4 [8·2,8·5] |
| 113 | 0 (0%) | n/a | 0 (0%) | n/a | 0 (0%) | n/a | < 10 (< 0·05%) | 7·4 [7·4,7·4] |
| 115 | 0 (0%) | n/a | 0 (0%) | n/a | 0 (0%) | n/a | < 10 (< 0·05%) | 8·1 [8·1,8·1] |
| 116 | 0 (0%) | n/a | 0 (0%) | n/a | 0 (0%) | n/a | < 10 (< 0·05%) | 8·6 [8·6,8·6] |
| 117 | 0 (0%) | n/a | 0 (0%) | n/a | 0 (0%) | n/a | < 10 (< 0·05%) | 8·5 [8·5,8·5] |
| 118 | 0 (0%) | n/a | 0 (0%) | n/a | < 10 (< 0·05%) | 8·5 [8·5,8·5] | < 10 (< 0·05%) | 8·1 [8·1,8·1] |
| 119 | 0 (0%) | n/a | 0 (0%) | n/a | 0 (0%) | n/a | < 10 (< 0·05%) | 7·7 [7·7,7·7] |
| 121 | 0 (0%) | n/a | 0 (0%) | n/a | 0 (0%) | n/a | < 10 (< 0·05%) | 8·1 [7·9,8·3] |
| 122 | 0 (0%) | n/a | 0 (0%) | n/a | 0 (0%) | n/a | < 10 (< 0·05%) | 6·3 [6·3,6·3] |
| 124 | 0 (0%) | n/a | 0 (0%) | n/a | 0 (0%) | n/a | < 10 (< 0·05%) | 6·0 [6·0,6·0] |
| 126 | 0 (0%) | n/a | 0 (0%) | n/a | 0 (0%) | n/a | < 10 (< 0·05%) | 9·0 [9·0,9·0] |
| 127 | 0 (0%) | n/a | 0 (0%) | n/a | 0 (0%) | n/a | < 10 (< 0·05%) | 8·1 [8·1,8·1] |
| 128 | 0 (0%) | n/a | 0 (0%) | n/a | 0 (0%) | n/a | < 10 (< 0·05%) | 7·5 [7·5,7·5] |
| 129 | 0 (0%) | n/a | 0 (0%) | n/a | 0 (0%) | n/a | < 10 (< 0·05%) | 8·7 [8·7,8·7] |
| 132 | 0 (0%) | n/a | 0 (0%) | n/a | 0 (0%) | n/a | < 10 (< 0·05%) | 8·7 [8·7,8·7] |
| 134 | 0 (0%) | n/a | 0 (0%) | n/a | < 10 (< 0·05%) | 7·5 [7·5,7·5] | < 10 (< 0·05%) | 7·1 [7·1,7·1] |
| 141 | 0 (0%) | n/a | 0 (0%) | n/a | 0 (0%) | n/a | < 10 (< 0·05%) | 8·8 [8·8,8·8] |
| 151 | 0 (0%) | n/a | 0 (0%) | n/a | 0 (0%) | n/a | < 10 (< 0·05%) | 7·0 [7·0,7·0] |
| 176 | 0 (0%) | n/a | 0 (0%) | n/a | 0 (0%) | n/a | < 10 (< 0·05%) | 9·0 [9·0,9·0] |

1. Table S7: Of the unique trajectories per cohort – for the primary and secondary analyses – the number of trajectories that contain at least one instance of a post-MI/initial diagnosis, grouped by ICD-10 chapter heading

|  | Trajectories constructed using primary diagnoses only (primary analysis) | | Trajectories constructed using both primary and secondary diagnoses (secondary analysis) | |
| --- | --- | --- | --- | --- |
| ICD-10 chapter heading | MI (number of unique trajectories: 28,799) | Matched controls (number of unique trajectories: 134,151) | MI (number of unique trajectories: 194,913) | Matched controls (number of unique trajectories: 744,381) |
| A00-B99: Infectious and Parasitic | 5,667 (19·7%) | 29,678 (22·1%) | 53,009 (27·2%) | 255,804 (34·4%) |
| C00-D48: Neoplasms | 8,926 (31·0%) | 51,994 (38·8%) | 37,894 (19·4%) | 248,576 (33·4%) |
| D50-D89: Blood and Immune | 5,876 (20·4%) | 26,881 (20·0%) | 51,511 (26·4%) | 213,765 (28·7%) |
| E00-E90: Endocrine/Nutritional/Metabolic | 3,378 (11·7%) | 17,123 (12·8%) | 136,591 (70·1%) | 427,860 (57·5%) |
| F00-F99: Mental and Behavioural | 1,781 (6·2%) | 10,958 (8·2%) | 85,717 (44·0%) | 309,330 (41·6%) |
| G00-G99: Nervous System | 3,476 (12·1%) | 20,945 (15·6%) | 35,508 (18·2%) | 191,350 (25·7%) |
| H00-H59: Eye and Adnexa | 7,919 (27·5%) | 39,011 (29·1%) | 35,792 (18·4%) | 181,939 (24·4%) |
| H60-H95: Ear and Mastoid Process | 809 (2·8%) | 4,314 (3·2%) | 12,770 (6·6%) | 63,689 (8·6%) |
| I00-I99: Circulatory System | 17,946 (62·3%) | 57,824 (43·1%) | 189,319 (97·1%) | 555,036 (74·6%) |
| J00-J99: Respiratory System | 12,766 (44·3%) | 59,099 (44·1%) | 111,942 (57·4%) | 426,883 (57·3%) |
| K00-K93: Digestive System | 16,831 (58·4%) | 81,676 (60·9%) | 106,718 (54·8%) | 472,502 (63·5%) |
| L00-L99: Skin/Subcutaneous Tissue | 4,289 (14·9%) | 27,647 (20·6%) | 35,104 (18·0%) | 184,825 (24·8%) |
| M00-M99: Musculoskeletal System | 9,940 (34·5%) | 57,484 (42·9%) | 85,118 (43·7%) | 398,800 (53·6%) |
| N00-N99: Genitourinary System | 10,202 (35·4%) | 55,621 (41·5%) | 93,674 (48·1%) | 404,538 (54·3%) |

1. Table S8: Occurrences of ICD-10 diagnosis codes (limited to three characters) within myocardial infarction (MI) cohort and matched control cohort disease trajectories, ordered by MI-primary analysis counts

|  | **Trajectories constructed using primary diagnoses only** | | | | **Trajectories constructed using primary and secondary diagnoses** | | | |
| --- | --- | --- | --- | --- | --- | --- | --- | --- |
|  | **MI Cohort** | | **Matched Control Cohort** | | **MI Cohort** | | **Matched Control Cohort** | |
| **ICD-10 Diagnosis (three-character code and description)** | **Occurrences (% of all diagnoses)** | **% of ICD-10 chapter** | **Occurrences (% of all diagnoses)** | **% of ICD-10 chapter** | **Occurrences (% of all diagnoses)** | **% of ICD-10 chapter** | **Occurrences (% of all diagnoses)** | **% of ICD-10 chapter** |
| **A00-B99 Certain infectious and parasitic diseases** | | | | | | | | |
| A09: Other gastroenteritis and colitis of infectious and unspecified origin | 4,953 (1·0%) | 36·8% | 27,768 (1·0%) | 31·6% | 16,805 (0·5%) | 20·7% | 79,518 (0·7%) | 17·9% |
| A41: Other sepsis | 4,552 (0·9%) | 33·8% | 35,478 (1·3%) | 40·4% | 13,774 (0·4%) | 17·0% | 78,983 (0·7%) | 17·8% |
| A08: Viral and other specified intestinal infections | 1,109 (0·2%) | 8·2% | 5,457 (0·2%) | 6·2% | 2,224 (0·1%) | 2·7% | 9,442 (0·1%) | 2·1% |
| A04: Other bacterial intestinal infections | 1,099 (0·2%) | 8·2% | 6,260 (0·2%) | 7·1% | 3,628 (0·1%) | 4·5% | 18,270 (0·2%) | 4·1% |
| B34: Viral infection of unspecified site | 747 (0·2%) | 5·6% | 3,302 (0·1%) | 3·8% | 1,225 (< 0·05%) | 1·5% | 5,568 (< 0·05%) | 1·3% |
| B37: Candidiasis | 314 (0·1%) | 2·3% | 2,482 (0·1%) | 2·8% | 5,253 (0·2%) | 6·5% | 33,417 (0·3%) | 7·5% |
| B02: Zoster [herpes zoster] | 239 (< 0·05%) | 1·8% | 1,800 (0·1%) | 2·0% | 979 (< 0·05%) | 1·2% | 5,926 (0·1%) | 1·3% |
| A49: Bacterial infection of unspecified site | 218 (< 0·05%) | 1·6% | 1,817 (0·1%) | 2·1% | 1,726 (0·1%) | 2·1% | 9,633 (0·1%) | 2·2% |
| A40: Streptococcal sepsis | 183 (< 0·05%) | 1·4% | 1,355 (< 0·05%) | 1·5% | 586 (< 0·05%) | 0·7% | 3,614 (< 0·05%) | 0·8% |
| B35: Dermatophytosis | 23 (< 0·05%) | 0·2% | 105 (< 0·05%) | 0·1% | 356 (< 0·05%) | 0·4% | 2,021 (< 0·05%) | 0·5% |
| B18: Chronic viral hepatitis | 19 (< 0·05%) | 0·1% | 263 (< 0·05%) | 0·3% | 449 (< 0·05%) | 0·6% | 3,917 (< 0·05%) | 0·9% |
| B95: Streptococcus and staphylococcus as the cause of diseases classified to other chapters | < 10 (< 0·05%) | 0·0% | < 10 (< 0·05%) | 0·0% | 8,176 (0·3%) | 10·1% | 48,976 (0·4%) | 11·0% |
| B96: Other specified bacterial agents as the cause of diseases classified to other chapters | < 10 (< 0·05%) | 0·0% | 19 (< 0·05%) | 0·0% | 24,321 (0·8%) | 30·0% | 128,380 (1·1%) | 28·9% |
| B07: Viral warts | 0 (0%) | 0·0% | 865 (< 0·05%) | 1·0% | 0 (0%) | 0·0% | 1,409 (< 0·05%) | 0·3% |
| B44: Aspergillosis | 0 (0%) | 0·0% | 546 (< 0·05%) | 0·6% | 0 (0%) | 0·0% | 3,185 (< 0·05%) | 0·7% |
| B00: Herpesviral [herpes simplex] infections | 0 (0%) | 0·0% | 317 (< 0·05%) | 0·4% | 0 (0%) | 0·0% | 2,007 (< 0·05%) | 0·5% |
| B98: Other specified infectious agents as the cause of disease classified to other chapters | 0 (0%) | 0·0% | 0 (0%) | 0·0% | 1,335 (< 0·05%) | 1·6% | 8,033 (0·1%) | 1·8% |
| B97: Viral agents as the cause of diseases classified to other chapters | 0 (0%) | 0·0% | 0 (0%) | 0·0% | 328 (< 0·05%) | 0·4% | 2,060 (< 0·05%) | 0·5% |
| **C00-D48 Neoplasms** | | | | | | | | |
| C44: Other malignant neoplasms of skin | 7,455 (1·5%) | 21·7% | 67,231 (2·4%) | 21·2% | 8,136 (0·3%) | 12·6% | 73,142 (0·7%) | 13·2% |
| D12: Benign neoplasm of colon, rectum, anus and anal canal | 6,012 (1·2%) | 17·5% | 49,703 (1·8%) | 15·7% | 7,212 (0·2%) | 11·1% | 58,780 (0·5%) | 10·6% |
| C34: Malignant neoplasm of bronchus and lung | 2,762 (0·6%) | 8·0% | 16,681 (0·6%) | 5·3% | 4,475 (0·1%) | 6·9% | 25,307 (0·2%) | 4·6% |
| C18: Malignant neoplasm of colon | 1,743 (0·4%) | 5·1% | 12,172 (0·4%) | 3·8% | 2,328 (0·1%) | 3·6% | 15,608 (0·1%) | 2·8% |
| C67: Malignant neoplasm of bladder | 1,719 (0·3%) | 5·0% | 11,876 (0·4%) | 3·8% | 2,264 (0·1%) | 3·5% | 14,784 (0·1%) | 2·7% |
| C61: Malignant neoplasm of prostate | 1,639 (0·3%) | 4·8% | 13,831 (0·5%) | 4·4% | 5,715 (0·2%) | 8·8% | 33,097 (0·3%) | 6·0% |
| C50: Malignant neoplasm of breast | 910 (0·2%) | 2·6% | 6,669 (0·2%) | 2·1% | 1,792 (0·1%) | 2·8% | 10,972 (0·1%) | 2·0% |
| C78: Secondary malignant neoplasm of respiratory and digestive organs | 844 (0·2%) | 2·5% | 9,512 (0·3%) | 3·0% | 5,057 (0·2%) | 7·8% | 51,207 (0·5%) | 9·2% |
| C15: Malignant neoplasm of oesophagus | 728 (0·1%) | 2·1% | 4,940 (0·2%) | 1·6% | 892 (< 0·05%) | 1·4% | 5,973 (0·1%) | 1·1% |
| C20: Malignant neoplasm of rectum | 693 (0·1%) | 2·0% | 5,276 (0·2%) | 1·7% | 887 (< 0·05%) | 1·4% | 6,596 (0·1%) | 1·2% |
| C79: Secondary malignant neoplasm of other and unspecified sites | 667 (0·1%) | 1·9% | 8,828 (0·3%) | 2·8% | 4,328 (0·1%) | 6·7% | 44,229 (0·4%) | 8·0% |
| C16: Malignant neoplasm of stomach | 663 (0·1%) | 1·9% | 4,416 (0·2%) | 1·4% | 824 (< 0·05%) | 1·3% | 5,431 (< 0·05%) | 1·0% |
| D37: Neoplasm of uncertain or unknown behaviour of oral cavity and digestive organs | 591 (0·1%) | 1·7% | 4,798 (0·2%) | 1·5% | 922 (< 0·05%) | 1·4% | 7,041 (0·1%) | 1·3% |
| D46: Myelodysplastic syndromes | 584 (0·1%) | 1·7% | 5,098 (0·2%) | 1·6% | 1,447 (< 0·05%) | 2·2% | 10,541 (0·1%) | 1·9% |
| C43: Malignant melanoma of skin | 478 (0·1%) | 1·4% | 3,863 (0·1%) | 1·2% | 592 (< 0·05%) | 0·9% | 4,765 (< 0·05%) | 0·9% |
| D41: Neoplasm of uncertain or unknown behaviour of urinary organs | 475 (0·1%) | 1·4% | 3,634 (0·1%) | 1·1% | 728 (< 0·05%) | 1·1% | 5,188 (< 0·05%) | 0·9% |
| C25: Malignant neoplasm of pancreas | 458 (0·1%) | 1·3% | 4,275 (0·2%) | 1·4% | 696 (< 0·05%) | 1·1% | 5,907 (0·1%) | 1·1% |
| D23: Other benign neoplasms of skin | 450 (0·1%) | 1·3% | 3,726 (0·1%) | 1·2% | 551 (< 0·05%) | 0·9% | 4,816 (< 0·05%) | 0·9% |
| C64: Malignant neoplasm of kidney, except renal pelvis | 447 (0·1%) | 1·3% | 3,651 (0·1%) | 1·2% | 868 (< 0·05%) | 1·3% | 6,063 (0·1%) | 1·1% |
| D04: Carcinoma in situ of skin | 432 (0·1%) | 1·3% | 4,627 (0·2%) | 1·5% | 671 (< 0·05%) | 1·0% | 7,097 (0·1%) | 1·3% |
| D17: Benign lipomatous neoplasm | 430 (0·1%) | 1·2% | 4,309 (0·2%) | 1·4% | 1,120 (< 0·05%) | 1·7% | 9,114 (0·1%) | 1·6% |
| C80: Malignant neoplasm without specification of site | 391 (0·1%) | 1·1% | 3,145 (0·1%) | 1·0% | 1,588 (0·1%) | 2·5% | 12,143 (0·1%) | 2·2% |
| C83: Non-follicular lymphoma | 368 (0·1%) | 1·1% | 3,855 (0·1%) | 1·2% | 494 (< 0·05%) | 0·8% | 4,938 (< 0·05%) | 0·9% |
| D47: Other neoplasms of uncertain or unknown behaviour of lymphoid, haematopoietic and related tissue | 313 (0·1%) | 0·9% | 2,473 (0·1%) | 0·8% | 1,340 (< 0·05%) | 2·1% | 8,279 (0·1%) | 1·5% |
| C77: Secondary and unspecified malignant neoplasm of lymph nodes | 309 (0·1%) | 0·9% | 3,076 (0·1%) | 1·0% | 2,737 (0·1%) | 4·2% | 30,461 (0·3%) | 5·5% |
| C85: Other and unspecified types of non-Hodgkin lymphoma | 307 (0·1%) | 0·9% | 3,078 (0·1%) | 1·0% | 743 (< 0·05%) | 1·1% | 6,181 (0·1%) | 1·1% |
| D22: Melanocytic naevi | 292 (0·1%) | 0·8% | 3,470 (0·1%) | 1·1% | 446 (< 0·05%) | 0·7% | 5,096 (< 0·05%) | 0·9% |
| C90: Multiple myeloma and malignant plasma cell neoplasms | 279 (0·1%) | 0·8% | 2,599 (0·1%) | 0·8% | 544 (< 0·05%) | 0·8% | 3,922 (< 0·05%) | 0·7% |
| C19: Malignant neoplasm of rectosigmoid junction | 275 (0·1%) | 0·8% | 2,867 (0·1%) | 0·9% | 401 (< 0·05%) | 0·6% | 4,144 (< 0·05%) | 0·7% |
| C22: Malignant neoplasm of liver and intrahepatic bile ducts | 258 (0·1%) | 0·7% | 2,808 (0·1%) | 0·9% | 401 (< 0·05%) | 0·6% | 4,167 (< 0·05%) | 0·8% |
| D35: Benign neoplasm of other and unspecified endocrine glands | 257 (0·1%) | 0·7% | 2,427 (0·1%) | 0·8% | 731 (< 0·05%) | 1·1% | 5,488 (< 0·05%) | 1·0% |
| D13: Benign neoplasm of other and ill-defined parts of digestive system | 250 (0·1%) | 0·7% | 2,216 (0·1%) | 0·7% | 442 (< 0·05%) | 0·7% | 3,657 (< 0·05%) | 0·7% |
| C92: Myeloid leukaemia | 239 (< 0·05%) | 0·7% | 2,090 (0·1%) | 0·7% | 422 (< 0·05%) | 0·7% | 3,160 (< 0·05%) | 0·6% |
| D18: Haemangioma and lymphangioma, any site | 198 (< 0·05%) | 0·6% | 1,491 (0·1%) | 0·5% | 626 (< 0·05%) | 1·0% | 4,357 (< 0·05%) | 0·8% |
| D25: Leiomyoma of uterus | 167 (< 0·05%) | 0·5% | 1,736 (0·1%) | 0·5% | 666 (< 0·05%) | 1·0% | 5,646 (0·1%) | 1·0% |
| D45: Polycythaemia vera | 143 (< 0·05%) | 0·4% | 759 (< 0·05%) | 0·2% | 480 (< 0·05%) | 0·7% | 1,777 (< 0·05%) | 0·3% |
| C91: Lymphoid leukaemia | 112 (< 0·05%) | 0·3% | 1,212 (< 0·05%) | 0·4% | 712 (< 0·05%) | 1·1% | 3,740 (< 0·05%) | 0·7% |
| D32: Benign neoplasm of meninges | 85 (< 0·05%) | 0·2% | 729 (< 0·05%) | 0·2% | 430 (< 0·05%) | 0·7% | 2,654 (< 0·05%) | 0·5% |
| C71: Malignant neoplasm of brain | 0 (0%) | 0·0% | 2,159 (0·1%) | 0·7% | 0 (0%) | 0·0% | 2,612 (< 0·05%) | 0·5% |
| C97: Malignant neoplasms of independent (primary) multiple sites | 0 (0%) | 0·0% | 1,944 (0·1%) | 0·6% | 0 (0%) | 0·0% | 2,662 (< 0·05%) | 0·5% |
| C54: Malignant neoplasm of corpus uteri | 0 (0%) | 0·0% | 1,797 (0·1%) | 0·6% | 0 (0%) | 0·0% | 2,175 (< 0·05%) | 0·4% |
| C45: Mesothelioma | 0 (0%) | 0·0% | 1,717 (0·1%) | 0·5% | 0 (0%) | 0·0% | 2,306 (< 0·05%) | 0·4% |
| D03: Melanoma in situ | 0 (0%) | 0·0% | 1,512 (0·1%) | 0·5% | 0 (0%) | 0·0% | 1,850 (< 0·05%) | 0·3% |
| C56: Malignant neoplasm of ovary | 0 (0%) | 0·0% | 1,507 (0·1%) | 0·5% | 0 (0%) | 0·0% | 2,314 (< 0·05%) | 0·4% |
| D09: Carcinoma in situ of other and unspecified sites | 0 (0%) | 0·0% | 1,369 (< 0·05%) | 0·4% | 0 (0%) | 0·0% | 2,034 (< 0·05%) | 0·4% |
| D48: Neoplasm of uncertain or unknown behaviour of other and unspecified sites | 0 (0%) | 0·0% | 1,292 (< 0·05%) | 0·4% | 0 (0%) | 0·0% | 2,077 (< 0·05%) | 0·4% |
| C32: Malignant neoplasm of larynx | 0 (0%) | 0·0% | 1,221 (< 0·05%) | 0·4% | 0 (0%) | 0·0% | 1,613 (< 0·05%) | 0·3% |
| C82: Follicular lymphoma | 0 (0%) | 0·0% | 1,178 (< 0·05%) | 0·4% | 0 (0%) | 0·0% | 1,567 (< 0·05%) | 0·3% |
| D36: Benign neoplasm of other and unspecified sites | 0 (0%) | 0·0% | 1,031 (< 0·05%) | 0·3% | 0 (0%) | 0·0% | 1,446 (< 0·05%) | 0·3% |
| D38: Neoplasm of uncertain or unknown behaviour of middle ear and respiratory and intrathoracic organs | 0 (0%) | 0·0% | 972 (< 0·05%) | 0·3% | 0 (0%) | 0·0% | 1,555 (< 0·05%) | 0·3% |
| C17: Malignant neoplasm of small intestine | 0 (0%) | 0·0% | 952 (< 0·05%) | 0·3% | 0 (0%) | 0·0% | 1,276 (< 0·05%) | 0·2% |
| C49: Malignant neoplasm of other connective and soft tissue | 0 (0%) | 0·0% | 882 (< 0·05%) | 0·3% | 0 (0%) | 0·0% | 1,198 (< 0·05%) | 0·2% |
| C24: Malignant neoplasm of other and unspecified parts of biliary tract | 0 (0%) | 0·0% | 879 (< 0·05%) | 0·3% | 0 (0%) | 0·0% | 1,126 (< 0·05%) | 0·2% |
| D27: Benign neoplasm of ovary | 0 (0%) | 0·0% | 832 (< 0·05%) | 0·3% | 0 (0%) | 0·0% | 1,295 (< 0·05%) | 0·2% |
| D07: Carcinoma in situ of other and unspecified genital organs | 0 (0%) | 0·0% | 801 (< 0·05%) | 0·3% | 0 (0%) | 0·0% | 1,163 (< 0·05%) | 0·2% |
| D05: Carcinoma in situ of breast | 0 (0%) | 0·0% | 786 (< 0·05%) | 0·2% | 0 (0%) | 0·0% | 1,248 (< 0·05%) | 0·2% |
| C02: Malignant neoplasm of other and unspecified parts of tongue | 0 (0%) | 0·0% | 738 (< 0·05%) | 0·2% | 0 (0%) | 0·0% | 1,038 (< 0·05%) | 0·2% |
| D11: Benign neoplasm of major salivary glands | 0 (0%) | 0·0% | 696 (< 0·05%) | 0·2% | 0 (0%) | 0·0% | 942 (< 0·05%) | 0·2% |
| D43: Neoplasm of uncertain or unknown behaviour of brain and central nervous system | 0 (0%) | 0·0% | 688 (< 0·05%) | 0·2% | 0 (0%) | 0·0% | 1,163 (< 0·05%) | 0·2% |
| C09: Malignant neoplasm of tonsil | 0 (0%) | 0·0% | 672 (< 0·05%) | 0·2% | 0 (0%) | 0·0% | 867 (< 0·05%) | 0·2% |
| C73: Malignant neoplasm of thyroid gland | 0 (0%) | 0·0% | 625 (< 0·05%) | 0·2% | 0 (0%) | 0·0% | 828 (< 0·05%) | 0·1% |
| C81: Hodgkin lymphoma | 0 (0%) | 0·0% | 614 (< 0·05%) | 0·2% | 0 (0%) | 0·0% | 1,050 (< 0·05%) | 0·2% |
| C26: Malignant neoplasm of other and ill-defined digestive organs | 0 (0%) | 0·0% | 535 (< 0·05%) | 0·2% | 0 (0%) | 0·0% | 1,812 (< 0·05%) | 0·3% |
| **D50-D89 Diseases of the blood and blood-forming organs and certain disorders involving the immune mechanism** | | | | | | | | |
| D50: Iron deficiency anaemia | 7,268 (1·5%) | 44·4% | 32,618 (1·2%) | 36·4% | 25,112 (0·8%) | 29·8% | 98,511 (0·9%) | 26·0% |
| D64: Other anaemias | 6,922 (1·4%) | 42·3% | 35,749 (1·3%) | 39·9% | 41,403 (1·3%) | 49·1% | 166,755 (1·5%) | 44·0% |
| D75: Other diseases of blood and blood-forming organs | 448 (0·1%) | 2·7% | 5,495 (0·2%) | 6·1% | 1,591 (0·1%) | 1·9% | 10,874 (0·1%) | 2·9% |
| D63: Anaemia in chronic diseases classified elsewhere | 443 (0·1%) | 2·7% | 3,354 (0·1%) | 3·7% | 1,404 (< 0·05%) | 1·7% | 10,142 (0·1%) | 2·7% |
| D69: Purpura and other haemorrhagic conditions | 280 (0·1%) | 1·7% | 1,927 (0·1%) | 2·1% | 3,555 (0·1%) | 4·2% | 18,241 (0·2%) | 4·8% |
| D70: Agranulocytosis | 278 (0·1%) | 1·7% | 4,402 (0·2%) | 4·9% | 1,758 (0·1%) | 2·1% | 22,478 (0·2%) | 5·9% |
| D61: Other aplastic anaemias | 183 (< 0·05%) | 1·1% | 1,541 (0·1%) | 1·7% | 799 (< 0·05%) | 0·9% | 6,476 (0·1%) | 1·7% |
| D52: Folate deficiency anaemia | 172 (< 0·05%) | 1·1% | 942 (< 0·05%) | 1·1% | 1,538 (< 0·05%) | 1·8% | 6,438 (0·1%) | 1·7% |
| D89: Other disorders involving the immune mechanism, not elsewhere classified | 77 (< 0·05%) | 0·5% | 649 (< 0·05%) | 0·7% | 335 (< 0·05%) | 0·4% | 2,323 (< 0·05%) | 0·6% |
| D68: Other coagulation defects | 60 (< 0·05%) | 0·4% | 345 (< 0·05%) | 0·4% | 1,278 (< 0·05%) | 1·5% | 5,000 (< 0·05%) | 1·3% |
| D72: Other disorders of white blood cells | 55 (< 0·05%) | 0·3% | 311 (< 0·05%) | 0·3% | 569 (< 0·05%) | 0·7% | 2,368 (< 0·05%) | 0·6% |
| D53: Other nutritional anaemias | 44 (< 0·05%) | 0·3% | 270 (< 0·05%) | 0·3% | 456 (< 0·05%) | 0·5% | 1,955 (< 0·05%) | 0·5% |
| D51: Vitamin B12 deficiency anaemia | 43 (< 0·05%) | 0·3% | 291 (< 0·05%) | 0·3% | 2,391 (0·1%) | 2·8% | 12,136 (0·1%) | 3·2% |
| D73: Diseases of spleen | 31 (< 0·05%) | 0·2% | 258 (< 0·05%) | 0·3% | 1,183 (< 0·05%) | 1·4% | 8,131 (0·1%) | 2·1% |
| D86: Sarcoidosis | 31 (< 0·05%) | 0·2% | 390 (< 0·05%) | 0·4% | 328 (< 0·05%) | 0·4% | 2,157 (< 0·05%) | 0·6% |
| D62: Acute posthaemorrhagic anaemia | 20 (< 0·05%) | 0·1% | 0 (0%) | 0·0% | 390 (< 0·05%) | 0·5% | 0 (0%) | 0·0% |
| D59: Acquired haemolytic anaemia | 0 (0%) | 0·0% | 420 (< 0·05%) | 0·5% | 0 (0%) | 0·0% | 1,127 (< 0·05%) | 0·3% |
| D80: Immunodeficiency with predominantly antibody defects | 0 (0%) | 0·0% | 382 (< 0·05%) | 0·4% | 0 (0%) | 0·0% | 1,169 (< 0·05%) | 0·3% |
| D57: Sickle-cell disorders | 0 (0%) | 0·0% | 272 (< 0·05%) | 0·3% | 0 (0%) | 0·0% | 1,273 (< 0·05%) | 0·3% |
| D56: Thalassaemia | 0 (0%) | 0·0% | 30 (< 0·05%) | 0·0% | 308 (< 0·05%) | 0·4% | 1,196 (< 0·05%) | 0·3% |
| **E00-E90 Endocrine, nutritional and metabolic diseases** | | | | | | | | |
| E87: Other disorders of fluid, electrolyte and acid-base balance | 2,854 (0·6%) | 41·3% | 15,624 (0·6%) | 35·0% | 42,529 (1·4%) | 13·1% | 166,654 (1·5%) | 18·5% |
| E86: Volume depletion | 1,108 (0·2%) | 16·0% | 7,813 (0·3%) | 17·5% | 19,051 (0·6%) | 5·9% | 106,439 (1·0%) | 11·8% |
| E16: Other disorders of pancreatic internal secretion | 909 (0·2%) | 13·1% | 5,445 (0·2%) | 12·2% | 4,078 (0·1%) | 1·3% | 19,858 (0·2%) | 2·2% |
| E83: Disorders of mineral metabolism | 608 (0·1%) | 8·8% | 4,534 (0·2%) | 10·1% | 4,587 (0·1%) | 1·4% | 28,083 (0·3%) | 3·1% |
| E11: Type 2 diabetes mellitus | 318 (0·1%) | 4·6% | 1,939 (0·1%) | 4·3% | 52,919 (1·7%) | 16·4% | 117,885 (1·1%) | 13·1% |
| E22: Hyperfunction of pituitary gland | 136 (< 0·05%) | 2·0% | 814 (< 0·05%) | 1·8% | 768 (< 0·05%) | 0·2% | 4,137 (< 0·05%) | 0·5% |
| E14: Unspecified diabetes mellitus | 123 (< 0·05%) | 1·8% | 970 (< 0·05%) | 2·2% | 4,461 (0·1%) | 1·4% | 24,110 (0·2%) | 2·7% |
| E61: Deficiency of other nutrient elements | 115 (< 0·05%) | 1·7% | 729 (< 0·05%) | 1·6% | 473 (< 0·05%) | 0·1% | 2,826 (< 0·05%) | 0·3% |
| E04: Other nontoxic goitre | 111 (< 0·05%) | 1·6% | 1,126 (< 0·05%) | 2·5% | 893 (< 0·05%) | 0·3% | 4,810 (< 0·05%) | 0·5% |
| E10: Type 1 diabetes mellitus | 103 (< 0·05%) | 1·5% | 785 (< 0·05%) | 1·8% | 4,455 (0·1%) | 1·4% | 17,249 (0·2%) | 1·9% |
| E05: Thyrotoxicosis [hyperthyroidism] | 92 (< 0·05%) | 1·3% | 524 (< 0·05%) | 1·2% | 2,271 (0·1%) | 0·7% | 9,351 (0·1%) | 1·0% |
| E27: Other disorders of adrenal gland | 89 (< 0·05%) | 1·3% | 784 (< 0·05%) | 1·8% | 594 (< 0·05%) | 0·2% | 3,564 (< 0·05%) | 0·4% |
| E21: Hyperparathyroidism and other disorders of parathyroid gland | 84 (< 0·05%) | 1·2% | 683 (< 0·05%) | 1·5% | 890 (< 0·05%) | 0·3% | 5,444 (< 0·05%) | 0·6% |
| E66: Obesity | 59 (< 0·05%) | 0·9% | 614 (< 0·05%) | 1·4% | 29,511 (1·0%) | 9·1% | 84,411 (0·8%) | 9·3% |
| E03: Other hypothyroidism | 55 (< 0·05%) | 0·8% | 181 (< 0·05%) | 0·4% | 18,130 (0·6%) | 5·6% | 62,220 (0·6%) | 6·9% |
| E46: Unspecified protein-energy malnutrition | 37 (< 0·05%) | 0·5% | 372 (< 0·05%) | 0·8% | 619 (< 0·05%) | 0·2% | 4,375 (< 0·05%) | 0·5% |
| E55: Vitamin D deficiency | 31 (< 0·05%) | 0·4% | 211 (< 0·05%) | 0·5% | 2,171 (0·1%) | 0·7% | 12,780 (0·1%) | 1·4% |
| E53: Deficiency of other B group vitamins | 28 (< 0·05%) | 0·4% | 226 (< 0·05%) | 0·5% | 3,134 (0·1%) | 1·0% | 18,053 (0·2%) | 2·0% |
| E88: Other metabolic disorders | 17 (< 0·05%) | 0·2% | 234 (< 0·05%) | 0·5% | 954 (< 0·05%) | 0·3% | 5,737 (0·1%) | 0·6% |
| E07: Other disorders of thyroid | 15 (< 0·05%) | 0·2% | 111 (< 0·05%) | 0·2% | 313 (< 0·05%) | 0·1% | 1,766 (< 0·05%) | 0·2% |
| E78: Disorders of lipoprotein metabolism and other lipidaemias | 14 (< 0·05%) | 0·2% | 77 (< 0·05%) | 0·2% | 129,678 (4·2%) | 40·1% | 191,781 (1·7%) | 21·2% |
| E89: Postprocedural endocrine and metabolic disorders, not elsewhere classified | 10 (< 0·05%) | 0·1% | 153 (< 0·05%) | 0·3% | 1,046 (< 0·05%) | 0·3% | 6,159 (0·1%) | 0·7% |
| E23: Hypofunction and other disorders of pituitary gland | 0 (0%) | 0·0% | 417 (< 0·05%) | 0·9% | 0 (0%) | 0·0% | 1,870 (< 0·05%) | 0·2% |
| E13: Other specified diabetes mellitus | 0 (0%) | 0·0% | 259 (< 0·05%) | 0·6% | 0 (0%) | 0·0% | 2,159 (< 0·05%) | 0·2% |
| E80: Disorders of porphyrin and bilirubin metabolism | 0 (0%) | 0·0% | 50 (< 0·05%) | 0·1% | 0 (0%) | 0·0% | 1,448 (< 0·05%) | 0·2% |
| **F00-F99 Mental, behavioural and neuro-developmental disorders** | | | | | | | | |
| F05: Delirium, not induced by alcohol and other psychoactive substances | 943 (0·2%) | 22·6% | 7,245 (0·3%) | 20·3% | 8,518 (0·3%) | 3·9% | 45,691 (0·4%) | 5·9% |
| F10: Mental and behavioural disorders due to use of alcohol | 771 (0·2%) | 18·5% | 11,312 (0·4%) | 31·8% | 13,009 (0·4%) | 6·0% | 89,059 (0·8%) | 11·4% |
| F41: Other anxiety disorders | 722 (0·1%) | 17·3% | 2,022 (0·1%) | 5·7% | 14,584 (0·5%) | 6·7% | 57,399 (0·5%) | 7·4% |
| F01: Vascular dementia | 401 (0·1%) | 9·6% | 2,731 (0·1%) | 7·7% | 4,979 (0·2%) | 2·3% | 27,865 (0·2%) | 3·6% |
| F03: Unspecified dementia | 385 (0·1%) | 9·2% | 2,708 (0·1%) | 7·6% | 13,398 (0·4%) | 6·2% | 73,344 (0·7%) | 9·4% |
| F32: Depressive episode | 249 (0·1%) | 6·0% | 1,888 (0·1%) | 5·3% | 20,629 (0·7%) | 9·5% | 94,527 (0·8%) | 12·1% |
| F33: Recurrent depressive disorder | 129 (< 0·05%) | 3·1% | 1,105 (< 0·05%) | 3·1% | 425 (< 0·05%) | 0·2% | 3,103 (< 0·05%) | 0·4% |
| F43: Reaction to severe stress, and adjustment disorders | 123 (< 0·05%) | 3·0% | 843 (< 0·05%) | 2·4% | 580 (< 0·05%) | 0·3% | 2,914 (< 0·05%) | 0·4% |
| F06: Other mental disorders due to brain damage and dysfunction and to physical disease | 113 (< 0·05%) | 2·7% | 780 (< 0·05%) | 2·2% | 1,970 (0·1%) | 0·9% | 10,452 (0·1%) | 1·3% |
| F00: Dementia in Alzheimer's disease (G30·-+) | 84 (< 0·05%) | 2·0% | 863 (< 0·05%) | 2·4% | 4,328 (0·1%) | 2·0% | 33,410 (0·3%) | 4·3% |
| F31: Bipolar affective disorder | 56 (< 0·05%) | 1·3% | 462 (< 0·05%) | 1·3% | 817 (< 0·05%) | 0·4% | 4,529 (< 0·05%) | 0·6% |
| F22: Persistent delusional disorders | 56 (< 0·05%) | 1·3% | 491 (< 0·05%) | 1·4% | 357 (< 0·05%) | 0·2% | 2,260 (< 0·05%) | 0·3% |
| F20: Schizophrenia | 51 (< 0·05%) | 1·2% | 468 (< 0·05%) | 1·3% | 1,011 (< 0·05%) | 0·5% | 4,895 (< 0·05%) | 0·6% |
| F60: Specific personality disorders | 32 (< 0·05%) | 0·8% | 378 (< 0·05%) | 1·1% | 305 (< 0·05%) | 0·1% | 2,357 (< 0·05%) | 0·3% |
| F11: Mental and behavioural disorders due to use of opioids | 16 (< 0·05%) | 0·4% | 275 (< 0·05%) | 0·8% | 798 (< 0·05%) | 0·4% | 6,805 (0·1%) | 0·9% |
| F17: Mental and behavioural disorders due to use of tobacco | 11 (< 0·05%) | 0·3% | 83 (< 0·05%) | 0·2% | 127,304 (4·1%) | 58·6% | 294,850 (2·6%) | 37·9% |
| F02: Dementia in other diseases classified elsewhere | < 10 (< 0·05%) | 0·2% | 125 (< 0·05%) | 0·4% | 499 (< 0·05%) | 0·2% | 4,602 (< 0·05%) | 0·6% |
| F12: Mental and behavioural disorders due to use of cannabinoids | < 10 (< 0·05%) | 0·2% | 62 (< 0·05%) | 0·2% | 1,148 (< 0·05%) | 0·5% | 2,951 (< 0·05%) | 0·4% |
| F14: Mental and behavioural disorders due to use of cocaine | < 10 (< 0·05%) | 0·1% | 0 (0%) | 0·0% | 567 (< 0·05%) | 0·3% | 0 (0%) | 0·0% |
| F40: Phobic anxiety disorders | < 10 (< 0·05%) | 0·1% | 26 (< 0·05%) | 0·1% | 1,148 (< 0·05%) | 0·5% | 5,400 (< 0·05%) | 0·7% |
| F81: Specific developmental disorders of scholastic skills | < 10 (< 0·05%) | 0·0% | 25 (< 0·05%) | 0·1% | 725 (< 0·05%) | 0·3% | 4,687 (< 0·05%) | 0·6% |
| F99: Mental disorder, not otherwise specified | 0 (0%) | 0·0% | 601 (< 0·05%) | 1·7% | 0 (0%) | 0·0% | 1,432 (< 0·05%) | 0·2% |
| F45: Somatoform disorders | 0 (0%) | 0·0% | 466 (< 0·05%) | 1·3% | 0 (0%) | 0·0% | 1,139 (< 0·05%) | 0·1% |
| F29: Unspecified nonorganic psychosis | 0 (0%) | 0·0% | 337 (< 0·05%) | 0·9% | 0 (0%) | 0·0% | 1,784 (< 0·05%) | 0·2% |
| F19: Mental and behavioural disorders due to multiple drug use and use of other psychoactive substances | 0 (0%) | 0·0% | 311 (< 0·05%) | 0·9% | 0 (0%) | 0·0% | 1,957 (< 0·05%) | 0·3% |
| F79: Unspecified mental retardation | 0 (0%) | 0·0% | < 10 (< 0·05%) | 0·0% | 0 (0%) | 0·0% | 1,487 (< 0·05%) | 0·2% |
| **G00-G99 Diseases of the nervous system** | | | | | | | | |
| G45: Transient cerebral ischaemic attacks and related syndromes | 2,425 (0·5%) | 27·0% | 12,911 (0·5%) | 18·0% | 3,477 (0·1%) | 6·8% | 16,106 (0·1%) | 5·0% |
| G56: Mononeuropathies of upper limb | 2,152 (0·4%) | 24·0% | 14,699 (0·5%) | 20·5% | 2,594 (0·1%) | 5·1% | 17,386 (0·2%) | 5·4% |
| G47: Sleep disorders | 1,085 (0·2%) | 12·1% | 10,842 (0·4%) | 15·1% | 8,084 (0·3%) | 15·9% | 57,805 (0·5%) | 17·9% |
| G40: Epilepsy | 480 (0·1%) | 5·3% | 3,656 (0·1%) | 5·1% | 4,206 (0·1%) | 8·3% | 22,725 (0·2%) | 7·0% |
| G93: Other disorders of brain | 403 (0·1%) | 4·5% | 1,936 (0·1%) | 2·7% | 4,421 (0·1%) | 8·7% | 13,025 (0·1%) | 4·0% |
| G43: Migraine | 328 (0·1%) | 3·7% | 2,382 (0·1%) | 3·3% | 1,466 (< 0·05%) | 2·9% | 7,458 (0·1%) | 2·3% |
| G81: Hemiplegia | 256 (0·1%) | 2·9% | 1,591 (0·1%) | 2·2% | 4,035 (0·1%) | 7·9% | 19,717 (0·2%) | 6·1% |
| G30: Alzheimer's disease | 253 (0·1%) | 2·8% | 2,072 (0·1%) | 2·9% | 4,593 (0·1%) | 9·0% | 28,097 (0·3%) | 8·7% |
| G51: Facial nerve disorders | 243 (< 0·05%) | 2·7% | 2,770 (0·1%) | 3·9% | 1,119 (< 0·05%) | 2·2% | 7,890 (0·1%) | 2·4% |
| G44: Other headache syndromes | 204 (< 0·05%) | 2·3% | 1,503 (0·1%) | 2·1% | 384 (< 0·05%) | 0·8% | 2,280 (< 0·05%) | 0·7% |
| G62: Other polyneuropathies | 139 (< 0·05%) | 1·5% | 1,463 (0·1%) | 2·0% | 1,736 (0·1%) | 3·4% | 12,234 (0·1%) | 3·8% |
| G31: Other degenerative diseases of nervous system, not elsewhere classified | 123 (< 0·05%) | 1·4% | 1,099 (< 0·05%) | 1·5% | 3,528 (0·1%) | 6·9% | 21,587 (0·2%) | 6·7% |
| G50: Disorders of trigeminal nerve | 119 (< 0·05%) | 1·3% | 1,149 (< 0·05%) | 1·6% | 749 (< 0·05%) | 1·5% | 4,153 (< 0·05%) | 1·3% |
| G95: Other diseases of spinal cord | 110 (< 0·05%) | 1·2% | 1,459 (0·1%) | 2·0% | 507 (< 0·05%) | 1·0% | 4,940 (< 0·05%) | 1·5% |
| G57: Mononeuropathies of lower limb | 104 (< 0·05%) | 1·2% | 998 (< 0·05%) | 1·4% | 196 (< 0·05%) | 0·4% | 1,645 (< 0·05%) | 0·5% |
| G55: Nerve root and plexus compressions in diseases classified elsewhere | 98 (< 0·05%) | 1·1% | 961 (< 0·05%) | 1·3% | 1,585 (0·1%) | 3·1% | 15,859 (0·1%) | 4·9% |
| G12: Spinal muscular atrophy and related syndromes | 79 (< 0·05%) | 0·9% | 694 (< 0·05%) | 1·0% | 256 (< 0·05%) | 0·5% | 1,675 (< 0·05%) | 0·5% |
| G20: Parkinson's disease | 78 (< 0·05%) | 0·9% | 795 (< 0·05%) | 1·1% | 2,633 (0·1%) | 5·2% | 14,676 (0·1%) | 4·6% |
| G83: Other paralytic syndromes | 73 (< 0·05%) | 0·8% | 671 (< 0·05%) | 0·9% | 393 (< 0·05%) | 0·8% | 3,082 (< 0·05%) | 1·0% |
| G25: Other extrapyramidal and movement disorders | 68 (< 0·05%) | 0·8% | 635 (< 0·05%) | 0·9% | 1,267 (< 0·05%) | 2·5% | 6,850 (0·1%) | 2·1% |
| G91: Hydrocephalus | 54 (< 0·05%) | 0·6% | 613 (< 0·05%) | 0·9% | 460 (< 0·05%) | 0·9% | 3,296 (< 0·05%) | 1·0% |
| G99: Other disorders of nervous system in diseases classified elsewhere | 43 (< 0·05%) | 0·5% | 547 (< 0·05%) | 0·8% | 744 (< 0·05%) | 1·5% | 7,534 (0·1%) | 2·3% |
| G82: Paraplegia and tetraplegia | 31 (< 0·05%) | 0·3% | 292 (< 0·05%) | 0·4% | 365 (< 0·05%) | 0·7% | 2,562 (< 0·05%) | 0·8% |
| G35: Multiple sclerosis | 21 (< 0·05%) | 0·2% | 296 (< 0·05%) | 0·4% | 583 (< 0·05%) | 1·1% | 2,257 (< 0·05%) | 0·7% |
| G63: Polyneuropathy in diseases classified elsewhere | < 10 (< 0·05%) | 0·1% | 56 (< 0·05%) | 0·1% | 1,048 (< 0·05%) | 2·1% | 6,573 (0·1%) | 2·0% |
| G41: Status epilepticus | 0 (0%) | 0·0% | 1,305 (< 0·05%) | 1·8% | 0 (0%) | 0·0% | 1,954 (< 0·05%) | 0·6% |
| G04: Encephalitis, myelitis and encephalomyelitis | 0 (0%) | 0·0% | 681 (< 0·05%) | 1·0% | 0 (0%) | 0·0% | 1,466 (< 0·05%) | 0·5% |
| G70: Myasthenia gravis and other myoneural disorders | 0 (0%) | 0·0% | 630 (< 0·05%) | 0·9% | 0 (0%) | 0·0% | 3,470 (< 0·05%) | 1·1% |
| G58: Other mononeuropathies | 0 (0%) | 0·0% | 547 (< 0·05%) | 0·8% | 0 (0%) | 0·0% | 1,520 (< 0·05%) | 0·5% |
| G61: Inflammatory polyneuropathy | 0 (0%) | 0·0% | 540 (< 0·05%) | 0·8% | 0 (0%) | 0·0% | 1,030 (< 0·05%) | 0·3% |
| G54: Nerve root and plexus disorders | 0 (0%) | 0·0% | 514 (< 0·05%) | 0·7% | 0 (0%) | 0·0% | 1,653 (< 0·05%) | 0·5% |
| G90: Disorders of autonomic nervous system | 0 (0%) | 0·0% | 361 (< 0·05%) | 0·5% | 0 (0%) | 0·0% | 1,773 (< 0·05%) | 0·5% |
| G24: Dystonia | 0 (0%) | 0·0% | 344 (< 0·05%) | 0·5% | 0 (0%) | 0·0% | 1,281 (< 0·05%) | 0·4% |
| G72: Other myopathies | 0 (0%) | 0·0% | 323 (< 0·05%) | 0·5% | 0 (0%) | 0·0% | 1,416 (< 0·05%) | 0·4% |
| G21: Secondary parkinsonism | 0 (0%) | 0·0% | 222 (< 0·05%) | 0·3% | 0 (0%) | 0·0% | 1,423 (< 0·05%) | 0·4% |
| G46: Vascular syndromes of brain in cerebrovascular diseases (I60-I67+) | 0 (0%) | 0·0% | 63 (< 0·05%) | 0·1% | 0 (0%) | 0·0% | 1,390 (< 0·05%) | 0·4% |
| G80: Cerebral palsy | 0 (0%) | 0·0% | 20 (< 0·05%) | 0·0% | 0 (0%) | 0·0% | 800 (< 0·05%) | 0·2% |
| G59: Mononeuropathy in diseases classified elsewhere | 0 (0%) | 0·0% | < 10 (< 0·05%) | 0·0% | 356 (< 0·05%) | 0·7% | 1,922 (< 0·05%) | 0·6% |
| **H00-H59 Eye and adnexa** | | | | | | | | |
| H26: Other cataract | 12,503 (2·5%) | 43·3% | 71,624 (2·5%) | 38·7% | 15,119 (0·5%) | 25·5% | 86,994 (0·8%) | 23·0% |
| H25: Senile cataract | 9,436 (1·9%) | 32·7% | 52,138 (1·8%) | 28·2% | 10,002 (0·3%) | 16·9% | 57,095 (0·5%) | 15·1% |
| H02: Other disorders of eyelid | 2,291 (0·5%) | 7·9% | 18,588 (0·7%) | 10·0% | 3,039 (0·1%) | 5·1% | 23,633 (0·2%) | 6·2% |
| H35: Other retinal disorders | 1,167 (0·2%) | 4·0% | 8,119 (0·3%) | 4·4% | 6,146 (0·2%) | 10·4% | 37,752 (0·3%) | 10·0% |
| H04: Disorders of lacrimal system | 589 (0·1%) | 2·0% | 4,493 (0·2%) | 2·4% | 1,103 (< 0·05%) | 1·9% | 8,521 (0·1%) | 2·3% |
| H33: Retinal detachments and breaks | 575 (0·1%) | 2·0% | 5,576 (0·2%) | 3·0% | 803 (< 0·05%) | 1·4% | 7,539 (0·1%) | 2·0% |
| H40: Glaucoma | 529 (0·1%) | 1·8% | 4,374 (0·2%) | 2·4% | 6,443 (0·2%) | 10·9% | 39,946 (0·4%) | 10·6% |
| H53: Visual disturbances | 342 (0·1%) | 1·2% | 2,371 (0·1%) | 1·3% | 3,081 (0·1%) | 5·2% | 18,064 (0·2%) | 4·8% |
| H34: Retinal vascular occlusions | 276 (0·1%) | 1·0% | 1,949 (0·1%) | 1·1% | 676 (< 0·05%) | 1·1% | 4,400 (< 0·05%) | 1·2% |
| H43: Disorders of vitreous body | 230 (< 0·05%) | 0·8% | 1,833 (0·1%) | 1·0% | 690 (< 0·05%) | 1·2% | 5,466 (< 0·05%) | 1·4% |
| H18: Other disorders of cornea | 207 (< 0·05%) | 0·7% | 1,919 (0·1%) | 1·0% | 694 (< 0·05%) | 1·2% | 5,716 (0·1%) | 1·5% |
| H11: Other disorders of conjunctiva | 138 (< 0·05%) | 0·5% | 1,243 (< 0·05%) | 0·7% | 661 (< 0·05%) | 1·1% | 3,636 (< 0·05%) | 1·0% |
| H00: Hordeolum and chalazion | 133 (< 0·05%) | 0·5% | 1,557 (0·1%) | 0·8% | 188 (< 0·05%) | 0·3% | 2,045 (< 0·05%) | 0·5% |
| H36: Retinal disorders in diseases classified elsewhere | 107 (< 0·05%) | 0·4% | 621 (< 0·05%) | 0·3% | 3,087 (0·1%) | 5·2% | 15,498 (0·1%) | 4·1% |
| H57: Other disorders of eye and adnexa | 106 (< 0·05%) | 0·4% | 819 (< 0·05%) | 0·4% | 509 (< 0·05%) | 0·9% | 3,564 (< 0·05%) | 0·9% |
| H44: Disorders of globe | 70 (< 0·05%) | 0·2% | 523 (< 0·05%) | 0·3% | 290 (< 0·05%) | 0·5% | 2,243 (< 0·05%) | 0·6% |
| H21: Other disorders of iris and ciliary body | 43 (< 0·05%) | 0·1% | 372 (< 0·05%) | 0·2% | 394 (< 0·05%) | 0·7% | 3,253 (< 0·05%) | 0·9% |
| H01: Other inflammation of eyelid | 43 (< 0·05%) | 0·1% | 313 (< 0·05%) | 0·2% | 411 (< 0·05%) | 0·7% | 3,619 (< 0·05%) | 1·0% |
| H54: Visual impairment including blindness (binocular or monocular) | 24 (< 0·05%) | 0·1% | 220 (< 0·05%) | 0·1% | 4,365 (0·1%) | 7·4% | 21,736 (0·2%) | 5·7% |
| H10: Conjunctivitis | 19 (< 0·05%) | 0·1% | 224 (< 0·05%) | 0·1% | 825 (< 0·05%) | 1·4% | 5,248 (< 0·05%) | 1·4% |
| H52: Disorders of refraction and accommodation | 18 (< 0·05%) | 0·1% | 287 (< 0·05%) | 0·2% | 734 (< 0·05%) | 1·2% | 6,646 (0·1%) | 1·8% |
| H50: Other strabismus | 0 (0%) | 0·0% | 1,217 (< 0·05%) | 0·7% | 0 (0%) | 0·0% | 2,206 (< 0·05%) | 0·6% |
| H59: Postprocedural disorders of eye and adnexa, not elsewhere classified | 0 (0%) | 0·0% | 1,150 (< 0·05%) | 0·6% | 0 (0%) | 0·0% | 1,597 (< 0·05%) | 0·4% |
| H16: Keratitis | 0 (0%) | 0·0% | 943 (< 0·05%) | 0·5% | 0 (0%) | 0·0% | 1,742 (< 0·05%) | 0·5% |
| H27: Other disorders of lens | 0 (0%) | 0·0% | 789 (< 0·05%) | 0·4% | 0 (0%) | 0·0% | 1,372 (< 0·05%) | 0·4% |
| H05: Disorders of orbit | 0 (0%) | 0·0% | 584 (< 0·05%) | 0·3% | 0 (0%) | 0·0% | 1,384 (< 0·05%) | 0·4% |
| H49: Paralytic strabismus | 0 (0%) | 0·0% | 511 (< 0·05%) | 0·3% | 0 (0%) | 0·0% | 1,711 (< 0·05%) | 0·5% |
| H20: Iridocyclitis | 0 (0%) | 0·0% | 307 (< 0·05%) | 0·2% | 0 (0%) | 0·0% | 1,862 (< 0·05%) | 0·5% |
| H31: Other disorders of choroid | 0 (0%) | 0·0% | 290 (< 0·05%) | 0·2% | 0 (0%) | 0·0% | 1,510 (< 0·05%) | 0·4% |
| H47: Other disorders of optic [2nd] nerve and visual pathways | 0 (0%) | 0·0% | 210 (< 0·05%) | 0·1% | 0 (0%) | 0·0% | 1,346 (< 0·05%) | 0·4% |
| H55: Nystagmus and other irregular eye movements | 0 (0%) | 0·0% | 34 (< 0·05%) | 0·0% | 0 (0%) | 0·0% | 1,142 (< 0·05%) | 0·3% |
| **H60-H95 Ear and mastoid process** | | | | | | | | |
| H81: Disorders of vestibular function | 395 (0·1%) | 24·9% | 2,051 (0·1%) | 18·3% | 1,562 (0·1%) | 10·5% | 7,040 (0·1%) | 8·6% |
| H83: Other diseases of inner ear | 296 (0·1%) | 18·6% | 1,526 (0·1%) | 13·6% | 477 (< 0·05%) | 3·2% | 2,360 (< 0·05%) | 2·9% |
| H61: Other disorders of external ear | 239 (< 0·05%) | 15·1% | 1,797 (0·1%) | 16·0% | 597 (< 0·05%) | 4·0% | 4,118 (< 0·05%) | 5·0% |
| H65: Nonsuppurative otitis media | 206 (< 0·05%) | 13·0% | 1,557 (0·1%) | 13·9% | 251 (< 0·05%) | 1·7% | 1,893 (< 0·05%) | 2·3% |
| H60: Otitis externa | 129 (< 0·05%) | 8·1% | 944 (< 0·05%) | 8·4% | 296 (< 0·05%) | 2·0% | 1,782 (< 0·05%) | 2·2% |
| H66: Suppurative and unspecified otitis media | 102 (< 0·05%) | 6·4% | 821 (< 0·05%) | 7·3% | 319 (< 0·05%) | 2·2% | 2,026 (< 0·05%) | 2·5% |
| H90: Conductive and sensorineural hearing loss | 74 (< 0·05%) | 4·7% | 523 (< 0·05%) | 4·7% | 770 (< 0·05%) | 5·2% | 4,758 (< 0·05%) | 5·8% |
| H92: Otalgia and effusion of ear | 58 (< 0·05%) | 3·7% | 444 (< 0·05%) | 4·0% | 268 (< 0·05%) | 1·8% | 1,553 (< 0·05%) | 1·9% |
| H91: Other hearing loss | 45 (< 0·05%) | 2·8% | 443 (< 0·05%) | 4·0% | 9,639 (0·3%) | 65·0% | 50,921 (0·5%) | 62·1% |
| H93: Other disorders of ear, not elsewhere classified | 44 (< 0·05%) | 2·8% | 271 (< 0·05%) | 2·4% | 648 (< 0·05%) | 4·4% | 3,929 (< 0·05%) | 4·8% |
| H72: Perforation of tympanic membrane | 0 (0%) | 0·0% | 829 (< 0·05%) | 7·4% | 0 (0%) | 0·0% | 1,578 (< 0·05%) | 1·9% |
| **I00-I99 Circulatory system** | | | | | | | | |
| I25: Chronic ischaemic heart disease | 36,220 (7·3%) | 26·7% | 25,534 (0·9%) | 8·5% | 255,069 (8·3%) | 22·2% | 134,572 (1·2%) | 7·3% |
| I20: Angina pectoris | 18,675 (3·8%) | 13·7% | 12,713 (0·4%) | 4·2% | 97,101 (3·1%) | 8·5% | 86,239 (0·8%) | 4·7% |
| I50: Heart failure | 13,109 (2·6%) | 9·6% | 32,874 (1·2%) | 11·0% | 114,045 (3·7%) | 9·9% | 178,259 (1·6%) | 9·7% |
| I24: Other acute ischaemic heart diseases | 10,014 (2·0%) | 7·4% | 3,311 (0·1%) | 1·1% | 38,459 (1·2%) | 3·3% | 8,031 (0·1%) | 0·4% |
| I63: Cerebral infarction | 7,605 (1·5%) | 5·6% | 32,818 (1·2%) | 11·0% | 10,418 (0·3%) | 0·9% | 37,319 (0·3%) | 2·0% |
| I22: Subsequent myocardial infarction | 6,415 (1·3%) | 4·7% | 0 (0%) | 0·0% | 8,406 (0·3%) | 0·7% | 0 (0%) | 0·0% |
| I48: Atrial fibrillation and flutter | 4,737 (1·0%) | 3·5% | 18,537 (0·7%) | 6·2% | 67,813 (2·2%) | 5·9% | 153,087 (1·4%) | 8·3% |
| I95: Hypotension | 4,113 (0·8%) | 3·0% | 15,717 (0·6%) | 5·3% | 35,140 (1·1%) | 3·1% | 103,496 (0·9%) | 5·6% |
| I84: Haemorrhoids | 2,787 (0·6%) | 2·1% | 20,951 (0·7%) | 7·0% | 9,085 (0·3%) | 0·8% | 62,185 (0·6%) | 3·4% |
| I26: Pulmonary embolism | 2,541 (0·5%) | 1·9% | 17,376 (0·6%) | 5·8% | 5,247 (0·2%) | 0·5% | 32,749 (0·3%) | 1·8% |
| I47: Paroxysmal tachycardia | 2,248 (0·5%) | 1·7% | 5,249 (0·2%) | 1·8% | 15,342 (0·5%) | 1·3% | 16,266 (0·1%) | 0·9% |
| I49: Other cardiac arrhythmias | 1,959 (0·4%) | 1·4% | 4,913 (0·2%) | 1·6% | 14,179 (0·5%) | 1·2% | 20,375 (0·2%) | 1·1% |
| I21: Acute myocardial infarction | 1,924 (0·4%) | 1·4% | 0 (0%) | 0·0% | 2,714 (0·1%) | 0·2% | 0 (0%) | 0·0% |
| I70: Atherosclerosis | 1,622 (0·3%) | 1·2% | 5,149 (0·2%) | 1·7% | 5,791 (0·2%) | 0·5% | 14,889 (0·1%) | 0·8% |
| I77: Other disorders of arteries and arterioles | 1,541 (0·3%) | 1·1% | 5,063 (0·2%) | 1·7% | 4,406 (0·1%) | 0·4% | 11,815 (0·1%) | 0·6% |
| I74: Arterial embolism and thrombosis | 1,500 (0·3%) | 1·1% | 5,053 (0·2%) | 1·7% | 3,102 (0·1%) | 0·3% | 9,446 (0·1%) | 0·5% |
| I44: Atrioventricular and left bundle-branch block | 1,470 (0·3%) | 1·1% | 6,751 (0·2%) | 2·3% | 31,325 (1·0%) | 2·7% | 50,811 (0·5%) | 2·8% |
| I80: Phlebitis and thrombophlebitis | 1,358 (0·3%) | 1·0% | 12,611 (0·4%) | 4·2% | 3,662 (0·1%) | 0·3% | 26,985 (0·2%) | 1·5% |
| I64: Stroke, not specified as haemorrhage or infarction | 1,355 (0·3%) | 1·0% | 6,896 (0·2%) | 2·3% | 2,248 (0·1%) | 0·2% | 8,845 (0·1%) | 0·5% |
| I31: Other diseases of pericardium | 1,194 (0·2%) | 0·9% | 2,220 (0·1%) | 0·7% | 7,731 (0·3%) | 0·7% | 10,992 (0·1%) | 0·6% |
| I46: Cardiac arrest | 1,157 (0·2%) | 0·9% | 2,679 (0·1%) | 0·9% | 22,045 (0·7%) | 1·9% | 16,396 (0·1%) | 0·9% |
| I51: Complications and ill-defined descriptions of heart disease | 1,052 (0·2%) | 0·8% | 1,691 (0·1%) | 0·6% | 50,606 (1·6%) | 4·4% | 59,878 (0·5%) | 3·2% |
| I61: Intracerebral haemorrhage | 969 (0·2%) | 0·7% | 6,139 (0·2%) | 2·1% | 1,532 (< 0·05%) | 0·1% | 7,767 (0·1%) | 0·4% |
| I71: Aortic aneurysm and dissection | 930 (0·2%) | 0·7% | 3,581 (0·1%) | 1·2% | 7,932 (0·3%) | 0·7% | 20,631 (0·2%) | 1·1% |
| I83: Varicose veins of lower extremities | 879 (0·2%) | 0·6% | 9,907 (0·3%) | 3·3% | 4,067 (0·1%) | 0·4% | 24,689 (0·2%) | 1·3% |
| I73: Other peripheral vascular diseases | 777 (0·2%) | 0·6% | 3,174 (0·1%) | 1·1% | 15,567 (0·5%) | 1·4% | 38,032 (0·3%) | 2·1% |
| I42: Cardiomyopathy | 749 (0·2%) | 0·6% | 1,702 (0·1%) | 0·6% | 3,844 (0·1%) | 0·3% | 8,317 (0·1%) | 0·5% |
| I35: Nonrheumatic aortic valve disorders | 728 (0·1%) | 0·5% | 3,449 (0·1%) | 1·2% | 17,232 (0·6%) | 1·5% | 31,359 (0·3%) | 1·7% |
| I08: Multiple valve diseases | 713 (0·1%) | 0·5% | 3,361 (0·1%) | 1·1% | 30,240 (1·0%) | 2·6% | 32,969 (0·3%) | 1·8% |
| I62: Other nontraumatic intracranial haemorrhage | 624 (0·1%) | 0·5% | 3,897 (0·1%) | 1·3% | 1,026 (< 0·05%) | 0·1% | 5,914 (0·1%) | 0·3% |
| I65: Occlusion and stenosis of precerebral arteries, not resulting in cerebral infarction | 609 (0·1%) | 0·4% | 2,046 (0·1%) | 0·7% | 4,481 (0·1%) | 0·4% | 8,617 (0·1%) | 0·5% |
| I72: Other aneurysm and dissection | 489 (0·1%) | 0·4% | 1,467 (0·1%) | 0·5% | 1,992 (0·1%) | 0·2% | 4,492 (< 0·05%) | 0·2% |
| I45: Other conduction disorders | 421 (0·1%) | 0·3% | 1,843 (0·1%) | 0·6% | 15,247 (0·5%) | 1·3% | 32,434 (0·3%) | 1·8% |
| I34: Nonrheumatic mitral valve disorders | 400 (0·1%) | 0·3% | 1,383 (< 0·05%) | 0·5% | 20,177 (0·7%) | 1·8% | 20,098 (0·2%) | 1·1% |
| I23: Certain current complications following acute myocardial infarction | 396 (0·1%) | 0·3% | 0 (0%) | 0·0% | 1,284 (< 0·05%) | 0·1% | 0 (0%) | 0·0% |
| I67: Other cerebrovascular diseases | 365 (0·1%) | 0·3% | 2,391 (0·1%) | 0·8% | 14,539 (0·5%) | 1·3% | 69,268 (0·6%) | 3·8% |
| I30: Acute pericarditis | 242 (< 0·05%) | 0·2% | 0 (0%) | 0·0% | 597 (< 0·05%) | 0·1% | 0 (0%) | 0·0% |
| I33: Acute and subacute endocarditis | 232 (< 0·05%) | 0·2% | 1,288 (< 0·05%) | 0·4% | 415 (< 0·05%) | 0·0% | 1,916 (< 0·05%) | 0·1% |
| I60: Subarachnoid haemorrhage | 230 (< 0·05%) | 0·2% | 1,431 (0·1%) | 0·5% | 390 (< 0·05%) | 0·0% | 2,002 (< 0·05%) | 0·1% |
| I27: Other pulmonary heart diseases | 197 (< 0·05%) | 0·1% | 1,193 (< 0·05%) | 0·4% | 6,540 (0·2%) | 0·6% | 18,136 (0·2%) | 1·0% |
| I85: Oesophageal varices | 188 (< 0·05%) | 0·1% | 2,635 (0·1%) | 0·9% | 412 (< 0·05%) | 0·0% | 5,089 (< 0·05%) | 0·3% |
| I12: Hypertensive renal disease | 167 (< 0·05%) | 0·1% | 973 (< 0·05%) | 0·3% | 9,375 (0·3%) | 0·8% | 22,947 (0·2%) | 1·2% |
| I87: Other disorders of veins | 143 (< 0·05%) | 0·1% | 1,463 (0·1%) | 0·5% | 845 (< 0·05%) | 0·1% | 6,755 (0·1%) | 0·4% |
| I11: Hypertensive heart disease | 109 (< 0·05%) | 0·1% | 351 (< 0·05%) | 0·1% | 694 (< 0·05%) | 0·1% | 1,718 (< 0·05%) | 0·1% |
| I78: Diseases of capillaries | 109 (< 0·05%) | 0·1% | 856 (< 0·05%) | 0·3% | 539 (< 0·05%) | 0·0% | 4,035 (< 0·05%) | 0·2% |
| I99: Other and unspecified disorders of circulatory system | 104 (< 0·05%) | 0·1% | 410 (< 0·05%) | 0·1% | 385 (< 0·05%) | 0·0% | 1,505 (< 0·05%) | 0·1% |
| I10: Essential (primary) hypertension | 104 (< 0·05%) | 0·1% | 797 (< 0·05%) | 0·3% | 169,769 (5·5%) | 14·8% | 367,869 (3·3%) | 19·9% |
| I89: Other noninfective disorders of lymphatic vessels and lymph nodes | 90 (< 0·05%) | 0·1% | 992 (< 0·05%) | 0·3% | 889 (< 0·05%) | 0·1% | 7,682 (0·1%) | 0·4% |
| I82: Other venous embolism and thrombosis | 88 (< 0·05%) | 0·1% | 807 (< 0·05%) | 0·3% | 493 (< 0·05%) | 0·0% | 3,314 (< 0·05%) | 0·2% |
| I05: Rheumatic mitral valve diseases | 67 (< 0·05%) | 0·0% | 315 (< 0·05%) | 0·1% | 1,027 (< 0·05%) | 0·1% | 2,632 (< 0·05%) | 0·1% |
| I38: Endocarditis, valve unspecified | 50 (< 0·05%) | 0·0% | 284 (< 0·05%) | 0·1% | 1,018 (< 0·05%) | 0·1% | 4,315 (< 0·05%) | 0·2% |
| I97: Postprocedural disorders of circulatory system, not elsewhere classified | 49 (< 0·05%) | 0·0% | 0 (0%) | 0·0% | 499 (< 0·05%) | 0·0% | 0 (0%) | 0·0% |
| I07: Rheumatic tricuspid valve diseases | 24 (< 0·05%) | 0·0% | 158 (< 0·05%) | 0·1% | 4,298 (0·1%) | 0·4% | 6,398 (0·1%) | 0·3% |
| I79: Disorders of arteries, arterioles and capillaries in diseases classified elsewhere | 17 (< 0·05%) | 0·0% | 70 (< 0·05%) | 0·0% | 340 (< 0·05%) | 0·0% | 1,578 (< 0·05%) | 0·1% |
| I36: Nonrheumatic tricuspid valve disorders | 11 (< 0·05%) | 0·0% | 64 (< 0·05%) | 0·0% | 1,193 (< 0·05%) | 0·1% | 2,133 (< 0·05%) | 0·1% |
| I15: Secondary hypertension | < 10 (< 0·05%) | 0·0% | 39 (< 0·05%) | 0·0% | 445 (< 0·05%) | 0·0% | 1,699 (< 0·05%) | 0·1% |
| I69: Sequelae of cerebrovascular disease | < 10 (< 0·05%) | 0·0% | 24 (< 0·05%) | 0·0% | 4,153 (0·1%) | 0·4% | 21,338 (0·2%) | 1·2% |
| I37: Pulmonary valve disorders | < 10 (< 0·05%) | 0·0% | 46 (< 0·05%) | 0·0% | 1,625 (0·1%) | 0·1% | 2,064 (< 0·05%) | 0·1% |
| I98: Other disorders of circulatory system in diseases classified elsewhere | 0 (0%) | 0·0% | 1,489 (0·1%) | 0·5% | 0 (0%) | 0·0% | 7,647 (0·1%) | 0·4% |
| I86: Varicose veins of other sites | 0 (0%) | 0·0% | 857 (< 0·05%) | 0·3% | 0 (0%) | 0·0% | 3,537 (< 0·05%) | 0·2% |
| I81: Portal vein thrombosis | 0 (0%) | 0·0% | 185 (< 0·05%) | 0·1% | 0 (0%) | 0·0% | 2,318 (< 0·05%) | 0·1% |
| **J00-J99 Respiratory system** | | | | | | | | |
| J18: Pneumonia, organism unspecified | 24,623 (5·0%) | 41·9% | 143,663 (5·1%) | 40·8% | 56,467 (1·8%) | 18·0% | 234,587 (2·1%) | 17·5% |
| J44: Other chronic obstructive pulmonary disease | 10,220 (2·1%) | 17·4% | 52,304 (1·8%) | 14·8% | 49,806 (1·6%) | 15·9% | 197,897 (1·8%) | 14·8% |
| J22: Unspecified acute lower respiratory infection | 9,710 (2·0%) | 16·5% | 55,121 (1·9%) | 15·6% | 30,701 (1·0%) | 9·8% | 118,453 (1·1%) | 8·8% |
| J90: Pleural effusion, not elsewhere classified | 2,700 (0·5%) | 4·6% | 12,341 (0·4%) | 3·5% | 34,208 (1·1%) | 10·9% | 105,900 (0·9%) | 7·9% |
| J69: Pneumonitis due to solids and liquids | 1,926 (0·4%) | 3·3% | 17,102 (0·6%) | 4·9% | 6,668 (0·2%) | 2·1% | 33,061 (0·3%) | 2·5% |
| J45: Asthma | 1,138 (0·2%) | 1·9% | 8,952 (0·3%) | 2·5% | 60,584 (2·0%) | 19·3% | 311,387 (2·8%) | 23·2% |
| J96: Respiratory failure, not elsewhere classified | 998 (0·2%) | 1·7% | 6,848 (0·2%) | 1·9% | 18,936 (0·6%) | 6·0% | 69,902 (0·6%) | 5·2% |
| J98: Other respiratory disorders | 668 (0·1%) | 1·1% | 4,783 (0·2%) | 1·4% | 12,746 (0·4%) | 4·1% | 53,610 (0·5%) | 4·0% |
| J81: Pulmonary oedema | 626 (0·1%) | 1·1% | 1,372 (< 0·05%) | 0·4% | 5,472 (0·2%) | 1·7% | 8,905 (0·1%) | 0·7% |
| J47: Bronchiectasis | 601 (0·1%) | 1·0% | 6,307 (0·2%) | 1·8% | 6,601 (0·2%) | 2·1% | 43,922 (0·4%) | 3·3% |
| J38: Diseases of vocal cords and larynx, not elsewhere classified | 566 (0·1%) | 1·0% | 5,192 (0·2%) | 1·5% | 1,017 (< 0·05%) | 0·3% | 8,283 (0·1%) | 0·6% |
| J15: Bacterial pneumonia, not elsewhere classified | 564 (0·1%) | 1·0% | 3,844 (0·1%) | 1·1% | 1,434 (< 0·05%) | 0·5% | 6,314 (0·1%) | 0·5% |
| J34: Other disorders of nose and nasal sinuses | 477 (0·1%) | 0·8% | 5,213 (0·2%) | 1·5% | 894 (< 0·05%) | 0·3% | 8,780 (0·1%) | 0·7% |
| J06: Acute upper respiratory infections of multiple and unspecified sites | 443 (0·1%) | 0·8% | 2,243 (0·1%) | 0·6% | 896 (< 0·05%) | 0·3% | 4,166 (< 0·05%) | 0·3% |
| J84: Other interstitial pulmonary diseases | 409 (0·1%) | 0·7% | 2,487 (0·1%) | 0·7% | 3,861 (0·1%) | 1·2% | 16,178 (0·1%) | 1·2% |
| J93: Pneumothorax | 348 (0·1%) | 0·6% | 2,380 (0·1%) | 0·7% | 2,122 (0·1%) | 0·7% | 8,933 (0·1%) | 0·7% |
| J33: Nasal polyp | 279 (0·1%) | 0·5% | 2,964 (0·1%) | 0·8% | 525 (< 0·05%) | 0·2% | 5,051 (< 0·05%) | 0·4% |
| J43: Emphysema | 253 (0·1%) | 0·4% | 1,449 (0·1%) | 0·4% | 7,079 (0·2%) | 2·3% | 29,687 (0·3%) | 2·2% |
| J40: Bronchitis, not specified as acute or chronic | 247 (< 0·05%) | 0·4% | 1,425 (0·1%) | 0·4% | 998 (< 0·05%) | 0·3% | 4,841 (< 0·05%) | 0·4% |
| J20: Acute bronchitis | 228 (< 0·05%) | 0·4% | 1,165 (< 0·05%) | 0·3% | 312 (< 0·05%) | 0·1% | 1,582 (< 0·05%) | 0·1% |
| J13: Pneumonia due to Streptococcus pneumoniae | 213 (< 0·05%) | 0·4% | 1,722 (0·1%) | 0·5% | 421 (< 0·05%) | 0·1% | 2,135 (< 0·05%) | 0·2% |
| J10: Influenza due to other identified influenza virus | 207 (< 0·05%) | 0·4% | 1,526 (0·1%) | 0·4% | 384 (< 0·05%) | 0·1% | 2,371 (< 0·05%) | 0·2% |
| J86: Pyothorax | 198 (< 0·05%) | 0·3% | 1,763 (0·1%) | 0·5% | 489 (< 0·05%) | 0·2% | 3,828 (< 0·05%) | 0·3% |
| J39: Other diseases of upper respiratory tract | 174 (< 0·05%) | 0·3% | 1,830 (0·1%) | 0·5% | 484 (< 0·05%) | 0·2% | 4,006 (< 0·05%) | 0·3% |
| J32: Chronic sinusitis | 165 (< 0·05%) | 0·3% | 1,542 (0·1%) | 0·4% | 620 (< 0·05%) | 0·2% | 4,999 (< 0·05%) | 0·4% |
| J14: Pneumonia due to Haemophilus influenzae | 147 (< 0·05%) | 0·3% | 831 (< 0·05%) | 0·2% | 388 (< 0·05%) | 0·1% | 1,271 (< 0·05%) | 0·1% |
| J95: Postprocedural respiratory disorders, not elsewhere classified | 134 (< 0·05%) | 0·2% | 878 (< 0·05%) | 0·2% | 989 (< 0·05%) | 0·3% | 3,351 (< 0·05%) | 0·2% |
| J02: Acute pharyngitis | 132 (< 0·05%) | 0·2% | 1,023 (< 0·05%) | 0·3% | 518 (< 0·05%) | 0·2% | 3,373 (< 0·05%) | 0·3% |
| J94: Other pleural conditions | 130 (< 0·05%) | 0·2% | 780 (< 0·05%) | 0·2% | 845 (< 0·05%) | 0·3% | 4,319 (< 0·05%) | 0·3% |
| J92: Pleural plaque | 77 (< 0·05%) | 0·1% | 420 (< 0·05%) | 0·1% | 4,052 (0·1%) | 1·3% | 17,085 (0·2%) | 1·3% |
| J91: Pleural effusion in conditions classified elsewhere | 36 (< 0·05%) | 0·1% | 314 (< 0·05%) | 0·1% | 495 (< 0·05%) | 0·2% | 2,449 (< 0·05%) | 0·2% |
| J80: Adult respiratory distress syndrome | 32 (< 0·05%) | 0·1% | 167 (< 0·05%) | 0·0% | 610 (< 0·05%) | 0·2% | 1,741 (< 0·05%) | 0·1% |
| J42: Unspecified chronic bronchitis | 19 (< 0·05%) | 0·0% | 133 (< 0·05%) | 0·0% | 394 (< 0·05%) | 0·1% | 1,930 (< 0·05%) | 0·1% |
| J61: Pneumoconiosis due to asbestos and other mineral fibres | 16 (< 0·05%) | 0·0% | 76 (< 0·05%) | 0·0% | 863 (< 0·05%) | 0·3% | 3,467 (< 0·05%) | 0·3% |
| J30: Vasomotor and allergic rhinitis | < 10 (< 0·05%) | 0·0% | 62 (< 0·05%) | 0·0% | 593 (< 0·05%) | 0·2% | 3,954 (< 0·05%) | 0·3% |
| J35: Chronic diseases of tonsils and adenoids | 0 (0%) | 0·0% | 818 (< 0·05%) | 0·2% | 0 (0%) | 0·0% | 1,364 (< 0·05%) | 0·1% |
| J03: Acute tonsillitis | 0 (0%) | 0·0% | 775 (< 0·05%) | 0·2% | 0 (0%) | 0·0% | 1,083 (< 0·05%) | 0·1% |
| J31: Chronic rhinitis, nasopharyngitis and pharyngitis | 0 (0%) | 0·0% | 310 (< 0·05%) | 0·1% | 0 (0%) | 0·0% | 1,642 (< 0·05%) | 0·1% |
| J00: Acute nasopharyngitis [common cold] | 0 (0%) | 0·0% | 187 (< 0·05%) | 0·1% | 0 (0%) | 0·0% | 1,332 (< 0·05%) | 0·1% |
| J17: Pneumonia in diseases classified elsewhere | 0 (0%) | 0·0% | 179 (< 0·05%) | 0·1% | 0 (0%) | 0·0% | 1,699 (< 0·05%) | 0·1% |
| J99: Respiratory disorders in diseases classified elsewhere | 0 (0%) | 0·0% | 23 (< 0·05%) | 0·0% | 0 (0%) | 0·0% | 1,900 (< 0·05%) | 0·1% |
| **K00-K93 Digestive system** | | | | | | | | |
| K29: Gastritis and duodenitis | 9,215 (1·9%) | 9·6% | 45,801 (1·6%) | 7·8% | 23,328 (0·8%) | 8·1% | 124,555 (1·1%) | 7·5% |
| K92: Other diseases of digestive system | 8,688 (1·7%) | 9·0% | 43,963 (1·6%) | 7·5% | 21,011 (0·7%) | 7·3% | 88,361 (0·8%) | 5·3% |
| K57: Diverticular disease of intestine | 6,401 (1·3%) | 6·7% | 36,315 (1·3%) | 6·2% | 21,914 (0·7%) | 7·6% | 123,350 (1·1%) | 7·5% |
| K80: Cholelithiasis | 6,068 (1·2%) | 6·3% | 36,577 (1·3%) | 6·2% | 12,795 (0·4%) | 4·4% | 68,973 (0·6%) | 4·2% |
| K63: Other diseases of intestine | 5,573 (1·1%) | 5·8% | 40,904 (1·4%) | 6·9% | 10,436 (0·3%) | 3·6% | 76,498 (0·7%) | 4·6% |
| K40: Inguinal hernia | 4,985 (1·0%) | 5·2% | 31,980 (1·1%) | 5·4% | 7,172 (0·2%) | 2·5% | 43,591 (0·4%) | 2·6% |
| K21: Gastro-oesophageal reflux disease | 4,810 (1·0%) | 5·0% | 20,591 (0·7%) | 3·5% | 22,133 (0·7%) | 7·6% | 92,130 (0·8%) | 5·6% |
| K62: Other diseases of anus and rectum | 4,776 (1·0%) | 5·0% | 33,959 (1·2%) | 5·8% | 9,819 (0·3%) | 3·4% | 66,896 (0·6%) | 4·0% |
| K59: Other functional intestinal disorders | 3,687 (0·7%) | 3·8% | 26,931 (0·9%) | 4·6% | 23,310 (0·8%) | 8·1% | 139,710 (1·3%) | 8·4% |
| K22: Other diseases of oesophagus | 3,597 (0·7%) | 3·7% | 23,315 (0·8%) | 4·0% | 8,155 (0·3%) | 2·8% | 49,390 (0·4%) | 3·0% |
| K52: Other noninfective gastroenteritis and colitis | 3,573 (0·7%) | 3·7% | 22,561 (0·8%) | 3·8% | 12,828 (0·4%) | 4·4% | 58,717 (0·5%) | 3·5% |
| K44: Diaphragmatic hernia | 3,370 (0·7%) | 3·5% | 21,504 (0·8%) | 3·6% | 31,515 (1·0%) | 10·9% | 189,933 (1·7%) | 11·5% |
| K30: Functional dyspepsia | 2,871 (0·6%) | 3·0% | 12,383 (0·4%) | 2·1% | 5,721 (0·2%) | 2·0% | 24,068 (0·2%) | 1·5% |
| K02: Dental caries | 2,183 (0·4%) | 2·3% | 9,880 (0·3%) | 1·7% | 2,820 (0·1%) | 1·0% | 12,873 (0·1%) | 0·8% |
| K25: Gastric ulcer | 1,986 (0·4%) | 2·1% | 7,451 (0·3%) | 1·3% | 5,020 (0·2%) | 1·7% | 20,210 (0·2%) | 1·2% |
| K56: Paralytic ileus and intestinal obstruction without hernia | 1,825 (0·4%) | 1·9% | 18,358 (0·6%) | 3·1% | 4,720 (0·2%) | 1·6% | 37,324 (0·3%) | 2·3% |
| K20: Oesophagitis | 1,790 (0·4%) | 1·9% | 12,705 (0·4%) | 2·2% | 5,073 (0·2%) | 1·8% | 34,314 (0·3%) | 2·1% |
| K31: Other diseases of stomach and duodenum | 1,784 (0·4%) | 1·9% | 14,100 (0·5%) | 2·4% | 5,846 (0·2%) | 2·0% | 47,807 (0·4%) | 2·9% |
| K85: Acute pancreatitis | 1,622 (0·3%) | 1·7% | 10,220 (0·4%) | 1·7% | 2,414 (0·1%) | 0·8% | 14,754 (0·1%) | 0·9% |
| K26: Duodenal ulcer | 1,567 (0·3%) | 1·6% | 5,998 (0·2%) | 1·0% | 4,281 (0·1%) | 1·5% | 16,871 (0·2%) | 1·0% |
| K81: Cholecystitis | 1,378 (0·3%) | 1·4% | 7,433 (0·3%) | 1·3% | 2,068 (0·1%) | 0·7% | 10,621 (0·1%) | 0·6% |
| K08: Other disorders of teeth and supporting structures | 1,246 (0·3%) | 1·3% | 5,882 (0·2%) | 1·0% | 2,080 (0·1%) | 0·7% | 9,298 (0·1%) | 0·6% |
| K83: Other diseases of biliary tract | 1,166 (0·2%) | 1·2% | 7,988 (0·3%) | 1·4% | 2,722 (0·1%) | 0·9% | 17,631 (0·2%) | 1·1% |
| K43: Ventral hernia | 1,086 (0·2%) | 1·1% | 10,820 (0·4%) | 1·8% | 2,257 (0·1%) | 0·8% | 20,733 (0·2%) | 1·3% |
| K55: Vascular disorders of intestine | 1,035 (0·2%) | 1·1% | 4,438 (0·2%) | 0·8% | 2,656 (0·1%) | 0·9% | 11,453 (0·1%) | 0·7% |
| K60: Fissure and fistula of anal and rectal regions | 954 (0·2%) | 1·0% | 7,016 (0·2%) | 1·2% | 1,482 (< 0·05%) | 0·5% | 10,479 (0·1%) | 0·6% |
| K42: Umbilical hernia | 921 (0·2%) | 1·0% | 7,460 (0·3%) | 1·3% | 2,291 (0·1%) | 0·8% | 17,062 (0·2%) | 1·0% |
| K91: Postprocedural disorders of digestive system, not elsewhere classified | 602 (0·1%) | 0·6% | 7,806 (0·3%) | 1·3% | 1,505 (< 0·05%) | 0·5% | 15,919 (0·1%) | 1·0% |
| K61: Abscess of anal and rectal regions | 578 (0·1%) | 0·6% | 3,386 (0·1%) | 0·6% | 744 (< 0·05%) | 0·3% | 4,219 (< 0·05%) | 0·3% |
| K13: Other diseases of lip and oral mucosa | 577 (0·1%) | 0·6% | 4,458 (0·2%) | 0·8% | 911 (< 0·05%) | 0·3% | 6,728 (0·1%) | 0·4% |
| K35: Acute appendicitis | 565 (0·1%) | 0·6% | 3,056 (0·1%) | 0·5% | 616 (< 0·05%) | 0·2% | 3,324 (< 0·05%) | 0·2% |
| K64: Haemorrhoids and perianal venous thrombosis | 550 (0·1%) | 0·6% | 4,070 (0·1%) | 0·7% | 1,887 (0·1%) | 0·7% | 12,672 (0·1%) | 0·8% |
| K51: Ulcerative colitis | 520 (0·1%) | 0·5% | 3,494 (0·1%) | 0·6% | 1,655 (0·1%) | 0·6% | 8,922 (0·1%) | 0·5% |
| K04: Diseases of pulp and periapical tissues | 505 (0·1%) | 0·5% | 2,410 (0·1%) | 0·4% | 977 (< 0·05%) | 0·3% | 4,368 (< 0·05%) | 0·3% |
| K86: Other diseases of pancreas | 385 (0·1%) | 0·4% | 3,701 (0·1%) | 0·6% | 1,741 (0·1%) | 0·6% | 14,300 (0·1%) | 0·9% |
| K14: Diseases of tongue | 344 (0·1%) | 0·4% | 2,375 (0·1%) | 0·4% | 641 (< 0·05%) | 0·2% | 4,073 (< 0·05%) | 0·2% |
| K65: Peritonitis | 337 (0·1%) | 0·4% | 2,990 (0·1%) | 0·5% | 1,000 (< 0·05%) | 0·3% | 8,447 (0·1%) | 0·5% |
| K05: Gingivitis and periodontal diseases | 287 (0·1%) | 0·3% | 1,298 (< 0·05%) | 0·2% | 604 (< 0·05%) | 0·2% | 2,575 (< 0·05%) | 0·2% |
| K76: Other diseases of liver | 271 (0·1%) | 0·3% | 2,331 (0·1%) | 0·4% | 4,756 (0·2%) | 1·6% | 32,253 (0·3%) | 1·9% |
| K41: Femoral hernia | 259 (0·1%) | 0·3% | 1,455 (0·1%) | 0·2% | 375 (< 0·05%) | 0·1% | 2,102 (< 0·05%) | 0·1% |
| K75: Other inflammatory liver diseases | 233 (< 0·05%) | 0·2% | 1,633 (0·1%) | 0·3% | 952 (< 0·05%) | 0·3% | 5,292 (< 0·05%) | 0·3% |
| K11: Diseases of salivary glands | 203 (< 0·05%) | 0·2% | 1,700 (0·1%) | 0·3% | 379 (< 0·05%) | 0·1% | 2,978 (< 0·05%) | 0·2% |
| K50: Crohn's disease [regional enteritis] | 194 (< 0·05%) | 0·2% | 1,829 (0·1%) | 0·3% | 972 (< 0·05%) | 0·3% | 5,529 (< 0·05%) | 0·3% |
| K10: Other diseases of jaws | 185 (< 0·05%) | 0·2% | 953 (< 0·05%) | 0·2% | 426 (< 0·05%) | 0·1% | 1,850 (< 0·05%) | 0·1% |
| K70: Alcoholic liver disease | 171 (< 0·05%) | 0·2% | 2,399 (0·1%) | 0·4% | 1,045 (< 0·05%) | 0·4% | 10,293 (0·1%) | 0·6% |
| K82: Other diseases of gallbladder | 167 (< 0·05%) | 0·2% | 1,122 (< 0·05%) | 0·2% | 903 (< 0·05%) | 0·3% | 6,548 (0·1%) | 0·4% |
| K12: Stomatitis and related lesions | 164 (< 0·05%) | 0·2% | 1,346 (< 0·05%) | 0·2% | 717 (< 0·05%) | 0·2% | 6,497 (0·1%) | 0·4% |
| K90: Intestinal malabsorption | 162 (< 0·05%) | 0·2% | 860 (< 0·05%) | 0·1% | 973 (< 0·05%) | 0·3% | 4,714 (< 0·05%) | 0·3% |
| K66: Other disorders of peritoneum | 149 (< 0·05%) | 0·2% | 1,719 (0·1%) | 0·3% | 1,997 (0·1%) | 0·7% | 19,896 (0·2%) | 1·2% |
| K72: Hepatic failure, not elsewhere classified | 148 (< 0·05%) | 0·2% | 1,714 (0·1%) | 0·3% | 1,898 (0·1%) | 0·7% | 7,839 (0·1%) | 0·5% |
| K01: Embedded and impacted teeth | 147 (< 0·05%) | 0·2% | 917 (< 0·05%) | 0·2% | 210 (< 0·05%) | 0·1% | 1,270 (< 0·05%) | 0·1% |
| K74: Fibrosis and cirrhosis of liver | 120 (< 0·05%) | 0·1% | 1,403 (< 0·05%) | 0·2% | 1,011 (< 0·05%) | 0·3% | 9,674 (0·1%) | 0·6% |
| K58: Irritable bowel syndrome | 114 (< 0·05%) | 0·1% | 906 (< 0·05%) | 0·2% | 3,233 (0·1%) | 1·1% | 16,209 (0·1%) | 1·0% |
| K27: Peptic ulcer, site unspecified | 85 (< 0·05%) | 0·1% | 361 (< 0·05%) | 0·1% | 912 (< 0·05%) | 0·3% | 3,032 (< 0·05%) | 0·2% |
| K46: Unspecified abdominal hernia | 40 (< 0·05%) | 0·0% | 397 (< 0·05%) | 0·1% | 482 (< 0·05%) | 0·2% | 3,013 (< 0·05%) | 0·2% |
| K06: Other disorders of gingiva and edentulous alveolar ridge | 0 (0%) | 0·0% | 663 (< 0·05%) | 0·1% | 0 (0%) | 0·0% | 1,187 (< 0·05%) | 0·1% |
| K07: Dentofacial anomalies [including malocclusion] | 0 (0%) | 0·0% | 646 (< 0·05%) | 0·1% | 0 (0%) | 0·0% | 1,053 (< 0·05%) | 0·1% |
| **L00-L99 Skin and subcutaneous tissue** | | | | | | | | |
| L03: Cellulitis | 4,467 (0·9%) | 39·9% | 32,354 (1·1%) | 35·0% | 12,904 (0·4%) | 24·8% | 77,445 (0·7%) | 24·5% |
| L98: Other disorders of skin and subcutaneous tissue, not elsewhere classified | 1,395 (0·3%) | 12·5% | 12,252 (0·4%) | 13·2% | 3,182 (0·1%) | 6·1% | 23,386 (0·2%) | 7·4% |
| L72: Follicular cysts of skin and subcutaneous tissue | 933 (0·2%) | 8·3% | 7,158 (0·3%) | 7·7% | 1,159 (< 0·05%) | 2·2% | 8,960 (0·1%) | 2·8% |
| L02: Cutaneous abscess, furuncle and carbuncle | 925 (0·2%) | 8·3% | 6,608 (0·2%) | 7·1% | 1,401 (< 0·05%) | 2·7% | 9,847 (0·1%) | 3·1% |
| L57: Skin changes due to chronic exposure to nonionizing radiation | 805 (0·2%) | 7·2% | 7,979 (0·3%) | 8·6% | 1,600 (0·1%) | 3·1% | 16,113 (0·1%) | 5·1% |
| L97: Ulcer of lower limb, not elsewhere classified | 658 (0·1%) | 5·9% | 3,861 (0·1%) | 4·2% | 7,888 (0·3%) | 15·2% | 36,414 (0·3%) | 11·5% |
| L82: Seborrhoeic keratosis | 469 (0·1%) | 4·2% | 4,098 (0·1%) | 4·4% | 762 (< 0·05%) | 1·5% | 7,334 (0·1%) | 2·3% |
| L90: Atrophic disorders of skin | 289 (0·1%) | 2·6% | 3,572 (0·1%) | 3·9% | 547 (< 0·05%) | 1·1% | 6,430 (0·1%) | 2·0% |
| L08: Other local infections of skin and subcutaneous tissue | 215 (< 0·05%) | 1·9% | 1,896 (0·1%) | 2·0% | 2,716 (0·1%) | 5·2% | 17,751 (0·2%) | 5·6% |
| L85: Other epidermal thickening | 214 (< 0·05%) | 1·9% | 1,757 (0·1%) | 1·9% | 337 (< 0·05%) | 0·6% | 3,121 (< 0·05%) | 1·0% |
| L60: Nail disorders | 155 (< 0·05%) | 1·4% | 1,341 (< 0·05%) | 1·4% | 298 (< 0·05%) | 0·6% | 2,201 (< 0·05%) | 0·7% |
| L30: Other dermatitis | 154 (< 0·05%) | 1·4% | 1,239 (< 0·05%) | 1·3% | 2,259 (0·1%) | 4·3% | 13,294 (0·1%) | 4·2% |
| L89: Decubitus ulcer and pressure area | 153 (< 0·05%) | 1·4% | 1,117 (< 0·05%) | 1·2% | 11,354 (0·4%) | 21·8% | 60,067 (0·5%) | 19·0% |
| L27: Dermatitis due to substances taken internally | 120 (< 0·05%) | 1·1% | 736 (< 0·05%) | 0·8% | 756 (< 0·05%) | 1·5% | 3,186 (< 0·05%) | 1·0% |
| L40: Psoriasis | 73 (< 0·05%) | 0·7% | 492 (< 0·05%) | 0·5% | 3,034 (0·1%) | 5·8% | 10,969 (0·1%) | 3·5% |
| L53: Other erythematous conditions | 70 (< 0·05%) | 0·6% | 626 (< 0·05%) | 0·7% | 871 (< 0·05%) | 1·7% | 5,187 (< 0·05%) | 1·6% |
| L50: Urticaria | 65 (< 0·05%) | 0·6% | 338 (< 0·05%) | 0·4% | 378 (< 0·05%) | 0·7% | 1,408 (< 0·05%) | 0·4% |
| L29: Pruritus | 43 (< 0·05%) | 0·4% | 387 (< 0·05%) | 0·4% | 591 (< 0·05%) | 1·1% | 3,353 (< 0·05%) | 1·1% |
| L81: Other disorders of pigmentation | 0 (0%) | 0·0% | 915 (< 0·05%) | 1·0% | 0 (0%) | 0·0% | 1,806 (< 0·05%) | 0·6% |
| L05: Pilonidal cyst | 0 (0%) | 0·0% | 790 (< 0·05%) | 0·9% | 0 (0%) | 0·0% | 1,019 (< 0·05%) | 0·3% |
| L92: Granulomatous disorders of skin and subcutaneous tissue | 0 (0%) | 0·0% | 772 (< 0·05%) | 0·8% | 0 (0%) | 0·0% | 1,553 (< 0·05%) | 0·5% |
| L91: Hypertrophic disorders of skin | 0 (0%) | 0·0% | 708 (< 0·05%) | 0·8% | 0 (0%) | 0·0% | 1,456 (< 0·05%) | 0·5% |
| L73: Other follicular disorders | 0 (0%) | 0·0% | 646 (< 0·05%) | 0·7% | 0 (0%) | 0·0% | 1,555 (< 0·05%) | 0·5% |
| L43: Lichen planus | 0 (0%) | 0·0% | 467 (< 0·05%) | 0·5% | 0 (0%) | 0·0% | 1,224 (< 0·05%) | 0·4% |
| L12: Pemphigoid | 0 (0%) | 0·0% | 388 (< 0·05%) | 0·4% | 0 (0%) | 0·0% | 1,358 (< 0·05%) | 0·4% |
| **M00-M99 Musculoskeletal system and connective tissue** | | | | | | | | |
| M54: Dorsalgia | 7,238 (1·5%) | 16·6% | 70,870 (2·5%) | 18·7% | 19,036 (0·6%) | 10·2% | 130,746 (1·2%) | 11·3% |
| M79: Other soft tissue disorders, not elsewhere classified | 5,471 (1·1%) | 12·6% | 35,533 (1·3%) | 9·4% | 13,981 (0·5%) | 7·5% | 81,622 (0·7%) | 7·1% |
| M25: Other joint disorders, not elsewhere classified | 4,730 (1·0%) | 10·9% | 37,674 (1·3%) | 10·0% | 11,582 (0·4%) | 6·2% | 80,620 (0·7%) | 7·0% |
| M16: Coxarthrosis [arthrosis of hip] | 3,171 (0·6%) | 7·3% | 22,298 (0·8%) | 5·9% | 5,932 (0·2%) | 3·2% | 36,862 (0·3%) | 3·2% |
| M17: Gonarthrosis [arthrosis of knee] | 3,128 (0·6%) | 7·2% | 27,501 (1·0%) | 7·3% | 9,358 (0·3%) | 5·0% | 64,939 (0·6%) | 5·6% |
| M75: Shoulder lesions | 2,190 (0·4%) | 5·0% | 20,014 (0·7%) | 5·3% | 3,418 (0·1%) | 1·8% | 27,092 (0·2%) | 2·4% |
| M51: Other intervertebral disc disorders | 2,043 (0·4%) | 4·7% | 19,620 (0·7%) | 5·2% | 4,408 (0·1%) | 2·4% | 37,413 (0·3%) | 3·2% |
| M48: Other spondylopathies | 1,747 (0·4%) | 4·0% | 16,832 (0·6%) | 4·4% | 6,166 (0·2%) | 3·3% | 49,883 (0·4%) | 4·3% |
| M23: Internal derangement of knee | 1,475 (0·3%) | 3·4% | 14,296 (0·5%) | 3·8% | 1,698 (0·1%) | 0·9% | 16,854 (0·2%) | 1·5% |
| M72: Fibroblastic disorders | 1,059 (0·2%) | 2·4% | 9,980 (0·4%) | 2·6% | 1,361 (< 0·05%) | 0·7% | 12,199 (0·1%) | 1·1% |
| M47: Spondylosis | 990 (0·2%) | 2·3% | 8,519 (0·3%) | 2·3% | 7,699 (0·2%) | 4·1% | 48,136 (0·4%) | 4·2% |
| M19: Other arthrosis | 985 (0·2%) | 2·3% | 9,066 (0·3%) | 2·4% | 24,983 (0·8%) | 13·4% | 121,535 (1·1%) | 10·5% |
| M65: Synovitis and tenosynovitis | 662 (0·1%) | 1·5% | 6,039 (0·2%) | 1·6% | 1,182 (< 0·05%) | 0·6% | 12,203 (0·1%) | 1·1% |
| M15: Polyarthrosis | 612 (0·1%) | 1·4% | 5,633 (0·2%) | 1·5% | 4,017 (0·1%) | 2·2% | 25,805 (0·2%) | 2·2% |
| M80: Osteoporosis with pathological fracture | 572 (0·1%) | 1·3% | 4,600 (0·2%) | 1·2% | 1,870 (0·1%) | 1·0% | 12,706 (0·1%) | 1·1% |
| M20: Acquired deformities of fingers and toes | 558 (0·1%) | 1·3% | 6,789 (0·2%) | 1·8% | 752 (< 0·05%) | 0·4% | 8,459 (0·1%) | 0·7% |
| M94: Other disorders of cartilage | 532 (0·1%) | 1·2% | 1,270 (< 0·05%) | 0·3% | 788 (< 0·05%) | 0·4% | 2,361 (< 0·05%) | 0·2% |
| M10: Gout | 496 (0·1%) | 1·1% | 2,634 (0·1%) | 0·7% | 8,895 (0·3%) | 4·8% | 34,564 (0·3%) | 3·0% |
| M70: Soft tissue disorders related to use, overuse and pressure | 493 (0·1%) | 1·1% | 3,859 (0·1%) | 1·0% | 823 (< 0·05%) | 0·4% | 6,333 (0·1%) | 0·5% |
| M86: Osteomyelitis | 428 (0·1%) | 1·0% | 2,801 (0·1%) | 0·7% | 1,000 (< 0·05%) | 0·5% | 6,390 (0·1%) | 0·6% |
| M13: Other arthritis | 395 (0·1%) | 0·9% | 3,514 (0·1%) | 0·9% | 17,058 (0·6%) | 9·2% | 85,773 (0·8%) | 7·4% |
| M84: Disorders of continuity of bone | 389 (0·1%) | 0·9% | 4,386 (0·2%) | 1·2% | 829 (< 0·05%) | 0·4% | 7,729 (0·1%) | 0·7% |
| M43: Other deforming dorsopathies | 366 (0·1%) | 0·8% | 3,517 (0·1%) | 0·9% | 1,696 (0·1%) | 0·9% | 14,230 (0·1%) | 1·2% |
| M50: Cervical disc disorders | 336 (0·1%) | 0·8% | 3,264 (0·1%) | 0·9% | 855 (< 0·05%) | 0·5% | 7,283 (0·1%) | 0·6% |
| M81: Osteoporosis without pathological fracture | 302 (0·1%) | 0·7% | 2,898 (0·1%) | 0·8% | 10,714 (0·3%) | 5·8% | 60,885 (0·5%) | 5·3% |
| M00: Pyogenic arthritis | 277 (0·1%) | 0·6% | 2,087 (0·1%) | 0·6% | 439 (< 0·05%) | 0·2% | 3,202 (< 0·05%) | 0·3% |
| M67: Other disorders of synovium and tendon | 266 (0·1%) | 0·6% | 2,791 (0·1%) | 0·7% | 434 (< 0·05%) | 0·2% | 4,695 (< 0·05%) | 0·4% |
| M24: Other specific joint derangements | 236 (< 0·05%) | 0·5% | 2,609 (0·1%) | 0·7% | 657 (< 0·05%) | 0·4% | 6,827 (0·1%) | 0·6% |
| M62: Other disorders of muscle | 235 (< 0·05%) | 0·5% | 1,619 (0·1%) | 0·4% | 1,352 (< 0·05%) | 0·7% | 6,904 (0·1%) | 0·6% |
| M96: Postprocedural musculoskeletal disorders, not elsewhere classified | 218 (< 0·05%) | 0·5% | 1,990 (0·1%) | 0·5% | 290 (< 0·05%) | 0·2% | 2,696 (< 0·05%) | 0·2% |
| M18: Arthrosis of first carpometacarpal joint | 189 (< 0·05%) | 0·4% | 1,990 (0·1%) | 0·5% | 355 (< 0·05%) | 0·2% | 3,040 (< 0·05%) | 0·3% |
| M06: Other rheumatoid arthritis | 180 (< 0·05%) | 0·4% | 1,683 (0·1%) | 0·4% | 6,905 (0·2%) | 3·7% | 26,415 (0·2%) | 2·3% |
| M46: Other inflammatory spondylopathies | 171 (< 0·05%) | 0·4% | 1,853 (0·1%) | 0·5% | 1,452 (< 0·05%) | 0·8% | 8,952 (0·1%) | 0·8% |
| M31: Other necrotizing vasculopathies | 166 (< 0·05%) | 0·4% | 1,354 (< 0·05%) | 0·4% | 779 (< 0·05%) | 0·4% | 3,956 (< 0·05%) | 0·3% |
| M71: Other bursopathies | 163 (< 0·05%) | 0·4% | 1,263 (< 0·05%) | 0·3% | 459 (< 0·05%) | 0·2% | 3,316 (< 0·05%) | 0·3% |
| M77: Other enthesopathies | 159 (< 0·05%) | 0·4% | 1,770 (0·1%) | 0·5% | 320 (< 0·05%) | 0·2% | 3,261 (< 0·05%) | 0·3% |
| M11: Other crystal arthropathies | 156 (< 0·05%) | 0·4% | 1,085 (< 0·05%) | 0·3% | 504 (< 0·05%) | 0·3% | 3,587 (< 0·05%) | 0·3% |
| M21: Other acquired deformities of limbs | 151 (< 0·05%) | 0·3% | 1,580 (0·1%) | 0·4% | 990 (< 0·05%) | 0·5% | 8,324 (0·1%) | 0·7% |
| M89: Other disorders of bone | 151 (< 0·05%) | 0·3% | 1,871 (0·1%) | 0·5% | 525 (< 0·05%) | 0·3% | 5,149 (< 0·05%) | 0·4% |
| M05: Seropositive rheumatoid arthritis | 138 (< 0·05%) | 0·3% | 1,142 (< 0·05%) | 0·3% | 623 (< 0·05%) | 0·3% | 3,108 (< 0·05%) | 0·3% |
| M87: Osteonecrosis | 90 (< 0·05%) | 0·2% | 897 (< 0·05%) | 0·2% | 238 (< 0·05%) | 0·1% | 2,228 (< 0·05%) | 0·2% |
| M35: Other systemic involvement of connective tissue | 84 (< 0·05%) | 0·2% | 593 (< 0·05%) | 0·2% | 4,085 (0·1%) | 2·2% | 16,636 (0·1%) | 1·4% |
| M41: Scoliosis | 58 (< 0·05%) | 0·1% | 555 (< 0·05%) | 0·1% | 1,764 (0·1%) | 0·9% | 10,889 (0·1%) | 0·9% |
| M85: Other disorders of bone density and structure | 54 (< 0·05%) | 0·1% | 714 (< 0·05%) | 0·2% | 1,207 (< 0·05%) | 0·6% | 9,995 (0·1%) | 0·9% |
| M88: Paget's disease of bone [osteitis deformans] | 23 (< 0·05%) | 0·1% | 202 (< 0·05%) | 0·1% | 342 (< 0·05%) | 0·2% | 1,909 (< 0·05%) | 0·2% |
| M32: Systemic lupus erythematosus | 19 (< 0·05%) | 0·0% | 98 (< 0·05%) | 0·0% | 354 (< 0·05%) | 0·2% | 1,132 (< 0·05%) | 0·1% |
| M40: Kyphosis and lordosis | 15 (< 0·05%) | 0·0% | 202 (< 0·05%) | 0·1% | 780 (< 0·05%) | 0·4% | 5,286 (< 0·05%) | 0·5% |
| M45: Ankylosing spondylitis | 13 (< 0·05%) | 0·0% | 147 (< 0·05%) | 0·0% | 693 (< 0·05%) | 0·4% | 2,888 (< 0·05%) | 0·3% |
| M07: Psoriatic and enteropathic arthropathies | < 10 (< 0·05%) | 0·0% | 84 (< 0·05%) | 0·0% | 540 (< 0·05%) | 0·3% | 2,502 (< 0·05%) | 0·2% |
| M53: Other dorsopathies, not elsewhere classified | 0 (0%) | 0·0% | 1,304 (< 0·05%) | 0·3% | 0 (0%) | 0·0% | 2,243 (< 0·05%) | 0·2% |
| M66: Spontaneous rupture of synovium and tendon | 0 (0%) | 0·0% | 904 (< 0·05%) | 0·2% | 0 (0%) | 0·0% | 1,314 (< 0·05%) | 0·1% |
| M90: Osteopathies in diseases classified elsewhere | 0 (0%) | 0·0% | 791 (< 0·05%) | 0·2% | 0 (0%) | 0·0% | 3,748 (< 0·05%) | 0·3% |
| M76: Enthesopathies of lower limb, excluding foot | 0 (0%) | 0·0% | 741 (< 0·05%) | 0·2% | 0 (0%) | 0·0% | 1,212 (< 0·05%) | 0·1% |
| M60: Myositis | 0 (0%) | 0·0% | 658 (< 0·05%) | 0·2% | 0 (0%) | 0·0% | 1,722 (< 0·05%) | 0·1% |
| M22: Disorders of patella | 0 (0%) | 0·0% | 353 (< 0·05%) | 0·1% | 0 (0%) | 0·0% | 1,046 (< 0·05%) | 0·1% |
| M49: Spondylopathies in diseases classified elsewhere | 0 (0%) | 0·0% | 140 (< 0·05%) | 0·0% | 0 (0%) | 0·0% | 1,488 (< 0·05%) | 0·1% |
| **N00-N99 Diseases of the genitourinary system** | | | | | | | | |
| N39: Other disorders of urinary system | 14,336 (2·9%) | 39·3% | 106,522 (3·8%) | 38·0% | 50,086 (1·6%) | 24·7% | 265,843 (2·4%) | 26·2% |
| N17: Acute renal failure | 5,244 (1·1%) | 14·4% | 26,167 (0·9%) | 9·3% | 54,494 (1·8%) | 26·9% | 195,016 (1·7%) | 19·2% |
| N20: Calculus of kidney and ureter | 2,055 (0·4%) | 5·6% | 19,860 (0·7%) | 7·1% | 4,203 (0·1%) | 2·1% | 32,161 (0·3%) | 3·2% |
| N40: Hyperplasia of prostate | 1,773 (0·4%) | 4·9% | 14,019 (0·5%) | 5·0% | 14,044 (0·5%) | 6·9% | 74,625 (0·7%) | 7·4% |
| N32: Other disorders of bladder | 1,753 (0·4%) | 4·8% | 14,181 (0·5%) | 5·1% | 5,166 (0·2%) | 2·5% | 41,828 (0·4%) | 4·1% |
| N13: Obstructive and reflux uropathy | 1,340 (0·3%) | 3·7% | 12,421 (0·4%) | 4·4% | 4,160 (0·1%) | 2·1% | 36,150 (0·3%) | 3·6% |
| N35: Urethral stricture | 1,272 (0·3%) | 3·5% | 12,147 (0·4%) | 4·3% | 2,360 (0·1%) | 1·2% | 23,118 (0·2%) | 2·3% |
| N30: Cystitis | 785 (0·2%) | 2·2% | 8,258 (0·3%) | 2·9% | 1,577 (0·1%) | 0·8% | 17,215 (0·2%) | 1·7% |
| N47: Redundant prepuce, phimosis and paraphimosis | 713 (0·1%) | 2·0% | 5,108 (0·2%) | 1·8% | 1,462 (< 0·05%) | 0·7% | 9,565 (0·1%) | 0·9% |
| N81: Female genital prolapse | 542 (0·1%) | 1·5% | 4,971 (0·2%) | 1·8% | 1,270 (< 0·05%) | 0·6% | 9,871 (0·1%) | 1·0% |
| N12: Tubulo-interstitial nephritis, not specified as acute or chronic | 504 (0·1%) | 1·4% | 3,015 (0·1%) | 1·1% | 760 (< 0·05%) | 0·4% | 4,789 (< 0·05%) | 0·5% |
| N45: Orchitis and epididymitis | 484 (0·1%) | 1·3% | 3,199 (0·1%) | 1·1% | 693 (< 0·05%) | 0·3% | 4,585 (< 0·05%) | 0·5% |
| N48: Other disorders of penis | 475 (0·1%) | 1·3% | 3,186 (0·1%) | 1·1% | 1,179 (< 0·05%) | 0·6% | 7,876 (0·1%) | 0·8% |
| N50: Other disorders of male genital organs | 449 (0·1%) | 1·2% | 4,040 (0·1%) | 1·4% | 1,224 (< 0·05%) | 0·6% | 9,675 (0·1%) | 1·0% |
| N21: Calculus of lower urinary tract | 427 (0·1%) | 1·2% | 4,082 (0·1%) | 1·5% | 917 (< 0·05%) | 0·5% | 7,954 (0·1%) | 0·8% |
| N84: Polyp of female genital tract | 420 (0·1%) | 1·2% | 3,780 (0·1%) | 1·3% | 605 (< 0·05%) | 0·3% | 5,533 (< 0·05%) | 0·5% |
| N95: Menopausal and other perimenopausal disorders | 414 (0·1%) | 1·1% | 2,730 (0·1%) | 1·0% | 888 (< 0·05%) | 0·4% | 6,222 (0·1%) | 0·6% |
| N43: Hydrocele and spermatocele | 397 (0·1%) | 1·1% | 2,745 (0·1%) | 1·0% | 835 (< 0·05%) | 0·4% | 5,690 (0·1%) | 0·6% |
| N18: Chronic kidney disease | 384 (0·1%) | 1·1% | 2,613 (0·1%) | 0·9% | 33,370 (1·1%) | 16·4% | 121,998 (1·1%) | 12·0% |
| N28: Other disorders of kidney and ureter, not elsewhere classified | 343 (0·1%) | 0·9% | 2,357 (0·1%) | 0·8% | 5,449 (0·2%) | 2·7% | 29,463 (0·3%) | 2·9% |
| N23: Unspecified renal colic | 285 (0·1%) | 0·8% | 2,160 (0·1%) | 0·8% | 392 (< 0·05%) | 0·2% | 2,842 (< 0·05%) | 0·3% |
| N42: Other disorders of prostate | 231 (< 0·05%) | 0·6% | 2,034 (0·1%) | 0·7% | 919 (< 0·05%) | 0·5% | 7,266 (0·1%) | 0·7% |
| N41: Inflammatory diseases of prostate | 217 (< 0·05%) | 0·6% | 2,236 (0·1%) | 0·8% | 561 (< 0·05%) | 0·3% | 5,382 (< 0·05%) | 0·5% |
| N92: Excessive, frequent and irregular menstruation | 196 (< 0·05%) | 0·5% | 1,108 (< 0·05%) | 0·4% | 446 (< 0·05%) | 0·2% | 2,214 (< 0·05%) | 0·2% |
| N02: Recurrent and persistent haematuria | 193 (< 0·05%) | 0·5% | 1,131 (< 0·05%) | 0·4% | 383 (< 0·05%) | 0·2% | 2,385 (< 0·05%) | 0·2% |
| N83: Noninflammatory disorders of ovary, fallopian tube and broad ligament | 172 (< 0·05%) | 0·5% | 1,346 (< 0·05%) | 0·5% | 710 (< 0·05%) | 0·3% | 5,029 (< 0·05%) | 0·5% |
| N93: Other abnormal uterine and vaginal bleeding | 151 (< 0·05%) | 0·4% | 1,028 (< 0·05%) | 0·4% | 627 (< 0·05%) | 0·3% | 2,912 (< 0·05%) | 0·3% |
| N99: Postprocedural disorders of genitourinary system, not elsewhere classified | 150 (< 0·05%) | 0·4% | 2,078 (0·1%) | 0·7% | 890 (< 0·05%) | 0·4% | 4,285 (< 0·05%) | 0·4% |
| N90: Other noninflammatory disorders of vulva and perineum | 139 (< 0·05%) | 0·4% | 1,116 (< 0·05%) | 0·4% | 296 (< 0·05%) | 0·1% | 2,517 (< 0·05%) | 0·2% |
| N19: Unspecified kidney failure | 124 (< 0·05%) | 0·3% | 935 (< 0·05%) | 0·3% | 6,118 (0·2%) | 3·0% | 17,356 (0·2%) | 1·7% |
| N36: Other disorders of urethra | 119 (< 0·05%) | 0·3% | 1,164 (< 0·05%) | 0·4% | 393 (< 0·05%) | 0·2% | 3,597 (< 0·05%) | 0·4% |
| N85: Other noninflammatory disorders of uterus, except cervix | 118 (< 0·05%) | 0·3% | 1,086 (< 0·05%) | 0·4% | 496 (< 0·05%) | 0·2% | 4,517 (< 0·05%) | 0·4% |
| N31: Neuromuscular dysfunction of bladder, not elsewhere classified | 97 (< 0·05%) | 0·3% | 1,360 (< 0·05%) | 0·5% | 279 (< 0·05%) | 0·1% | 3,266 (< 0·05%) | 0·3% |
| N73: Other female pelvic inflammatory diseases | 49 (< 0·05%) | 0·1% | 465 (< 0·05%) | 0·2% | 273 (< 0·05%) | 0·1% | 3,084 (< 0·05%) | 0·3% |
| N63: Unspecified lump in breast | 40 (< 0·05%) | 0·1% | 345 (< 0·05%) | 0·1% | 397 (< 0·05%) | 0·2% | 1,893 (< 0·05%) | 0·2% |
| N03: Chronic nephritic syndrome | 39 (< 0·05%) | 0·1% | 252 (< 0·05%) | 0·1% | 4,053 (0·1%) | 2·0% | 14,683 (0·1%) | 1·4% |
| N08: Glomerular disorders in diseases classified elsewhere | < 10 (< 0·05%) | 0·0% | 55 (< 0·05%) | 0·0% | 948 (< 0·05%) | 0·5% | 5,056 (< 0·05%) | 0·5% |
| N49: Inflammatory disorders of male genital organs, not elsewhere classified | 0 (0%) | 0·0% | 856 (< 0·05%) | 0·3% | 0 (0%) | 0·0% | 1,527 (< 0·05%) | 0·2% |
| N89: Other noninflammatory disorders of vagina | 0 (0%) | 0·0% | 638 (< 0·05%) | 0·2% | 0 (0%) | 0·0% | 1,954 (< 0·05%) | 0·2% |
| N64: Other disorders of breast | 0 (0%) | 0·0% | 588 (< 0·05%) | 0·2% | 0 (0%) | 0·0% | 1,380 (< 0·05%) | 0·1% |
| N76: Other inflammation of vagina and vulva | 0 (0%) | 0·0% | 488 (< 0·05%) | 0·2% | 0 (0%) | 0·0% | 1,200 (< 0·05%) | 0·1% |
| N94: Pain and other conditions associated with female genital organs and menstrual cycle | 0 (0%) | 0·0% | 426 (< 0·05%) | 0·2% | 0 (0%) | 0·0% | 1,306 (< 0·05%) | 0·1% |
| N60: Benign mammary dysplasia | 0 (0%) | 0·0% | 411 (< 0·05%) | 0·1% | 0 (0%) | 0·0% | 838 (< 0·05%) | 0·1% |
| N04: Nephrotic syndrome | 0 (0%) | 0·0% | 394 (< 0·05%) | 0·1% | 0 (0%) | 0·0% | 1,358 (< 0·05%) | 0·1% |
| N05: Unspecified nephritic syndrome | 0 (0%) | 0·0% | 332 (< 0·05%) | 0·1% | 0 (0%) | 0·0% | 1,532 (< 0·05%) | 0·2% |
| N80: Endometriosis | 0 (0%) | 0·0% | 322 (< 0·05%) | 0·1% | 0 (0%) | 0·0% | 1,538 (< 0·05%) | 0·2% |
| N88: Other noninflammatory disorders of cervix uteri | 0 (0%) | 0·0% | 168 (< 0·05%) | 0·1% | 0 (0%) | 0·0% | 1,336 (< 0·05%) | 0·1% |
| N26: Unspecified contracted kidney | 0 (0%) | 0·0% | 134 (< 0·05%) | 0·0% | 0 (0%) | 0·0% | 1,834 (< 0·05%) | 0·2% |
| N77: Vulvovaginal ulceration and inflammation in diseases classified elsewhere | 0 (0%) | 0·0% | < 10 (< 0·05%) | 0·0% | 0 (0%) | 0·0% | 1,462 (< 0·05%) | 0·1% |

1. Figure S1: The proportion of individuals with a given number* of hospitalisation episodes for the full Hospital Episode Statistics (HES) extract, matched control cohort, and myocardial infarction (MI) cohort


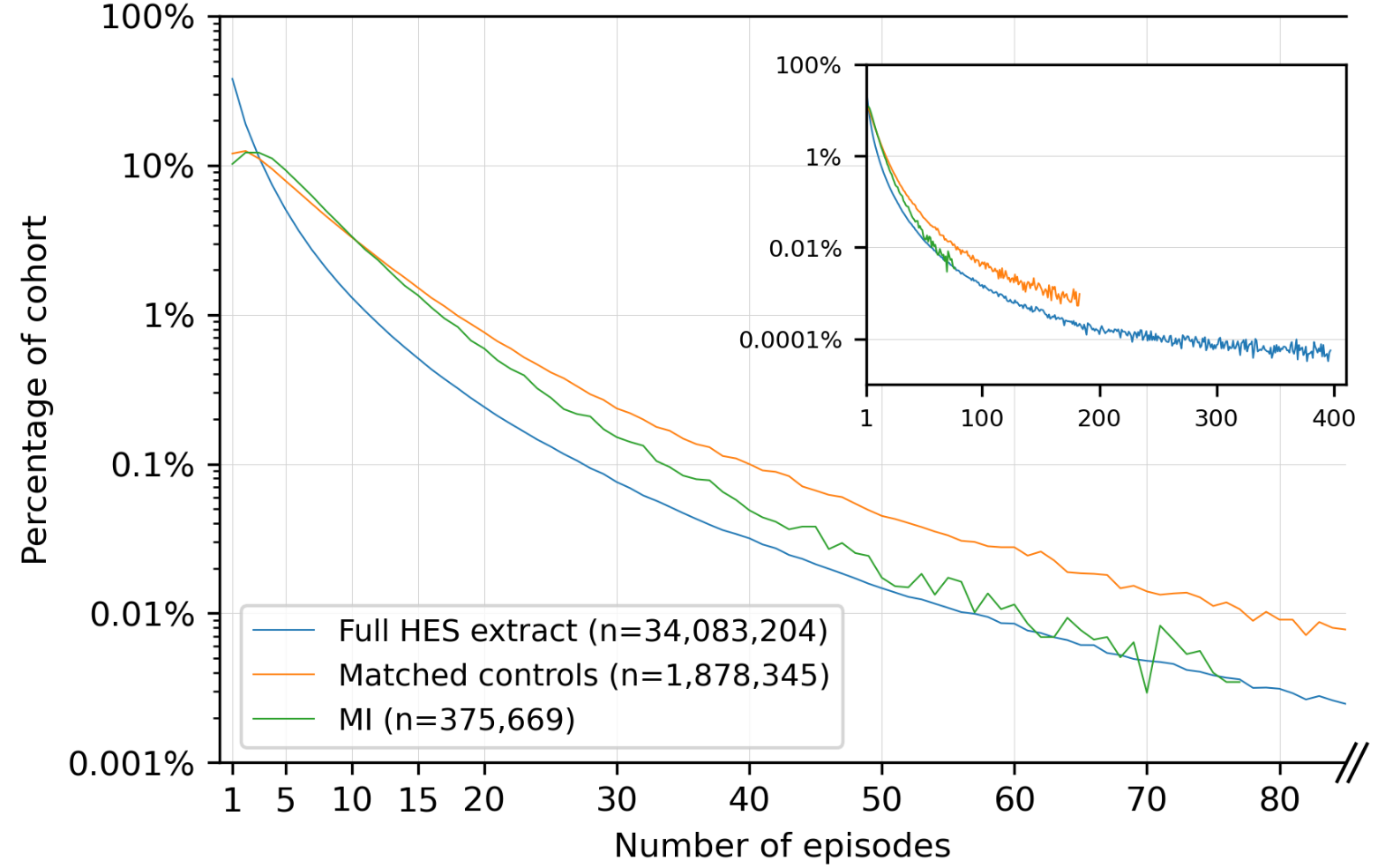


Inset: the full distribution of occurrences, showing the number of episodes for all individuals within the full HES extract. *Data for individuals who have fewer than ten episodes are omitted.

1. Figure S2: The network of disease transitions for temporally sequenced diseases following an initial non-myocardial infarction event, for the matched control cohort, England, 2008-2017





Directed edges between chapter headings represent progression of individuals from one primary diagnosis to the next, grouped by ICD-10 chapters. Disease trajectories from all individuals within the matched control cohort (n=1,878,345) are used to construct this network. The number of individuals (and percentage of cohort) who progress from one disease chapter to the next is shown when at least 1% of individuals contribute to that progression. Edge colour (ref: colour-bar) and thickness are proportional to the number of individuals that progress from one chapter to another. Index hospitalisation event is defined as the first primary diagnoses (non-MI) at the time of matching. An interactive version of this figure – showing counts for all connections; the network for the secondary analyses; and granular sub-chapter transitions – is available at <https://multimorbidity-research-leeds.github.io/>.

Figure S3: Landmark analysis – Disease trajectories† of primary hospitalisation cause for adults with myocardial infarction (MI) in England, compared with an age, sex, and year matched control cohort, 2008-2017


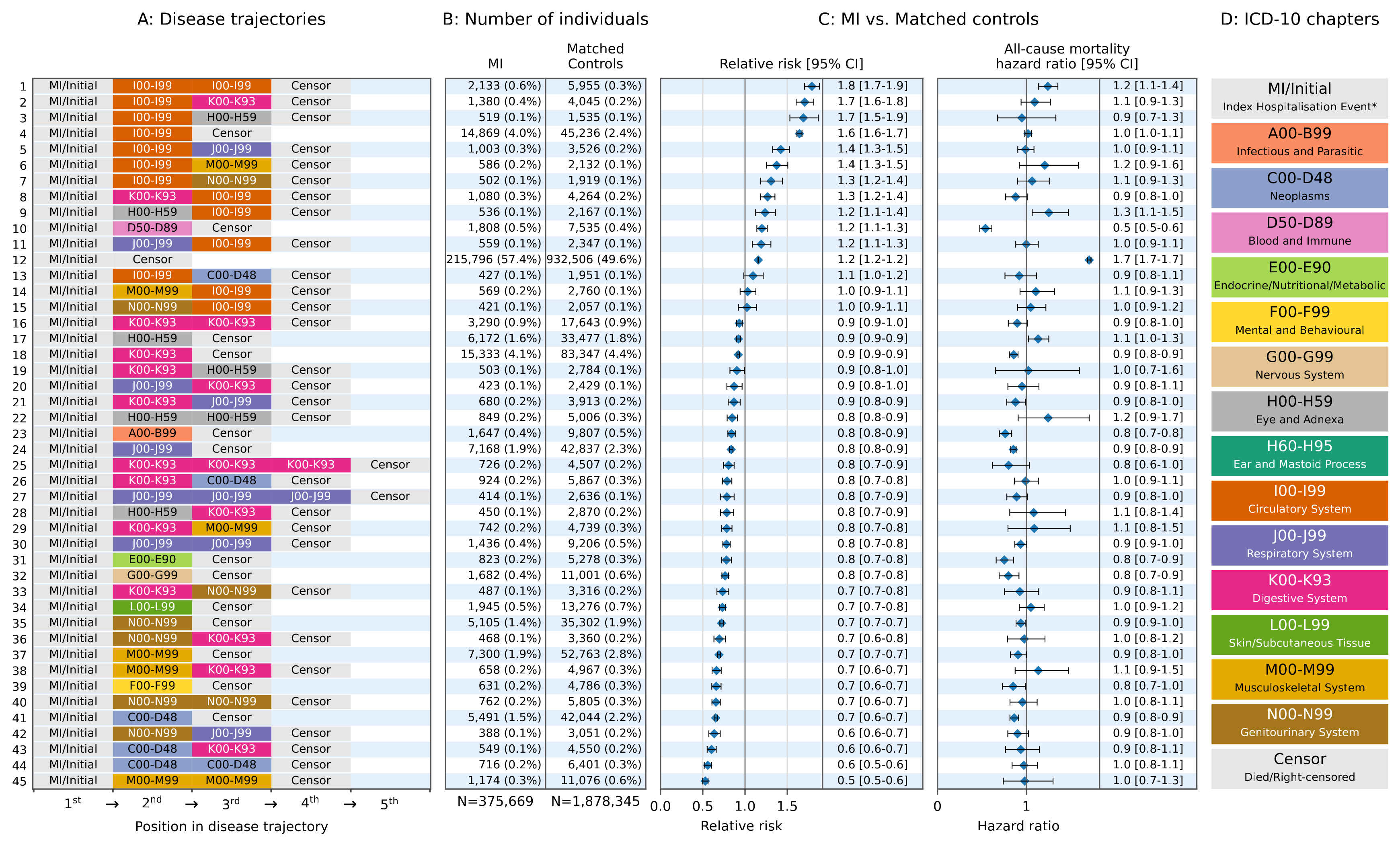


**A:** Chronological disease trajectories including primary diagnoses codes according to ICD-10 chapter headings – trajectories capture only the hospitalisation episodes which appear at least six months after the Index Hospitalisation Event. **B:** Number of MI cases and matched controls following each trajectory. **C:** The relative risk and 95% confidence intervals of disease trajectories between MI and matched control cases, and hazard ratios – all-cause mortality calculated using time-to-event models adjusted for age, sex, year, and deprivation, and accounting for cases nested within hospital trusts using random effects. **D:** ICD-10 chapter headings. An interactive version of this figure – showing a breakdown of disease counts within chapters – is available at <https://multimorbidity-research-leeds.github.io/>. *Index Hospitalisation Event refers to the initial MI diagnoses for the MI cohort and the first primary diagnoses at the time of matching for the matched control cohort. †Only disease trajectories which were followed by at least 0·1% of the MI cohort are shown.

Figure S4: Using an alternative ordering for tied admissions – Disease trajectories† of primary hospitalisation cause for adults with myocardial infarction (MI) in England, compared with an age, sex, and year matched control cohort, 2008-2017


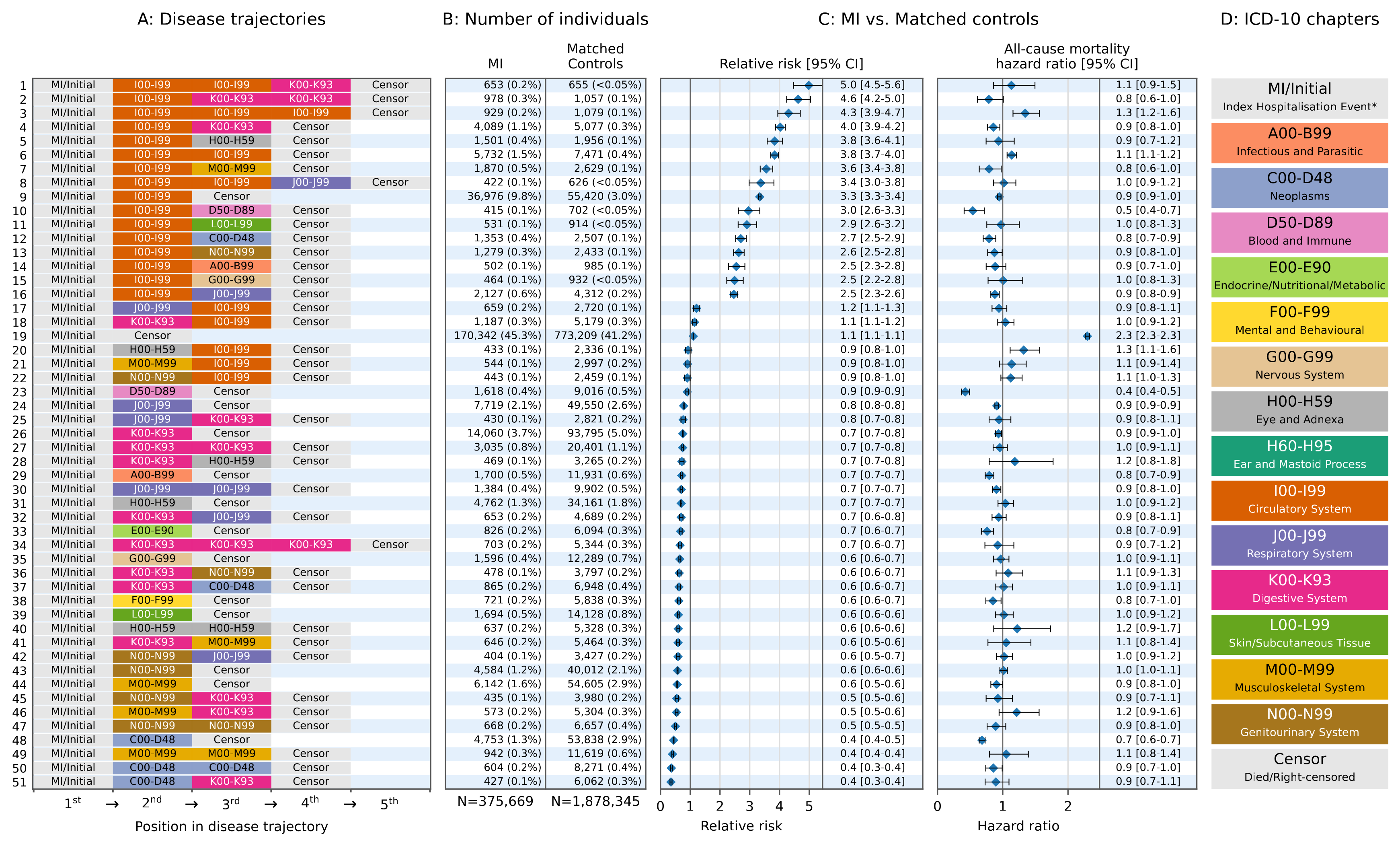


**A:** Chronological disease trajectories including primary diagnoses codes according to ICD-10 chapter headings – a different random order is used for tied admissions. **B:** Number of MI cases and matched controls following each trajectory. **C:** The relative risk and 95% confidence intervals of disease trajectories between MI and matched control cases, and hazard ratios – all-cause mortality calculated using time-to-event models adjusted for age, sex, year, and deprivation, and accounting for cases nested within hospital trusts using random effects. **D:** ICD-10 chapter headings. An interactive version of this figure – showing a breakdown of disease counts within chapters – is available at <https://multimorbidity-research-leeds.github.io/>. *Index Hospitalisation Event refers to the initial MI diagnoses for the MI cohort and the first primary diagnoses at the time of matching for the matched control cohort. †Only disease trajectories which were followed by at least 0·1% of the MI cohort are shown.

Figure S5: For males only – Disease trajectories† of primary hospitalisation cause for adults with myocardial infarction (MI) in England, compared with an age, sex, and year matched control cohort, 2008-2017


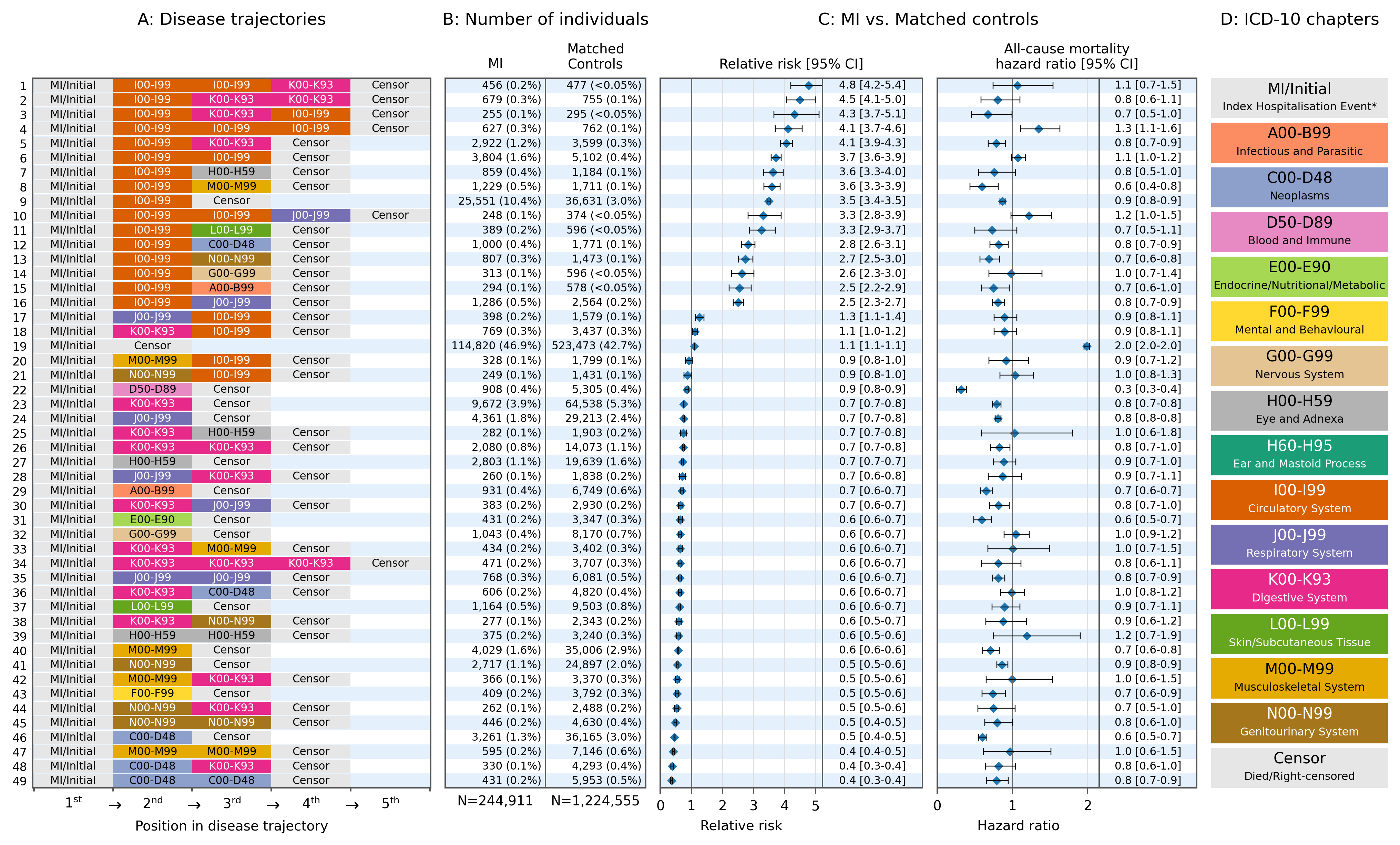


**A:** Chronological disease trajectories including primary diagnoses codes according to ICD-10 chapter headings – for males only. **B:** Number of MI cases and matched controls following each trajectory. **C:** The relative risk and 95% confidence intervals of disease trajectories between MI and matched control cases, and hazard ratios – all-cause mortality calculated using time-to-event models adjusted for age, year, and deprivation, and accounting for cases nested within hospital trusts using random effects. **D:** ICD-10 chapter headings. An interactive version of this figure – showing a breakdown of disease counts within chapters – is available at <https://multimorbidity-research-leeds.github.io/>. *Index Hospitalisation Event refers to the initial MI diagnoses for the MI cohort and the first primary diagnoses at the time of matching for the matched control cohort. †Only disease trajectories which were followed by at least 0·1% of the MI cohort are shown.

Figure S6: For females only – Disease trajectories† of primary hospitalisation cause for adults with myocardial infarction (MI) in England, compared with an age, sex, and year matched control cohort, 2008-2017


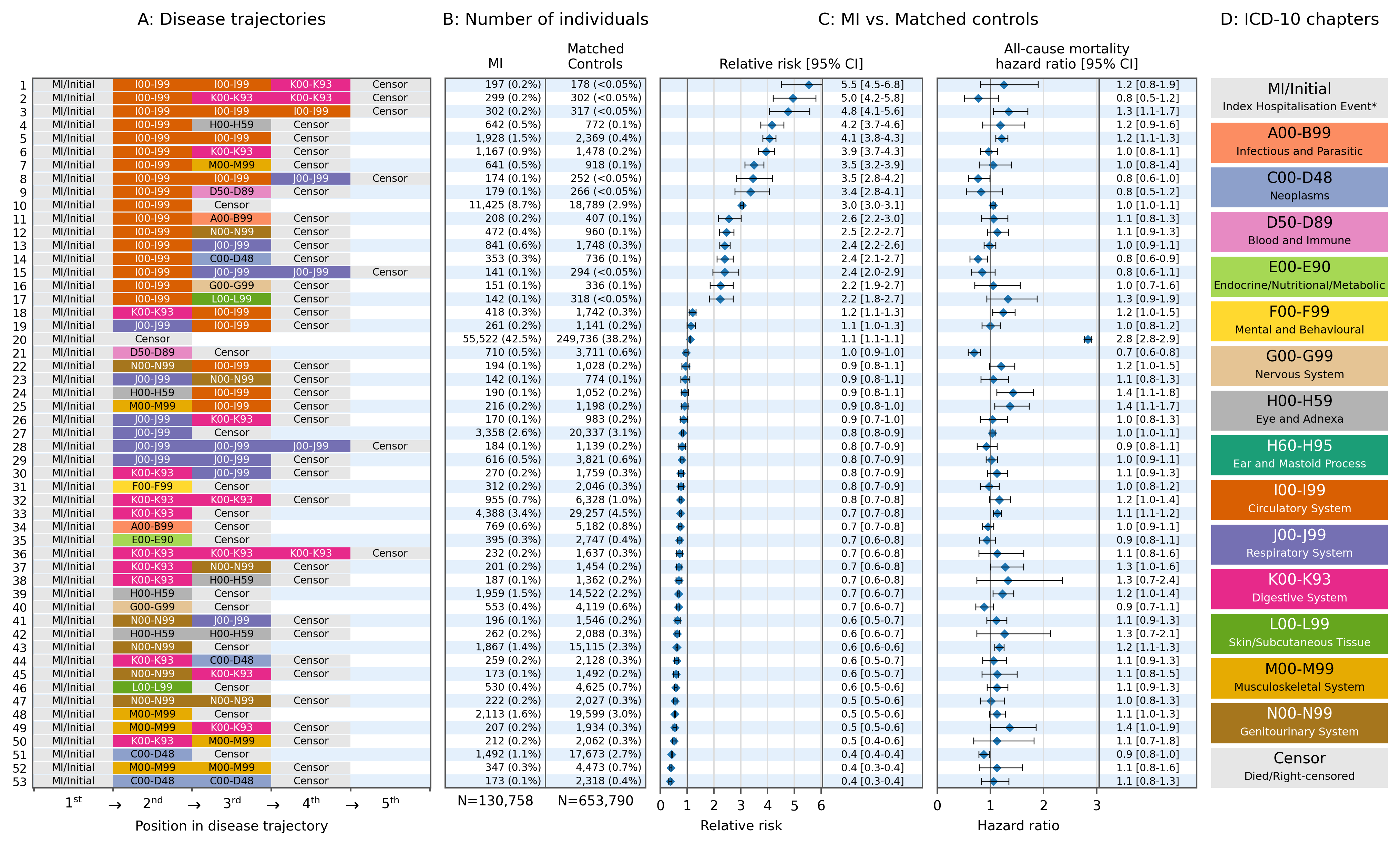


**A:** Chronological disease trajectories including primary diagnoses codes according to ICD-10 chapter headings – for females only. **B:** Number of MI cases and matched controls following each trajectory. **C:** The relative risk and 95% confidence intervals of disease trajectories between MI and matched control cases, and hazard ratios – all-cause mortality calculated using time-to-event models adjusted for age, year, and deprivation, and accounting for cases nested within hospital trusts using random effects. **D:** ICD-10 chapter headings. An interactive version of this figure – showing a breakdown of disease counts within chapters – is available at <https://multimorbidity-research-leeds.github.io/>. *Index Hospitalisation Event refers to the initial MI diagnoses for the MI cohort and the first primary diagnoses at the time of matching for the matched control cohort. †Only disease trajectories which were followed by at least 0·1% of the MI cohort are shown.

Figure S7: Restricted Mean Survival Time (RMST) for diseases trajectories† primary hospitalisation cause for adults with myocardial infarction (MI) in England, and for an age, sex, and year matched control cohort, 2008-2017


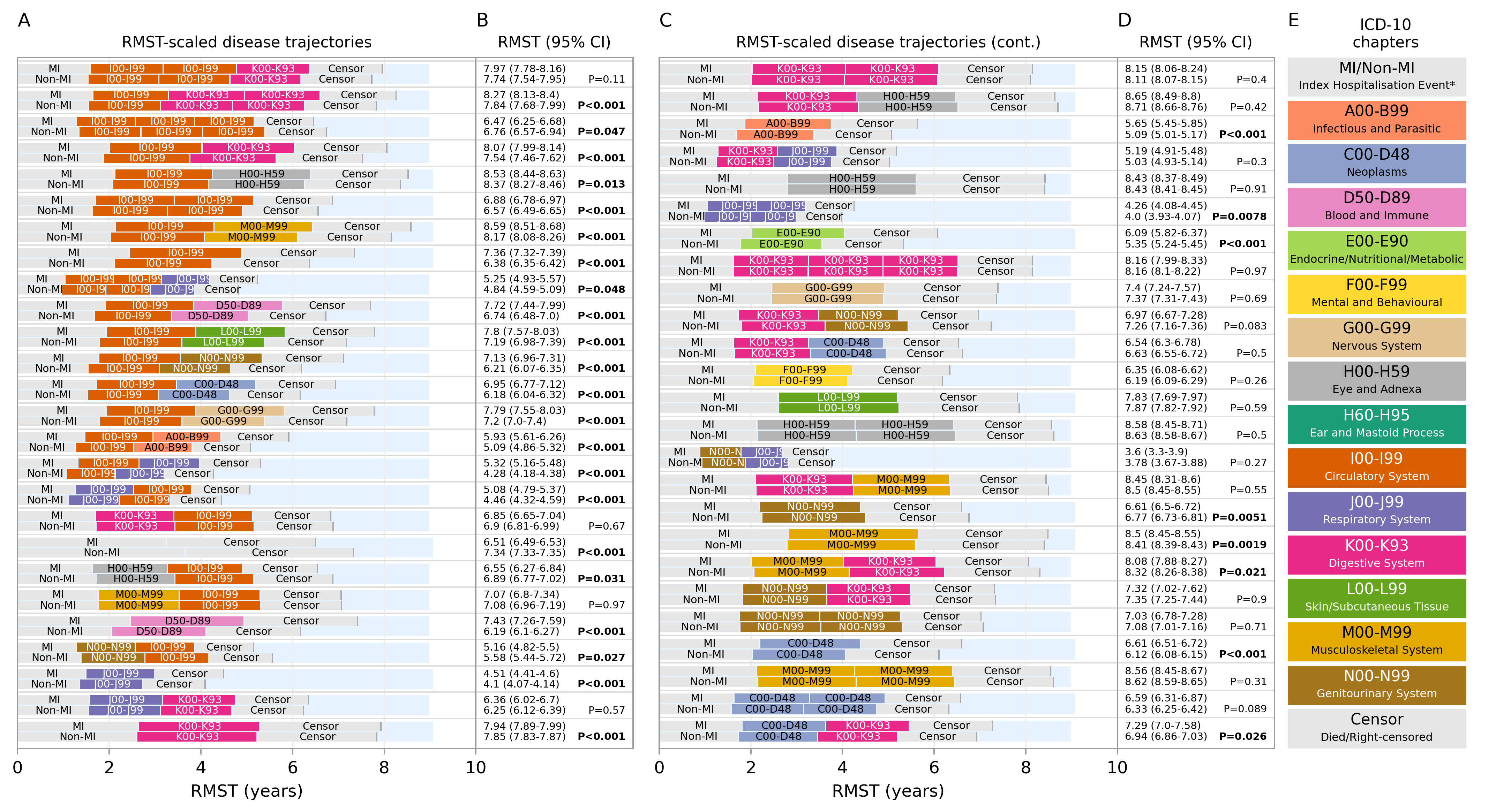


**A/C:** Disease trajectories scaled in length by their RMST, per cohort. Tau – the minimum of the longest follow-up time in the two cohorts – which truncates time, is shown as a light blue background bar for each trajectory. For the corresponding disease trajectory: **B/D:** RMST values for MI and control cohorts. **E:** Disease categories. P-values are calculated using a chi-squared test based on the difference in RMST between cohorts. Uniform scaling is applied to ICD-10 chapters within trajectories (the horizontal length of the whole trajectory conveys the RMST for that trajectory). All error bars are 95% CI. †Disease trajectories correspond to those displayed in Figure 3 (manuscript). *Index hospitalisation event is an MI event for the MI-cohort, and any non-MI event for the control cohort.

Figure S8: Using an alternative ordering for tied admissions – Disease trajectories† of primary and secondary hospitalisation cause for adults with myocardial infarction (MI) in England, compared with an age, sex, and year matched control cohort, 2008-2017


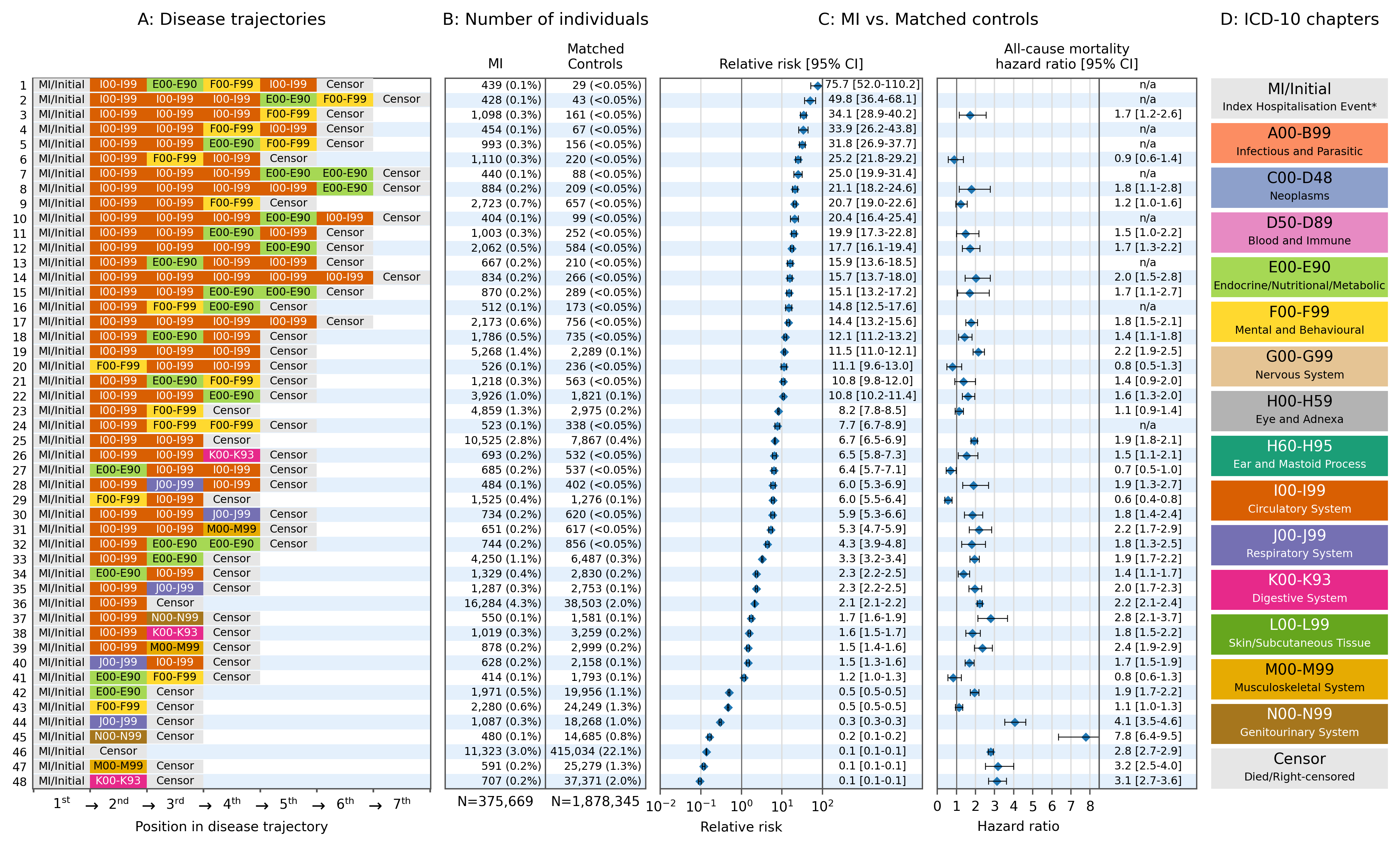


**A:** Chronological disease trajectories including primary and secondary diagnoses codes according to ICD-10 chapter headings – a different random order is used for tied admissions. **B:** Number of MI cases and matched controls following each trajectory. **C:** The relative risk and 95% confidence intervals of disease trajectories between MI and matched control cases, and hazard ratios – all-cause mortality calculated using time-to-event models adjusted for age, sex, year, and deprivation, and accounting for cases nested within hospital trusts using random effects. **D:** ICD-10 chapter headings. An interactive version of this figure – showing a breakdown of disease counts within chapters – is available at <https://multimorbidity-research-leeds.github.io/>. *Index Hospitalisation Event refers to the initial MI diagnoses for the MI cohort and the first primary/secondary diagnoses at the time of matching for the matched control cohort. †Only disease trajectories which were followed by at least 0·1% of the MI cohort are shown.

Figure S9: For males only – Disease trajectories† of primary and secondary hospitalisation cause for adults with myocardial infarction (MI) in England, compared with an age, sex, and year matched control cohort, 2008-2017


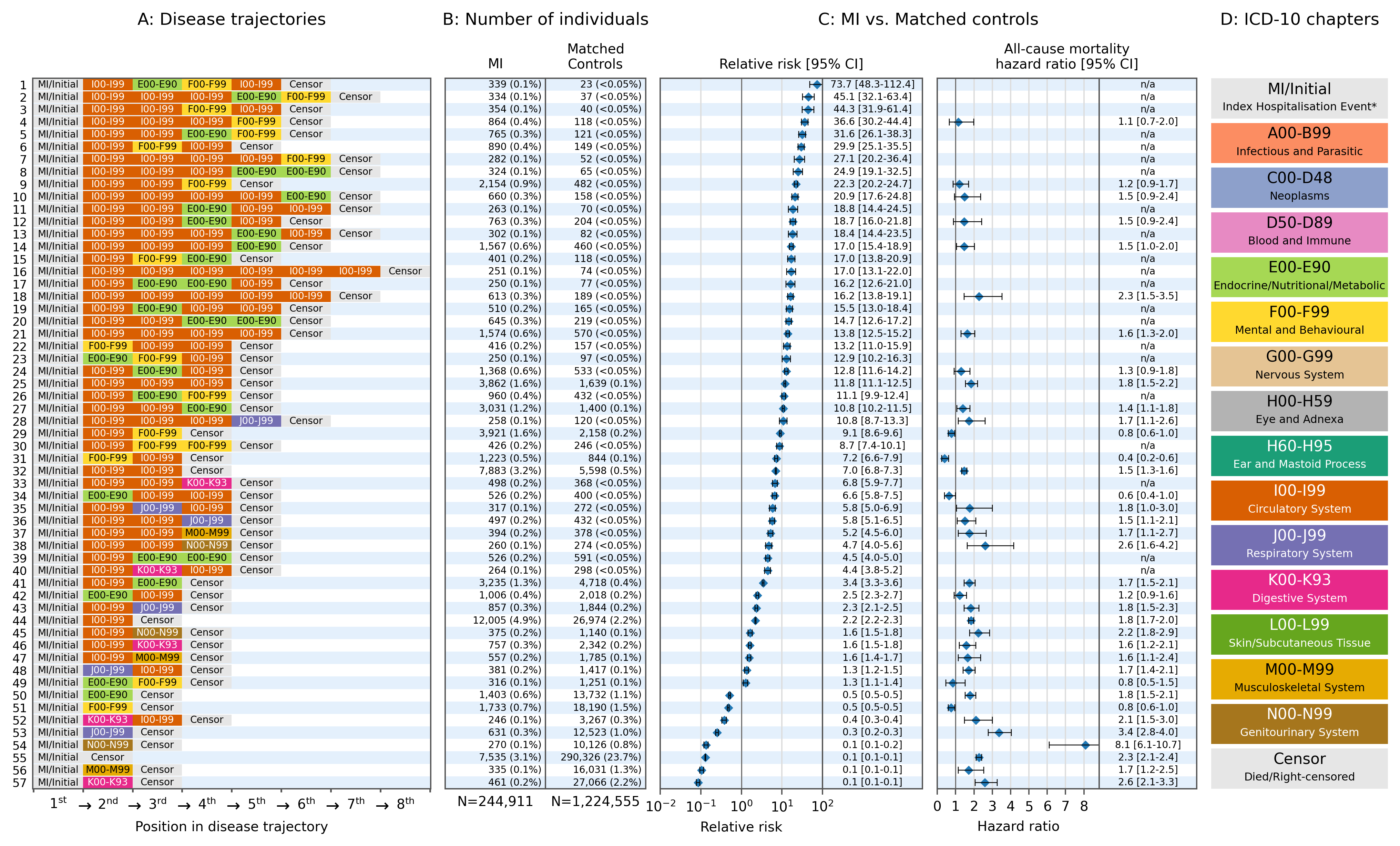


**A:** Chronological disease trajectories including primary and secondary diagnoses codes according to ICD-10 chapter headings – for males only. **B:** Number of MI cases and matched controls following each trajectory. **C:** The relative risk and 95% confidence intervals of disease trajectories between MI and matched control cases, and hazard ratios – all-cause mortality calculated using time-to-event models adjusted for age, year, and deprivation, and accounting for cases nested within hospital trusts using random effects. **D:** ICD-10 chapter headings. An interactive version of this figure – showing a breakdown of disease counts within chapters – is available at <https://multimorbidity-research-leeds.github.io/>. *Index Hospitalisation Event refers to the initial MI diagnoses for the MI cohort and the first primary/secondary diagnoses at the time of matching for the matched control cohort. †Only disease trajectories which were followed by at least 0·1% of the MI cohort are shown.

Figure S10: For females only – Disease trajectories† of primary and secondary hospitalisation cause for adults with myocardial infarction (MI) in England, compared with an age, sex, and year matched control cohort, 2008-2017


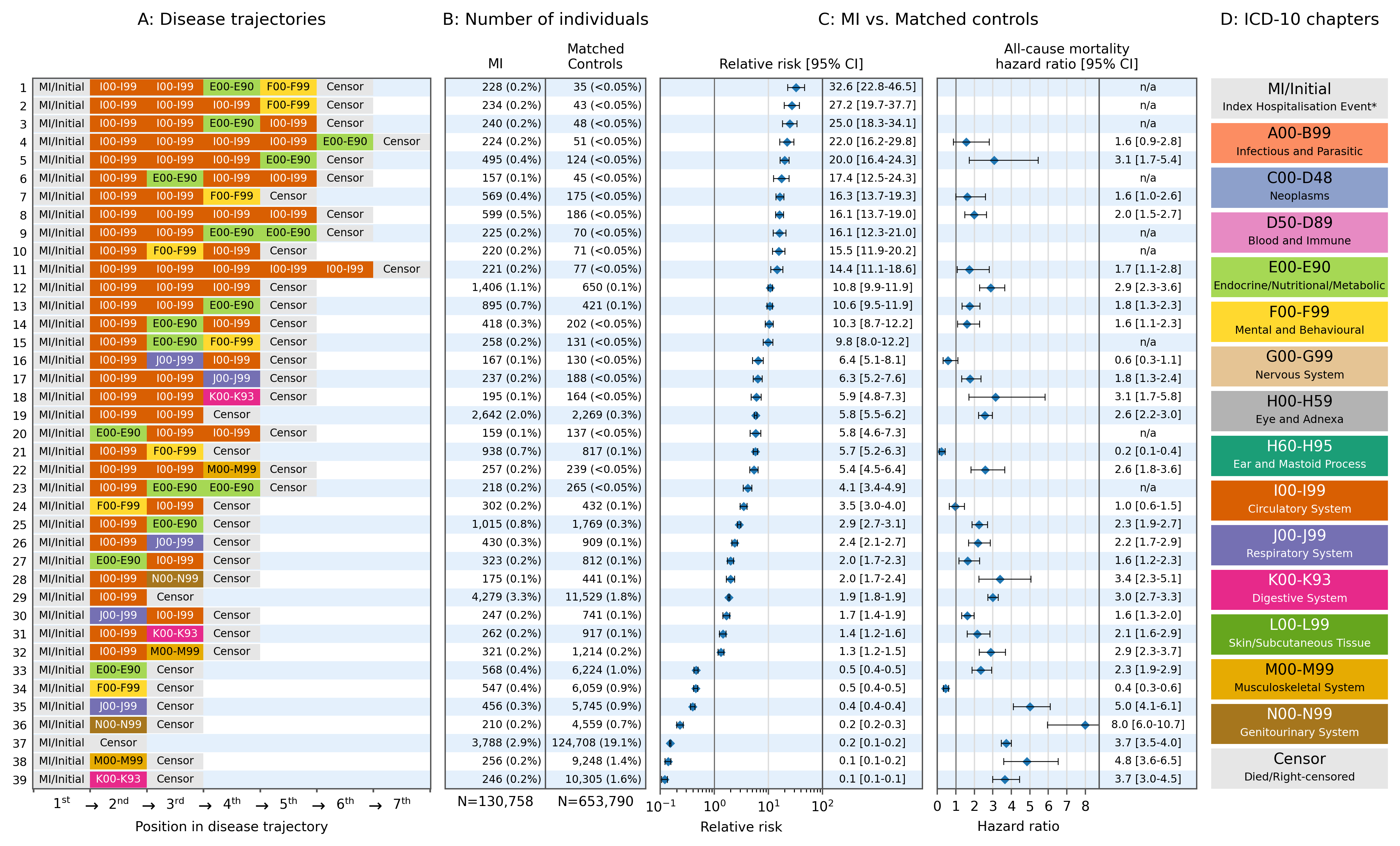


**A:** Chronological disease trajectories including primary and secondary diagnoses codes according to ICD-10 chapter headings – for females only. **B:** Number of MI cases and matched controls following each trajectory. **C:** The relative risk and 95% confidence intervals of disease trajectories between MI and matched control cases, and hazard ratios – all-cause mortality calculated using time-to-event models adjusted for age, year, and deprivation, and accounting for cases nested within hospital trusts using random effects. **D:** ICD-10 chapter headings. An interactive version of this figure – showing a breakdown of disease counts within chapters – is available at <https://multimorbidity-research-leeds.github.io/>. *Index Hospitalisation Event refers to the initial MI diagnoses for the MI cohort and the first primary/secondary diagnoses at the time of matching for the matched control cohort. †Only disease trajectories which were followed by at least 0·1% of the MI cohort are shown.

Figure S11: Restricted Mean Survival Time (RMST) for diseases trajectories† of primary and secondary hospitalisation cause for adults with myocardial infarction (MI) in England, and for an age, sex, and year matched control cohort, 2008-2017


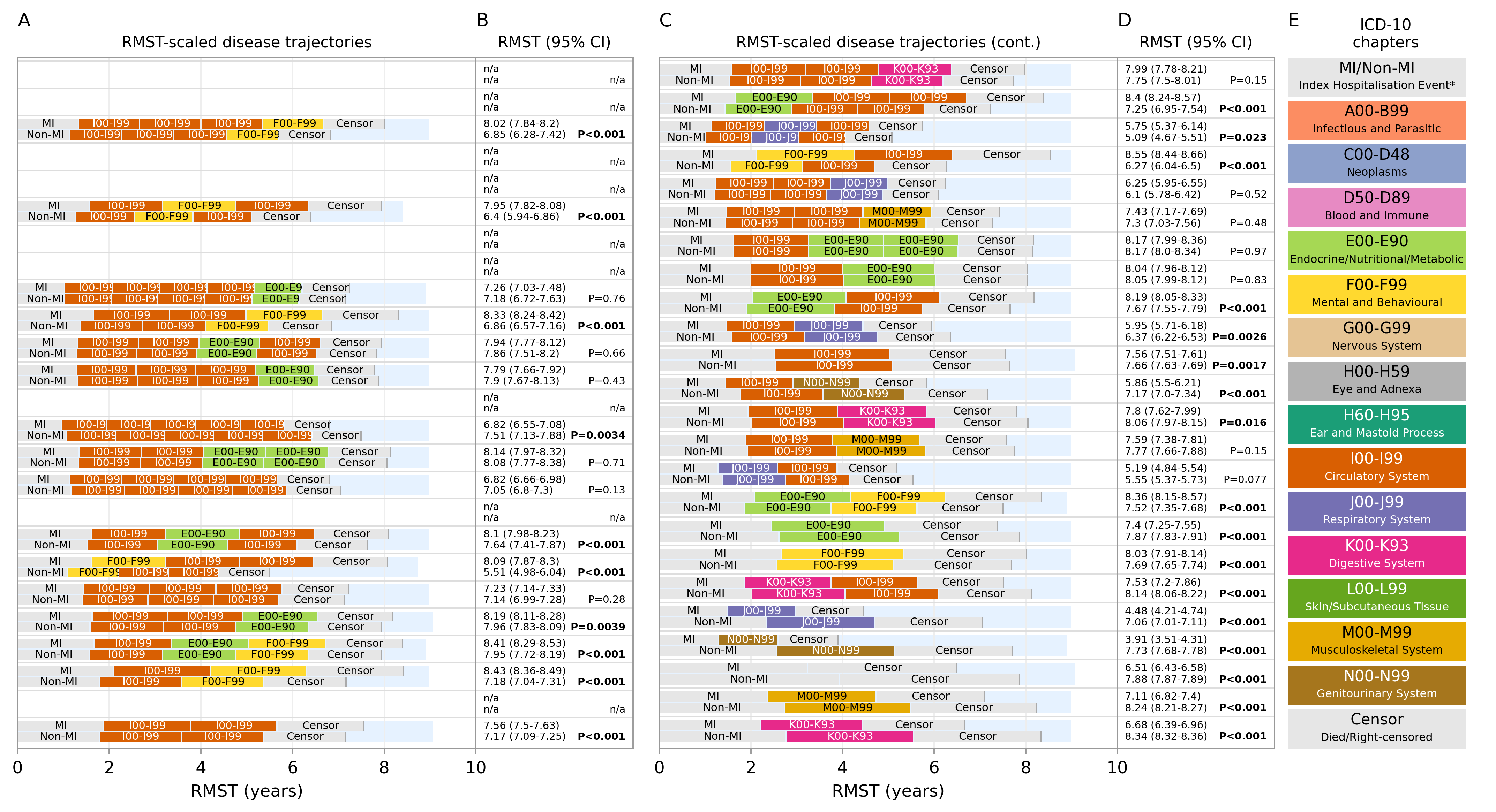


**A/C**: Disease trajectories scaled in length by their RMST, per cohort. Tau – the minimum of the longest follow-up time in the two cohorts – which truncates time, is shown as a light blue background bar for each trajectory. For the corresponding disease trajectory: **B/D**: RMST values for MI and control cohorts. **E**: Disease categories. P-values are calculated using a chi-squared test based on the difference in RMST between cohorts. Uniform scaling is applied to ICD-10 chapters within trajectories (the horizontal length of the whole trajectory conveys the RMST for that trajectory). All error bars are 95% CI. ‘n/a’ is used when the RMST could not be calculated (too few individuals/events in one or both cohorts). †Disease trajectories correspond to those displayed in Figure 4 (manuscript). *Index hospitalisation event is an MI event for the MI-cohort, and any non-MI event for the control cohort.

Table S9: ICD-10 three-character codes for diseases (A00-N99) and their descriptions

| ICD10 diagnosis code | Description |
| --- | --- |
| A00-B99 Certain infectious and parasitic diseases | |
| A00 | Cholera |
| A01 | Typhoid and paratyphoid fevers |
| A02 | Other salmonella infections |
| A03 | Shigellosis |
| A04 | Other bacterial intestinal infections |
| A05 | Other bacterial foodborne intoxications, not elsewhere classified |
| A06 | Amoebiasis |
| A07 | Other protozoal intestinal diseases |
| A08 | Viral and other specified intestinal infections |
| A09 | Other gastroenteritis and colitis of infectious and unspecified origin |
| A15 | Respiratory tuberculosis, bacteriologically and histologically confirmed |
| A16 | Respiratory tuberculosis, not confirmed bacteriologically or histologically |
| A17 | Tuberculosis of nervous system |
| A18 | Tuberculosis of other organs |
| A19 | Miliary tuberculosis |
| A20 | Plague |
| A21 | Tularaemia |
| A22 | Anthrax |
| A23 | Brucellosis |
| A24 | Glanders and melioidosis |
| A25 | Rat-bite fevers |
| A26 | Erysipeloid |
| A27 | Leptospirosis |
| A28 | Other zoonotic bacterial diseases, not elsewhere classified |
| A30 | Leprosy [Hansen's disease] |
| A31 | Infection due to other mycobacteria |
| A32 | Listeriosis |
| A33 | Tetanus neonatorum |
| A34 | Obstetrical tetanus |
| A35 | Other tetanus |
| A36 | Diphtheria |
| A37 | Whooping cough |
| A38 | Scarlet fever |
| A39 | Meningococcal infection |
| A40 | Streptococcal sepsis |
| A41 | Other sepsis |
| A42 | Actinomycosis |
| A43 | Nocardiosis |
| A44 | Bartonellosis |
| A46 | Erysipelas |
| A48 | Other bacterial diseases, not elsewhere classified |
| A49 | Bacterial infection of unspecified site |
| A50 | Congenital syphilis |
| A51 | Early syphilis |
| A52 | Late syphilis |
| A53 | Other and unspecified syphilis |
| A54 | Gonococcal infection |
| A55 | Chlamydial lymphogranuloma (venereum) |
| A56 | Other sexually transmitted chlamydial diseases |
| A57 | Chancroid |
| A58 | Granuloma inguinale |
| A59 | Trichomoniasis |
| A60 | Anogenital herpesviral [herpes simplex] infection |
| A63 | Other predominantly sexually transmitted diseases, not elsewhere classified |
| A64 | Unspecified sexually transmitted disease |
| A65 | Nonvenereal syphilis |
| A66 | Yaws |
| A67 | Pinta [carate] |
| A68 | Relapsing fevers |
| A69 | Other spirochaetal infections |
| A70 | Chlamydia psittaci infection |
| A71 | Trachoma |
| A74 | Other diseases caused by chlamydiae |
| A75 | Typhus fever |
| A77 | Spotted fever [tick-borne rickettsioses] |
| A78 | Q fever |
| A79 | Other rickettsioses |
| A80 | Acute poliomyelitis |
| A81 | Atypical virus infections of central nervous system |
| A82 | Rabies |
| A83 | Mosquito-borne viral encephalitis |
| A84 | Tick-borne viral encephalitis |
| A85 | Other viral encephalitis, not elsewhere classified |
| A86 | Unspecified viral encephalitis |
| A87 | Viral meningitis |
| A88 | Other viral infections of central nervous system, not elsewhere classified |
| A89 | Unspecified viral infection of central nervous system |
| A90 | Dengue fever [classical dengue] |
| A91 | Dengue haemorrhagic fever |
| A92 | Other mosquito-borne viral fevers |
| A93 | Other arthropod-borne viral fevers, not elsewhere classified |
| A94 | Unspecified arthropod-borne viral fever |
| A95 | Yellow fever |
| A96 | Arenaviral haemorrhagic fever |
| A98 | Other viral haemorrhagic fevers, not elsewhere classified |
| A99 | Unspecified viral haemorrhagic fever |
| B00 | Herpesviral [herpes simplex] infections |
| B01 | Varicella [chickenpox] |
| B02 | Zoster [herpes zoster] |
| B03 | Smallpox |
| B04 | Monkeypox |
| B05 | Measles |
| B06 | Rubella [German measles] |
| B07 | Viral warts |
| B08 | Other viral infections characterized by skin and mucous membrane lesions, not elsewhere classified |
| B09 | Unspecified viral infection characterized by skin and mucous membrane lesions |
| B15 | Acute hepatitis A |
| B16 | Acute hepatitis B |
| B17 | Other acute viral hepatitis |
| B18 | Chronic viral hepatitis |
| B19 | Unspecified viral hepatitis |
| B20 | Human immunodeficiency virus [HIV] disease resulting in infectious and parasitic diseases |
| B21 | Human immunodeficiency virus [HIV] disease resulting in malignant neoplasms |
| B22 | Human immunodeficiency virus [HIV] disease resulting in other specified diseases |
| B23 | Human immunodeficiency virus [HIV] disease resulting in other conditions |
| B24 | Unspecified human immunodeficiency virus [HIV] disease |
| B25 | Cytomegaloviral disease |
| B26 | Mumps |
| B27 | Infectious mononucleosis |
| B30 | Viral conjunctivitis |
| B33 | Other viral diseases, not elsewhere classified |
| B34 | Viral infection of unspecified site |
| B35 | Dermatophytosis |
| B36 | Other superficial mycoses |
| B37 | Candidiasis |
| B38 | Coccidioidomycosis |
| B39 | Histoplasmosis |
| B40 | Blastomycosis |
| B41 | Paracoccidioidomycosis |
| B42 | Sporotrichosis |
| B43 | Chromomycosis and phaeomycotic abscess |
| B44 | Aspergillosis |
| B45 | Cryptococcosis |
| B46 | Zygomycosis |
| B47 | Mycetoma |
| B48 | Other mycoses, not elsewhere classified |
| B49 | Unspecified mycosis |
| B50 | Plasmodium falciparum malaria |
| B51 | Plasmodium vivax malaria |
| B52 | Plasmodium malariae malaria |
| B53 | Other parasitologically confirmed malaria |
| B54 | Unspecified malaria |
| B55 | Leishmaniasis |
| B56 | African trypanosomiasis |
| B57 | Chagas' disease |
| B58 | Toxoplasmosis |
| B59 | Pneumocystosis (J17·3*) |
| B60 | Other protozoal diseases, not elsewhere classified |
| B64 | Unspecified protozoal disease |
| B65 | Schistosomiasis [bilharziasis] |
| B66 | Other fluke infections |
| B67 | Echinococcosis |
| B68 | Taeniasis |
| B69 | Cysticercosis |
| B70 | Diphyllobothriasis and sparganosis |
| B71 | Other cestode infections |
| B72 | Dracunculiasis |
| B73 | Onchocerciasis |
| B74 | Filariasis |
| B75 | Trichinellosis |
| B76 | Hookworm diseases |
| B77 | Ascariasis |
| B78 | Strongyloidiasis |
| B79 | Trichuriasis |
| B80 | Enterobiasis |
| B81 | Other intestinal helminthiases, not elsewhere classified |
| B82 | Unspecified intestinal parasitism |
| B83 | Other helminthiases |
| B85 | Pediculosis and phthiriasis |
| B86 | Scabies |
| B87 | Myiasis |
| B88 | Other infestations |
| B89 | Unspecified parasitic disease |
| B90 | Sequelae of tuberculosis |
| B91 | Sequelae of poliomyelitis |
| B92 | Sequelae of leprosy |
| B94 | Sequelae of other and unspecified infectious and parasitic diseases |
| B95 | Streptococcus and staphylococcus as the cause of diseases classified to other chapters |
| B96 | Other specified bacterial agents as the cause of diseases classified to other chapters |
| B97 | Viral agents as the cause of diseases classified to other chapters |
| B98 | Other specified infectious agents as the cause of disease classified to other chapters |
| B99 | Other infectious disease |
| C00-D48 Neoplasms | |
| C00 | Malignant neoplasm of lip |
| C01 | Malignant neoplasm of base of tongue |
| C02 | Malignant neoplasm of other and unspecified parts of tongue |
| C03 | Malignant neoplasm of gum |
| C04 | Malignant neoplasm of floor of mouth |
| C05 | Malignant neoplasm of palate |
| C06 | Malignant neoplasm of other and unspecified parts of mouth |
| C07 | Malignant neoplasm of parotid gland |
| C08 | Malignant neoplasm of other and unspecified major salivary glands |
| C09 | Malignant neoplasm of tonsil |
| C10 | Malignant neoplasm of oropharynx |
| C11 | Malignant neoplasm of nasopharynx |
| C12 | Malignant neoplasm of piriform sinus |
| C13 | Malignant neoplasm of hypopharynx |
| C14 | Malignant neoplasm of other and ill-defined sites in the lip, oral cavity and pharynx |
| C15 | Malignant neoplasm of oesophagus |
| C16 | Malignant neoplasm of stomach |
| C17 | Malignant neoplasm of small intestine |
| C18 | Malignant neoplasm of colon |
| C19 | Malignant neoplasm of rectosigmoid junction |
| C20 | Malignant neoplasm of rectum |
| C21 | Malignant neoplasm of anus and anal canal |
| C22 | Malignant neoplasm of liver and intrahepatic bile ducts |
| C23 | Malignant neoplasm of gallbladder |
| C24 | Malignant neoplasm of other and unspecified parts of biliary tract |
| C25 | Malignant neoplasm of pancreas |
| C26 | Malignant neoplasm of other and ill-defined digestive organs |
| C30 | Malignant neoplasm of nasal cavity and middle ear |
| C31 | Malignant neoplasm of accessory sinuses |
| C32 | Malignant neoplasm of larynx |
| C33 | Malignant neoplasm of trachea |
| C34 | Malignant neoplasm of bronchus and lung |
| C37 | Malignant neoplasm of thymus |
| C38 | Malignant neoplasm of heart, mediastinum and pleura |
| C39 | Malignant neoplasm of other and ill-defined sites in the respiratory system and intrathoracic organs |
| C40 | Malignant neoplasm of bone and articular cartilage of limbs |
| C41 | Malignant neoplasm of bone and articular cartilage of other and unspecified sites |
| C43 | Malignant melanoma of skin |
| C44 | Other malignant neoplasms of skin |
| C45 | Mesothelioma |
| C46 | Kaposi's sarcoma |
| C47 | Malignant neoplasm of peripheral nerves and autonomic nervous system |
| C48 | Malignant neoplasm of retroperitoneum and peritoneum |
| C49 | Malignant neoplasm of other connective and soft tissue |
| C50 | Malignant neoplasm of breast |
| C51 | Malignant neoplasm of vulva |
| C52 | Malignant neoplasm of vagina |
| C53 | Malignant neoplasm of cervix uteri |
| C54 | Malignant neoplasm of corpus uteri |
| C55 | Malignant neoplasm of uterus, part unspecified |
| C56 | Malignant neoplasm of ovary |
| C57 | Malignant neoplasm of other and unspecified female genital organs |
| C58 | Malignant neoplasm of placenta |
| C60 | Malignant neoplasm of penis |
| C61 | Malignant neoplasm of prostate |
| C62 | Malignant neoplasm of testis |
| C63 | Malignant neoplasm of other and unspecified male genital organs |
| C64 | Malignant neoplasm of kidney, except renal pelvis |
| C65 | Malignant neoplasm of renal pelvis |
| C66 | Malignant neoplasm of ureter |
| C67 | Malignant neoplasm of bladder |
| C68 | Malignant neoplasm of other and unspecified urinary organs |
| C69 | Malignant neoplasm of eye and adnexa |
| C70 | Malignant neoplasm of meninges |
| C71 | Malignant neoplasm of brain |
| C72 | Malignant neoplasm of spinal cord, cranial nerves and other parts of central nervous system |
| C73 | Malignant neoplasm of thyroid gland |
| C74 | Malignant neoplasm of adrenal gland |
| C75 | Malignant neoplasm of other endocrine glands and related structures |
| C76 | Malignant neoplasm of other and ill-defined sites |
| C77 | Secondary and unspecified malignant neoplasm of lymph nodes |
| C78 | Secondary malignant neoplasm of respiratory and digestive organs |
| C79 | Secondary malignant neoplasm of other and unspecified sites |
| C80 | Malignant neoplasm without specification of site |
| C81 | Hodgkin lymphoma |
| C82 | Follicular lymphoma |
| C83 | Non-follicular lymphoma |
| C84 | Mature T/NK-cell lymphomas |
| C85 | Other and unspecified types of non-Hodgkin lymphoma |
| C86 | Other specified types of T/NK-cell lymphoma |
| C88 | Malignant immunoproliferative diseases |
| C90 | Multiple myeloma and malignant plasma cell neoplasms |
| C91 | Lymphoid leukaemia |
| C92 | Myeloid leukaemia |
| C93 | Monocytic leukaemia |
| C94 | Other leukaemias of specified cell type |
| C95 | Leukaemia of unspecified cell type |
| C96 | Other and unspecified malignant neoplasms of lymphoid, haematopoietic and related tissue |
| C97 | Malignant neoplasms of independent (primary) multiple sites |
| D00 | Carcinoma in situ of oral cavity, oesophagus and stomach |
| D01 | Carcinoma in situ of other and unspecified digestive organs |
| D02 | Carcinoma in situ of middle ear and respiratory system |
| D03 | Melanoma in situ |
| D04 | Carcinoma in situ of skin |
| D05 | Carcinoma in situ of breast |
| D06 | Carcinoma in situ of cervix uteri |
| D07 | Carcinoma in situ of other and unspecified genital organs |
| D09 | Carcinoma in situ of other and unspecified sites |
| D10 | Benign neoplasm of mouth and pharynx |
| D11 | Benign neoplasm of major salivary glands |
| D12 | Benign neoplasm of colon, rectum, anus and anal canal |
| D13 | Benign neoplasm of other and ill-defined parts of digestive system |
| D14 | Benign neoplasm of middle ear and respiratory system |
| D15 | Benign neoplasm of other and unspecified intrathoracic organs |
| D16 | Benign neoplasm of bone and articular cartilage |
| D17 | Benign lipomatous neoplasm |
| D18 | Haemangioma and lymphangioma, any site |
| D19 | Benign neoplasm of mesothelial tissue |
| D20 | Benign neoplasm of soft tissue of retroperitoneum and peritoneum |
| D21 | Other benign neoplasms of connective and other soft tissue |
| D22 | Melanocytic naevi |
| D23 | Other benign neoplasms of skin |
| D24 | Benign neoplasm of breast |
| D25 | Leiomyoma of uterus |
| D26 | Other benign neoplasms of uterus |
| D27 | Benign neoplasm of ovary |
| D28 | Benign neoplasm of other and unspecified female genital organs |
| D29 | Benign neoplasm of male genital organs |
| D30 | Benign neoplasm of urinary organs |
| D31 | Benign neoplasm of eye and adnexa |
| D32 | Benign neoplasm of meninges |
| D33 | Benign neoplasm of brain and other parts of central nervous system |
| D34 | Benign neoplasm of thyroid gland |
| D35 | Benign neoplasm of other and unspecified endocrine glands |
| D36 | Benign neoplasm of other and unspecified sites |
| D37 | Neoplasm of uncertain or unknown behaviour of oral cavity and digestive organs |
| D38 | Neoplasm of uncertain or unknown behaviour of middle ear and respiratory and intrathoracic organs |
| D39 | Neoplasm of uncertain or unknown behaviour of female genital organs |
| D40 | Neoplasm of uncertain or unknown behaviour of male genital organs |
| D41 | Neoplasm of uncertain or unknown behaviour of urinary organs |
| D42 | Neoplasm of uncertain or unknown behaviour of meninges |
| D43 | Neoplasm of uncertain or unknown behaviour of brain and central nervous system |
| D44 | Neoplasm of uncertain or unknown behaviour of endocrine glands |
| D45 | Polycythaemia vera |
| D46 | Myelodysplastic syndromes |
| D47 | Other neoplasms of uncertain or unknown behaviour of lymphoid, haematopoietic and related tissue |
| D48 | Neoplasm of uncertain or unknown behaviour of other and unspecified sites |
| D50-D89 diseases of the blood and blood-forming organs and certain disorders involving the immune mechanism | |
| D50 | Iron deficiency anaemia |
| D51 | Vitamin B12 deficiency anaemia |
| D52 | Folate deficiency anaemia |
| D53 | Other nutritional anaemias |
| D55 | Anaemia due to enzyme disorders |
| D56 | Thalassaemia |
| D57 | Sickle-cell disorders |
| D58 | Other hereditary haemolytic anaemias |
| D59 | Acquired haemolytic anaemia |
| D60 | Acquired pure red cell aplasia [erythroblastopenia] |
| D61 | Other aplastic anaemias |
| D62 | Acute posthaemorrhagic anaemia |
| D63 | Anaemia in chronic diseases classified elsewhere |
| D64 | Other anaemias |
| D65 | Disseminated intravascular coagulation [defibrination syndrome] |
| D66 | Hereditary factor VIII deficiency |
| D67 | Hereditary factor IX deficiency |
| D68 | Other coagulation defects |
| D69 | Purpura and other haemorrhagic conditions |
| D70 | Agranulocytosis |
| D71 | Functional disorders of polymorphonuclear neutrophils |
| D72 | Other disorders of white blood cells |
| D73 | Diseases of spleen |
| D74 | Methaemoglobinaemia |
| D75 | Other diseases of blood and blood-forming organs |
| D76 | Other specified diseases with participation of lymphoreticular and reticulohistiocytic tissue |
| D77 | Other disorders of blood and blood-forming organs in diseases classified elsewhere |
| D80 | Immunodeficiency with predominantly antibody defects |
| D81 | Combined immunodeficiencies |
| D82 | Immunodeficiency associated with other major defects |
| D83 | Common variable immunodeficiency |
| D84 | Other immunodeficiencies |
| D86 | Sarcoidosis |
| D89 | Other disorders involving the immune mechanism, not elsewhere classified |
| E00-E90 Endocrine, nutritional and metabolic diseases | |
| E00 | Congenital iodine-deficiency syndrome |
| E01 | Iodine-deficiency-related thyroid disorders and allied conditions |
| E02 | Subclinical iodine-deficiency hypothyroidism |
| E03 | Other hypothyroidism |
| E04 | Other nontoxic goitre |
| E05 | Thyrotoxicosis [hyperthyroidism] |
| E06 | Thyroiditis |
| E07 | Other disorders of thyroid |
| E10 | Type 1 diabetes mellitus |
| E11 | Type 2 diabetes mellitus |
| E12 | Malnutrition-related diabetes mellitus |
| E13 | Other specified diabetes mellitus |
| E14 | Unspecified diabetes mellitus |
| E15 | Nondiabetic hypoglycaemic coma |
| E16 | Other disorders of pancreatic internal secretion |
| E20 | Hypoparathyroidism |
| E21 | Hyperparathyroidism and other disorders of parathyroid gland |
| E22 | Hyperfunction of pituitary gland |
| E23 | Hypofunction and other disorders of pituitary gland |
| E24 | Cushing's syndrome |
| E25 | Adrenogenital disorders |
| E26 | Hyperaldosteronism |
| E27 | Other disorders of adrenal gland |
| E28 | Ovarian dysfunction |
| E29 | Testicular dysfunction |
| E30 | Disorders of puberty, not elsewhere classified |
| E31 | Polyglandular dysfunction |
| E32 | Diseases of thymus |
| E34 | Other endocrine disorders |
| E35 | Disorders of endocrine glands in diseases classified elsewhere |
| E40 | Kwashiorkor |
| E41 | Nutritional marasmus |
| E42 | Marasmic kwashiorkor |
| E43 | Unspecified severe protein-energy malnutrition |
| E44 | Protein-energy malnutrition of moderate and mild degree |
| E45 | Retarded development following protein-energy malnutrition |
| E46 | Unspecified protein-energy malnutrition |
| E50 | Vitamin A deficiency |
| E51 | Thiamine deficiency |
| E52 | Niacin deficiency [pellagra] |
| E53 | Deficiency of other B group vitamins |
| E54 | Ascorbic acid deficiency |
| E55 | Vitamin D deficiency |
| E56 | Other vitamin deficiencies |
| E58 | Dietary calcium deficiency |
| E59 | Dietary selenium deficiency |
| E60 | Dietary zinc deficiency |
| E61 | Deficiency of other nutrient elements |
| E63 | Other nutritional deficiencies |
| E64 | Sequelae of malnutrition and other nutritional deficiencies |
| E65 | Localized adiposity |
| E66 | Obesity |
| E67 | Other hyperalimentation |
| E68 | Sequelae of hyperalimentation |
| E70 | Disorders of aromatic amino-acid metabolism |
| E71 | Disorders of branched-chain amino-acid metabolism and fatty-acid metabolism |
| E72 | Other disorders of amino-acid metabolism |
| E73 | Lactose intolerance |
| E74 | Other disorders of carbohydrate metabolism |
| E75 | Disorders of sphingolipid metabolism and other lipid storage disorders |
| E76 | Disorders of glycosaminoglycan metabolism |
| E77 | Disorders of glycoprotein metabolism |
| E78 | Disorders of lipoprotein metabolism and other lipidaemias |
| E79 | Disorders of purine and pyrimidine metabolism |
| E80 | Disorders of porphyrin and bilirubin metabolism |
| E83 | Disorders of mineral metabolism |
| E84 | Cystic fibrosis |
| E85 | Amyloidosis |
| E86 | Volume depletion |
| E87 | Other disorders of fluid, electrolyte and acid-base balance |
| E88 | Other metabolic disorders |
| E89 | Postprocedural endocrine and metabolic disorders, not elsewhere classified |
| E90 | Nutritional and metabolic disorders in diseases classified elsewhere |
| F00-F99 Mental and behavioural disorders | |
| F00 | Dementia in Alzheimer's disease |
| F01 | Vascular dementia |
| F02 | Dementia in other diseases classified elsewhere |
| F03 | Unspecified dementia |
| F04 | Organic amnesic syndrome, not induced by alcohol and other psychoactive substances |
| F05 | Delirium, not induced by alcohol and other psychoactive substances |
| F06 | Other mental disorders due to brain damage and dysfunction and to physical disease |
| F07 | Personality and behavioural disorders due to brain disease, damage and dysfunction |
| F09 | Unspecified organic or symptomatic mental disorder |
| F10 | Mental and behavioural disorders due to use of alcohol |
| F11 | Mental and behavioural disorders due to use of opioids |
| F12 | Mental and behavioural disorders due to use of cannabinoids |
| F13 | Mental and behavioural disorders due to use of sedatives or hypnotics |
| F14 | Mental and behavioural disorders due to use of cocaine |
| F15 | Mental and behavioural disorders due to use of other stimulants, including caffeine |
| F16 | Mental and behavioural disorders due to use of hallucinogens |
| F17 | Mental and behavioural disorders due to use of tobacco |
| F18 | Mental and behavioural disorders due to use of volatile solvents |
| F19 | Mental and behavioural disorders due to multiple drug use and use of other psychoactive substances |
| F20 | Schizophrenia |
| F21 | Schizotypal disorder |
| F22 | Persistent delusional disorders |
| F23 | Acute and transient psychotic disorders |
| F24 | Induced delusional disorder |
| F25 | Schizoaffective disorders |
| F28 | Other nonorganic psychotic disorders |
| F29 | Unspecified nonorganic psychosis |
| F30 | Manic episode |
| F31 | Bipolar affective disorder |
| F32 | Depressive episode |
| F33 | Recurrent depressive disorder |
| F34 | Persistent mood [affective] disorders |
| F38 | Other mood [affective] disorders |
| F39 | Unspecified mood [affective] disorder |
| F40 | Phobic anxiety disorders |
| F41 | Other anxiety disorders |
| F42 | Obsessive-compulsive disorder |
| F43 | Reaction to severe stress, and adjustment disorders |
| F44 | Dissociative [conversion] disorders |
| F45 | Somatoform disorders |
| F48 | Other neurotic disorders |
| F50 | Eating disorders |
| F51 | Nonorganic sleep disorders |
| F52 | Sexual dysfunction, not caused by organic disorder or disease |
| F53 | Mental and behavioural disorders associated with the puerperium, not elsewhere classified |
| F54 | Psychological and behavioural factors associated with disorders or diseases classified elsewhere |
| F55 | Abuse of non-dependence-producing substances |
| F59 | Unspecified behavioural syndromes associated with physiological disturbances and physical factors |
| F60 | Specific personality disorders |
| F61 | Mixed and other personality disorders |
| F62 | Enduring personality changes, not attributable to brain damage and disease |
| F63 | Habit and impulse disorders |
| F64 | Gender identity disorders |
| F65 | Disorders of sexual preference |
| F66 | Psychological and behavioural disorders associated with sexual development and orientation |
| F68 | Other disorders of adult personality and behaviour |
| F69 | Unspecified disorder of adult personality and behaviour |
| F70 | Mild mental retardation |
| F71 | Moderate mental retardation |
| F72 | Severe mental retardation |
| F73 | Profound mental retardation |
| F78 | Other mental retardation |
| F79 | Unspecified mental retardation |
| F80 | Specific developmental disorders of speech and language |
| F81 | Specific developmental disorders of scholastic skills |
| F82 | Specific developmental disorder of motor function |
| F83 | Mixed specific developmental disorders |
| F84 | Pervasive developmental disorders |
| F88 | Other disorders of psychological development |
| F89 | Unspecified disorder of psychological development |
| F90 | Hyperkinetic disorders |
| F91 | Conduct disorders |
| F92 | Mixed disorders of conduct and emotions |
| F93 | Emotional disorders with onset specific to childhood |
| F94 | Disorders of social functioning with onset specific to childhood and adolescence |
| F95 | Tic disorders |
| F98 | Other behavioural and emotional disorders with onset usually occurring in childhood and adolescence |
| F99 | Mental disorder, not otherwise specified |
| G00-G99 Diseases of the nervous system | |
| G00 | Bacterial meningitis, not elsewhere classified |
| G01 | Meningitis in bacterial diseases classified elsewhere |
| G02 | Meningitis in other infectious and parasitic diseases classified elsewhere |
| G03 | Meningitis due to other and unspecified causes |
| G04 | Encephalitis, myelitis and encephalomyelitis |
| G05 | Encephalitis, myelitis and encephalomyelitis in diseases classified elsewhere |
| G06 | Intracranial and intraspinal abscess and granuloma |
| G07 | Intracranial and intraspinal abscess and granuloma in diseases classified elsewhere |
| G08 | Intracranial and intraspinal phlebitis and thrombophlebitis |
| G09 | Sequelae of inflammatory diseases of central nervous system |
| G10 | Huntington's disease |
| G11 | Hereditary ataxia |
| G12 | Spinal muscular atrophy and related syndromes |
| G13 | Systemic atrophies primarily affecting central nervous system in diseases classified elsewhere |
| G14 | Postpolio syndrome |
| G20 | Parkinson's disease |
| G21 | Secondary parkinsonism |
| G22 | Parkinsonism in diseases classified elsewhere |
| G23 | Other degenerative diseases of basal ganglia |
| G24 | Dystonia |
| G25 | Other extrapyramidal and movement disorders |
| G26 | Extrapyramidal and movement disorders in diseases classified elsewhere |
| G30 | Alzheimer's disease |
| G31 | Other degenerative diseases of nervous system, not elsewhere classified |
| G32 | Other degenerative disorders of nervous system in diseases classified elsewhere |
| G35 | Multiple sclerosis |
| G36 | Other acute disseminated demyelination |
| G37 | Other demyelinating diseases of central nervous system |
| G40 | Epilepsy |
| G41 | Status epilepticus |
| G43 | Migraine |
| G44 | Other headache syndromes |
| G45 | Transient cerebral ischaemic attacks and related syndromes |
| G46 | Vascular syndromes of brain in cerebrovascular diseases (I60-I67+) |
| G47 | Sleep disorders |
| G50 | Disorders of trigeminal nerve |
| G51 | Facial nerve disorders |
| G52 | Disorders of other cranial nerves |
| G53 | Cranial nerve disorders in diseases classified elsewhere |
| G54 | Nerve root and plexus disorders |
| G55 | Nerve root and plexus compressions in diseases classified elsewhere |
| G56 | Mononeuropathies of upper limb |
| G57 | Mononeuropathies of lower limb |
| G58 | Other mononeuropathies |
| G59 | Mononeuropathy in diseases classified elsewhere |
| G60 | Hereditary and idiopathic neuropathy |
| G61 | Inflammatory polyneuropathy |
| G62 | Other polyneuropathies |
| G63 | Polyneuropathy in diseases classified elsewhere |
| G64 | Other disorders of peripheral nervous system |
| G70 | Myasthenia gravis and other myoneural disorders |
| G71 | Primary disorders of muscles |
| G72 | Other myopathies |
| G73 | Disorders of myoneural junction and muscle in diseases classified elsewhere |
| G80 | Cerebral palsy |
| G81 | Hemiplegia |
| G82 | Paraplegia and tetraplegia |
| G83 | Other paralytic syndromes |
| G90 | Disorders of autonomic nervous system |
| G91 | Hydrocephalus |
| G92 | Toxic encephalopathy |
| G93 | Other disorders of brain |
| G94 | Other disorders of brain in diseases classified elsewhere |
| G95 | Other diseases of spinal cord |
| G96 | Other disorders of central nervous system |
| G97 | Postprocedural disorders of nervous system, not elsewhere classified |
| G98 | Other disorders of nervous system, not elsewhere classified |
| G99 | Other disorders of nervous system in diseases classified elsewhere |
| H00-H59 Eye and adnexa | |
| H00 | Hordeolum and chalazion |
| H01 | Other inflammation of eyelid |
| H02 | Other disorders of eyelid |
| H03 | Disorders of eyelid in diseases classified elsewhere |
| H04 | Disorders of lacrimal system |
| H05 | Disorders of orbit |
| H06 | Disorders of lacrimal system and orbit in diseases classified elsewhere |
| H10 | Conjunctivitis |
| H11 | Other disorders of conjunctiva |
| H13 | Disorders of conjunctiva in diseases classified elsewhere |
| H15 | Disorders of sclera |
| H16 | Keratitis |
| H17 | Corneal scars and opacities |
| H18 | Other disorders of cornea |
| H19 | Disorders of sclera and cornea in diseases classified elsewhere |
| H20 | Iridocyclitis |
| H21 | Other disorders of iris and ciliary body |
| H22 | Disorders of iris and ciliary body in diseases classified elsewhere |
| H25 | Senile cataract |
| H26 | Other cataract |
| H27 | Other disorders of lens |
| H28 | Cataract and other disorders of lens in diseases classified elsewhere |
| H30 | Chorioretinal inflammation |
| H31 | Other disorders of choroid |
| H32 | Chorioretinal disorders in diseases classified elsewhere |
| H33 | Retinal detachments and breaks |
| H34 | Retinal vascular occlusions |
| H35 | Other retinal disorders |
| H36 | Retinal disorders in diseases classified elsewhere |
| H40 | Glaucoma |
| H42 | Glaucoma in diseases classified elsewhere |
| H43 | Disorders of vitreous body |
| H44 | Disorders of globe |
| H45 | Disorders of vitreous body and globe in diseases classified elsewhere |
| H46 | Optic neuritis |
| H47 | Other disorders of optic [2nd] nerve and visual pathways |
| H48 | Disorders of optic [2nd] nerve and visual pathways in diseases classified elsewhere |
| H49 | Paralytic strabismus |
| H50 | Other strabismus |
| H51 | Other disorders of binocular movement |
| H52 | Disorders of refraction and accommodation |
| H53 | Visual disturbances |
| H54 | Visual impairment including blindness (binocular or monocular) |
| H55 | Nystagmus and other irregular eye movements |
| H57 | Other disorders of eye and adnexa |
| H58 | Other disorders of eye and adnexa in diseases classified elsewhere |
| H59 | Postprocedural disorders of eye and adnexa, not elsewhere classified |
| H60-H95 Ear and mastoid process | |
| H60 | Otitis externa |
| H61 | Other disorders of external ear |
| H62 | Disorders of external ear in diseases classified elsewhere |
| H65 | Nonsuppurative otitis media |
| H66 | Suppurative and unspecified otitis media |
| H67 | Otitis media in diseases classified elsewhere |
| H68 | Eustachian salpingitis and obstruction |
| H69 | Other disorders of Eustachian tube |
| H70 | Mastoiditis and related conditions |
| H71 | Cholesteatoma of middle ear |
| H72 | Perforation of tympanic membrane |
| H73 | Other disorders of tympanic membrane |
| H74 | Other disorders of middle ear and mastoid |
| H75 | Other disorders of middle ear and mastoid in diseases classified elsewhere |
| H80 | Otosclerosis |
| H81 | Disorders of vestibular function |
| H82 | Vertiginous syndromes in diseases classified elsewhere |
| H83 | Other diseases of inner ear |
| H90 | Conductive and sensorineural hearing loss |
| H91 | Other hearing loss |
| H92 | Otalgia and effusion of ear |
| H93 | Other disorders of ear, not elsewhere classified |
| H94 | Other disorders of ear in diseases classified elsewhere |
| H95 | Postprocedural disorders of ear and mastoid process, not elsewhere classified |
| I00-I99 Circulatory system | |
| I00 | Rheumatic fever without mention of heart involvement |
| I01 | Rheumatic fever with heart involvement |
| I02 | Rheumatic chorea |
| I05 | Rheumatic mitral valve diseases |
| I06 | Rheumatic aortic valve diseases |
| I07 | Rheumatic tricuspid valve diseases |
| I08 | Multiple valve diseases |
| I09 | Other rheumatic heart diseases |
| I10 | Essential (primary) hypertension |
| I11 | Hypertensive heart disease |
| I12 | Hypertensive renal disease |
| I13 | Hypertensive heart and renal disease |
| I15 | Secondary hypertension |
| I20 | Angina pectoris |
| I21 | Acute myocardial infarction |
| I22 | Subsequent myocardial infarction |
| I23 | Certain current complications following acute myocardial infarction |
| I24 | Other acute ischaemic heart diseases |
| I25 | Chronic ischaemic heart disease |
| I26 | Pulmonary embolism |
| I27 | Other pulmonary heart diseases |
| I28 | Other diseases of pulmonary vessels |
| I30 | Acute pericarditis |
| I31 | Other diseases of pericardium |
| I32 | Pericarditis in diseases classified elsewhere |
| I33 | Acute and subacute endocarditis |
| I34 | Nonrheumatic mitral valve disorders |
| I35 | Nonrheumatic aortic valve disorders |
| I36 | Nonrheumatic tricuspid valve disorders |
| I37 | Pulmonary valve disorders |
| I38 | Endocarditis, valve unspecified |
| I39 | Endocarditis and heart valve disorders in diseases classified elsewhere |
| I40 | Acute myocarditis |
| I41 | Myocarditis in diseases classified elsewhere |
| I42 | Cardiomyopathy |
| I43 | Cardiomyopathy in diseases classified elsewhere |
| I44 | Atrioventricular and left bundle-branch block |
| I45 | Other conduction disorders |
| I46 | Cardiac arrest |
| I47 | Paroxysmal tachycardia |
| I48 | Atrial fibrillation and flutter |
| I49 | Other cardiac arrhythmias |
| I50 | Heart failure |
| I51 | Complications and ill-defined descriptions of heart disease |
| I52 | Other heart disorders in diseases classified elsewhere |
| I60 | Subarachnoid haemorrhage |
| I61 | Intracerebral haemorrhage |
| I62 | Other nontraumatic intracranial haemorrhage |
| I63 | Cerebral infarction |
| I64 | Stroke, not specified as haemorrhage or infarction |
| I65 | Occlusion and stenosis of precerebral arteries, not resulting in cerebral infarction |
| I66 | Occlusion and stenosis of cerebral arteries, not resulting in cerebral infarction |
| I67 | Other cerebrovascular diseases |
| I68 | Cerebrovascular disorders in diseases classified elsewhere |
| I69 | Sequelae of cerebrovascular disease |
| I70 | Atherosclerosis |
| I71 | Aortic aneurysm and dissection |
| I72 | Other aneurysm and dissection |
| I73 | Other peripheral vascular diseases |
| I74 | Arterial embolism and thrombosis |
| I77 | Other disorders of arteries and arterioles |
| I78 | Diseases of capillaries |
| I79 | Disorders of arteries, arterioles and capillaries in diseases classified elsewhere |
| I80 | Phlebitis and thrombophlebitis |
| I81 | Portal vein thrombosis |
| I82 | Other venous embolism and thrombosis |
| I83 | Varicose veins of lower extremities |
| I84 | Haemorrhoids |
| I85 | Oesophageal varices |
| I86 | Varicose veins of other sites |
| I87 | Other disorders of veins |
| I88 | Nonspecific lymphadenitis |
| I89 | Other noninfective disorders of lymphatic vessels and lymph nodes |
| I95 | Hypotension |
| I97 | Postprocedural disorders of circulatory system, not elsewhere classified |
| I98 | Other disorders of circulatory system in diseases classified elsewhere |
| I99 | Other and unspecified disorders of circulatory system |
| J00-J99 Respiratory system | |
| J00 | Acute nasopharyngitis [common cold] |
| J01 | Acute sinusitis |
| J02 | Acute pharyngitis |
| J03 | Acute tonsillitis |
| J04 | Acute laryngitis and tracheitis |
| J05 | Acute obstructive laryngitis [croup] and epiglottitis |
| J06 | Acute upper respiratory infections of multiple and unspecified sites |
| J09 | Influenza due to certain identified influenza virus |
| J10 | Influenza due to other identified influenza virus |
| J11 | Influenza, virus not identified |
| J12 | Viral pneumonia, not elsewhere classified |
| J13 | Pneumonia due to Streptococcus pneumoniae |
| J14 | Pneumonia due to Haemophilus influenzae |
| J15 | Bacterial pneumonia, not elsewhere classified |
| J16 | Pneumonia due to other infectious organisms, not elsewhere classified |
| J17 | Pneumonia in diseases classified elsewhere |
| J18 | Pneumonia, organism unspecified |
| J20 | Acute bronchitis |
| J21 | Acute bronchiolitis |
| J22 | Unspecified acute lower respiratory infection |
| J30 | Vasomotor and allergic rhinitis |
| J31 | Chronic rhinitis, nasopharyngitis and pharyngitis |
| J32 | Chronic sinusitis |
| J33 | Nasal polyp |
| J34 | Other disorders of nose and nasal sinuses |
| J35 | Chronic diseases of tonsils and adenoids |
| J36 | Peritonsillar abscess |
| J37 | Chronic laryngitis and laryngotracheitis |
| J38 | Diseases of vocal cords and larynx, not elsewhere classified |
| J39 | Other diseases of upper respiratory tract |
| J40 | Bronchitis, not specified as acute or chronic |
| J41 | Simple and mucopurulent chronic bronchitis |
| J42 | Unspecified chronic bronchitis |
| J43 | Emphysema |
| J44 | Other chronic obstructive pulmonary disease |
| J45 | Asthma |
| J46 | Status asthmaticus |
| J47 | Bronchiectasis |
| J60 | Coalworker's pneumoconiosis |
| J61 | Pneumoconiosis due to asbestos and other mineral fibres |
| J62 | Pneumoconiosis due to dust containing silica |
| J63 | Pneumoconiosis due to other inorganic dusts |
| J64 | Unspecified pneumoconiosis |
| J65 | Pneumoconiosis associated with tuberculosis |
| J66 | Airway disease due to specific organic dust |
| J67 | Hypersensitivity pneumonitis due to organic dust |
| J68 | Respiratory conditions due to inhalation of chemicals, gases, fumes and vapours |
| J69 | Pneumonitis due to solids and liquids |
| J70 | Respiratory conditions due to other external agents |
| J80 | Adult respiratory distress syndrome |
| J81 | Pulmonary oedema |
| J82 | Pulmonary eosinophilia, not elsewhere classified |
| J84 | Other interstitial pulmonary diseases |
| J85 | Abscess of lung and mediastinum |
| J86 | Pyothorax |
| J90 | Pleural effusion, not elsewhere classified |
| J91 | Pleural effusion in conditions classified elsewhere |
| J92 | Pleural plaque |
| J93 | Pneumothorax |
| J94 | Other pleural conditions |
| J95 | Postprocedural respiratory disorders, not elsewhere classified |
| J96 | Respiratory failure, not elsewhere classified |
| J98 | Other respiratory disorders |
| J99 | Respiratory disorders in diseases classified elsewhere |
| K00-K93 Digestive system | |
| K00 | Disorders of tooth development and eruption |
| K01 | Embedded and impacted teeth |
| K02 | Dental caries |
| K03 | Other diseases of hard tissues of teeth |
| K04 | Diseases of pulp and periapical tissues |
| K05 | Gingivitis and periodontal diseases |
| K06 | Other disorders of gingiva and edentulous alveolar ridge |
| K07 | Dentofacial anomalies [including malocclusion] |
| K08 | Other disorders of teeth and supporting structures |
| K09 | Cysts of oral region, not elsewhere classified |
| K10 | Other diseases of jaws |
| K11 | Diseases of salivary glands |
| K12 | Stomatitis and related lesions |
| K13 | Other diseases of lip and oral mucosa |
| K14 | Diseases of tongue |
| K20 | Oesophagitis |
| K21 | Gastro-oesophageal reflux disease |
| K22 | Other diseases of oesophagus |
| K23 | Disorders of oesophagus in diseases classified elsewhere |
| K25 | Gastric ulcer |
| K26 | Duodenal ulcer |
| K27 | Peptic ulcer, site unspecified |
| K28 | Gastrojejunal ulcer |
| K29 | Gastritis and duodenitis |
| K30 | Functional dyspepsia |
| K31 | Other diseases of stomach and duodenum |
| K35 | Acute appendicitis |
| K36 | Other appendicitis |
| K37 | Unspecified appendicitis |
| K38 | Other diseases of appendix |
| K40 | Inguinal hernia |
| K41 | Femoral hernia |
| K42 | Umbilical hernia |
| K43 | Ventral hernia |
| K44 | Diaphragmatic hernia |
| K45 | Other abdominal hernia |
| K46 | Unspecified abdominal hernia |
| K50 | Crohn's disease [regional enteritis] |
| K51 | Ulcerative colitis |
| K52 | Other noninfective gastroenteritis and colitis |
| K55 | Vascular disorders of intestine |
| K56 | Paralytic ileus and intestinal obstruction without hernia |
| K57 | Diverticular disease of intestine |
| K58 | Irritable bowel syndrome |
| K59 | Other functional intestinal disorders |
| K60 | Fissure and fistula of anal and rectal regions |
| K61 | Abscess of anal and rectal regions |
| K62 | Other diseases of anus and rectum |
| K63 | Other diseases of intestine |
| K64 | Haemorrhoids and perianal venous thrombosis |
| K65 | Peritonitis |
| K66 | Other disorders of peritoneum |
| K67 | Disorders of peritoneum in infectious diseases classified elsewhere |
| K70 | Alcoholic liver disease |
| K71 | Toxic liver disease |
| K72 | Hepatic failure, not elsewhere classified |
| K73 | Chronic hepatitis, not elsewhere classified |
| K74 | Fibrosis and cirrhosis of liver |
| K75 | Other inflammatory liver diseases |
| K76 | Other diseases of liver |
| K77 | Liver disorders in diseases classified elsewhere |
| K80 | Cholelithiasis |
| K81 | Cholecystitis |
| K82 | Other diseases of gallbladder |
| K83 | Other diseases of biliary tract |
| K85 | Acute pancreatitis |
| K86 | Other diseases of pancreas |
| K87 | Disorders of gallbladder, biliary tract and pancreas in diseases classified elsewhere |
| K90 | Intestinal malabsorption |
| K91 | Postprocedural disorders of digestive system, not elsewhere classified |
| K92 | Other diseases of digestive system |
| K93 | Disorders of other digestive organs in diseases classified elsewhere |
| L00-L99 Skin and subcutaneous tissue | |
| L00 | Staphylococcal scalded skin syndrome |
| L01 | Impetigo |
| L02 | Cutaneous abscess, furuncle and carbuncle |
| L03 | Cellulitis |
| L04 | Acute lymphadenitis |
| L05 | Pilonidal cyst |
| L08 | Other local infections of skin and subcutaneous tissue |
| L10 | Pemphigus |
| L11 | Other acantholytic disorders |
| L12 | Pemphigoid |
| L13 | Other bullous disorders |
| L14 | Bullous disorders in diseases classified elsewhere |
| L20 | Atopic dermatitis |
| L21 | Seborrhoeic dermatitis |
| L22 | Diaper [napkin] dermatitis |
| L23 | Allergic contact dermatitis |
| L24 | Irritant contact dermatitis |
| L25 | Unspecified contact dermatitis |
| L26 | Exfoliative dermatitis |
| L27 | Dermatitis due to substances taken internally |
| L28 | Lichen simplex chronicus and prurigo |
| L29 | Pruritus |
| L30 | Other dermatitis |
| L40 | Psoriasis |
| L41 | Parapsoriasis |
| L42 | Pityriasis rosea |
| L43 | Lichen planus |
| L44 | Other papulosquamous disorders |
| L45 | Papulosquamous disorders in diseases classified elsewhere |
| L50 | Urticaria |
| L51 | Erythema multiforme |
| L52 | Erythema nodosum |
| L53 | Other erythematous conditions |
| L54 | Erythema in diseases classified elsewhere |
| L55 | Sunburn |
| L56 | Other acute skin changes due to ultraviolet radiation |
| L57 | Skin changes due to chronic exposure to nonionizing radiation |
| L58 | Radiodermatitis |
| L59 | Other disorders of skin and subcutaneous tissue related to radiation |
| L60 | Nail disorders |
| L62 | Nail disorders in diseases classified elsewhere |
| L63 | Alopecia areata |
| L64 | Androgenic alopecia |
| L65 | Other nonscarring hair loss |
| L66 | Cicatricial alopecia [scarring hair loss] |
| L67 | Hair colour and hair shaft abnormalities |
| L68 | Hypertrichosis |
| L70 | Acne |
| L71 | Rosacea |
| L72 | Follicular cysts of skin and subcutaneous tissue |
| L73 | Other follicular disorders |
| L74 | Eccrine sweat disorders |
| L75 | Apocrine sweat disorders |
| L80 | Vitiligo |
| L81 | Other disorders of pigmentation |
| L82 | Seborrhoeic keratosis |
| L83 | Acanthosis nigricans |
| L84 | Corns and callosities |
| L85 | Other epidermal thickening |
| L86 | Keratoderma in diseases classified elsewhere |
| L87 | Transepidermal elimination disorders |
| L88 | Pyoderma gangrenosum |
| L89 | Decubitus ulcer and pressure area |
| L90 | Atrophic disorders of skin |
| L91 | Hypertrophic disorders of skin |
| L92 | Granulomatous disorders of skin and subcutaneous tissue |
| L93 | Lupus erythematosus |
| L94 | Other localized connective tissue disorders |
| L95 | Vasculitis limited to skin, not elsewhere classified |
| L97 | Ulcer of lower limb, not elsewhere classified |
| L98 | Other disorders of skin and subcutaneous tissue, not elsewhere classified |
| L99 | Other disorders of skin and subcutaneous tissue in diseases classified elsewhere |
| M00-M99 Musculoskeletal system and connective tissue | |
| M00 | Pyogenic arthritis |
| M01 | Direct infections of joint in infectious and parasitic diseases classified elsewhere |
| M02 | Reactive arthropathies |
| M03 | Postinfective and reactive arthropathies in diseases classified elsewhere |
| M05 | Seropositive rheumatoid arthritis |
| M06 | Other rheumatoid arthritis |
| M07 | Psoriatic and enteropathic arthropathies |
| M08 | Juvenile arthritis |
| M09 | Juvenile arthritis in diseases classified elsewhere |
| M10 | Gout |
| M11 | Other crystal arthropathies |
| M12 | Other specific arthropathies |
| M13 | Other arthritis |
| M14 | Arthropathies in other diseases classified elsewhere |
| M15 | Polyarthrosis |
| M16 | Coxarthrosis [arthrosis of hip] |
| M17 | Gonarthrosis [arthrosis of knee] |
| M18 | Arthrosis of first carpometacarpal joint |
| M19 | Other arthrosis |
| M20 | Acquired deformities of fingers and toes |
| M21 | Other acquired deformities of limbs |
| M22 | Disorders of patella |
| M23 | Internal derangement of knee |
| M24 | Other specific joint derangements |
| M25 | Other joint disorders, not elsewhere classified |
| M30 | Polyarteritis nodosa and related conditions |
| M31 | Other necrotizing vasculopathies |
| M32 | Systemic lupus erythematosus |
| M33 | Dermatopolymyositis |
| M34 | Systemic sclerosis |
| M35 | Other systemic involvement of connective tissue |
| M36 | Systemic disorders of connective tissue in diseases classified elsewhere |
| M40 | Kyphosis and lordosis |
| M41 | Scoliosis |
| M42 | Spinal osteochondrosis |
| M43 | Other deforming dorsopathies |
| M45 | Ankylosing spondylitis |
| M46 | Other inflammatory spondylopathies |
| M47 | Spondylosis |
| M48 | Other spondylopathies |
| M49 | Spondylopathies in diseases classified elsewhere |
| M50 | Cervical disc disorders |
| M51 | Other intervertebral disc disorders |
| M53 | Other dorsopathies, not elsewhere classified |
| M54 | Dorsalgia |
| M60 | Myositis |
| M61 | Calcification and ossification of muscle |
| M62 | Other disorders of muscle |
| M63 | Disorders of muscle in diseases classified elsewhere |
| M65 | Synovitis and tenosynovitis |
| M66 | Spontaneous rupture of synovium and tendon |
| M67 | Other disorders of synovium and tendon |
| M68 | Disorders of synovium and tendon in diseases classified elsewhere |
| M70 | Soft tissue disorders related to use, overuse and pressure |
| M71 | Other bursopathies |
| M72 | Fibroblastic disorders |
| M73 | Soft tissue disorders in diseases classified elsewhere |
| M75 | Shoulder lesions |
| M76 | Enthesopathies of lower limb, excluding foot |
| M77 | Other enthesopathies |
| M79 | Other soft tissue disorders, not elsewhere classified |
| M80 | Osteoporosis with pathological fracture |
| M81 | Osteoporosis without pathological fracture |
| M82 | Osteoporosis in diseases classified elsewhere |
| M83 | Adult osteomalacia |
| M84 | Disorders of continuity of bone |
| M85 | Other disorders of bone density and structure |
| M86 | Osteomyelitis |
| M87 | Osteonecrosis |
| M88 | Paget's disease of bone [osteitis deformans] |
| M89 | Other disorders of bone |
| M90 | Osteopathies in diseases classified elsewhere |
| M91 | Juvenile osteochondrosis of hip and pelvis |
| M92 | Other juvenile osteochondrosis |
| M93 | Other osteochondropathies |
| M94 | Other disorders of cartilage |
| M95 | Other acquired deformities of musculoskeletal system and connective tissue |
| M96 | Postprocedural musculoskeletal disorders, not elsewhere classified |
| M99 | Biomechanical lesions, not elsewhere classified |
| N00-N99 Diseases of the genitourinary system | |
| N00 | Acute nephritic syndrome |
| N01 | Rapidly progressive nephritic syndrome |
| N02 | Recurrent and persistent haematuria |
| N03 | Chronic nephritic syndrome |
| N04 | Nephrotic syndrome |
| N05 | Unspecified nephritic syndrome |
| N06 | Isolated proteinuria with specified morphological lesion |
| N07 | Hereditary nephropathy, not elsewhere classified |
| N08 | Glomerular disorders in diseases classified elsewhere |
| N10 | Acute tubulo-interstitial nephritis |
| N11 | Chronic tubulo-interstitial nephritis |
| N12 | Tubulo-interstitial nephritis, not specified as acute or chronic |
| N13 | Obstructive and reflux uropathy |
| N14 | Drug- and heavy-metal-induced tubulo-interstitial and tubular conditions |
| N15 | Other renal tubulo-interstitial diseases |
| N16 | Renal tubulo-interstitial disorders in diseases classified elsewhere |
| N17 | Acute renal failure |
| N18 | Chronic kidney disease |
| N19 | Unspecified kidney failure |
| N20 | Calculus of kidney and ureter |
| N21 | Calculus of lower urinary tract |
| N22 | Calculus of urinary tract in diseases classified elsewhere |
| N23 | Unspecified renal colic |
| N25 | Disorders resulting from impaired renal tubular function |
| N26 | Unspecified contracted kidney |
| N27 | Small kidney of unknown cause |
| N28 | Other disorders of kidney and ureter, not elsewhere classified |
| N29 | Other disorders of kidney and ureter in diseases classified elsewhere |
| N30 | Cystitis |
| N31 | Neuromuscular dysfunction of bladder, not elsewhere classified |
| N32 | Other disorders of bladder |
| N33 | Bladder disorders in diseases classified elsewhere |
| N34 | Urethritis and urethral syndrome |
| N35 | Urethral stricture |
| N36 | Other disorders of urethra |
| N37 | Urethral disorders in diseases classified elsewhere |
| N39 | Other disorders of urinary system |
| N40 | Hyperplasia of prostate |
| N41 | Inflammatory diseases of prostate |
| N42 | Other disorders of prostate |
| N43 | Hydrocele and spermatocele |
| N44 | Torsion of testis |
| N45 | Orchitis and epididymitis |
| N46 | Male infertility |
| N47 | Redundant prepuce, phimosis and paraphimosis |
| N48 | Other disorders of penis |
| N49 | Inflammatory disorders of male genital organs, not elsewhere classified |
| N50 | Other disorders of male genital organs |
| N51 | Disorders of male genital organs in diseases classified elsewhere |
| N60 | Benign mammary dysplasia |
| N61 | Inflammatory disorders of breast |
| N62 | Hypertrophy of breast |
| N63 | Unspecified lump in breast |
| N64 | Other disorders of breast |
| N70 | Salpingitis and oophoritis |
| N71 | Inflammatory disease of uterus, except cervix |
| N72 | Inflammatory disease of cervix uteri |
| N73 | Other female pelvic inflammatory diseases |
| N74 | Female pelvic inflammatory disorders in diseases classified elsewhere |
| N75 | Diseases of Bartholin's gland |
| N76 | Other inflammation of vagina and vulva |
| N77 | Vulvovaginal ulceration and inflammation in diseases classified elsewhere |
| N80 | Endometriosis |
| N81 | Female genital prolapse |
| N82 | Fistulae involving female genital tract |
| N83 | Noninflammatory disorders of ovary, fallopian tube and broad ligament |
| N84 | Polyp of female genital tract |
| N85 | Other noninflammatory disorders of uterus, except cervix |
| N86 | Erosion and ectropion of cervix uteri |
| N87 | Dysplasia of cervix uteri |
| N88 | Other noninflammatory disorders of cervix uteri |
| N89 | Other noninflammatory disorders of vagina |
| N90 | Other noninflammatory disorders of vulva and perineum |
| N91 | Absent, scanty and rare menstruation |
| N92 | Excessive, frequent and irregular menstruation |
| N93 | Other abnormal uterine and vaginal bleeding |
| N94 | Pain and other conditions associated with female genital organs and menstrual cycle |
| N95 | Menopausal and other perimenopausal disorders |
| N96 | Habitual aborter |
| N97 | Female infertility |
| N98 | Complications associated with artificial fertilization |
| N99 | Postprocedural disorders of genitourinary system, not elsewhere classified |
